# Absence of functional TolC protein causes increased stress response gene expression in *Sinorhizobium meliloti*

**DOI:** 10.1186/1471-2180-10-180

**Published:** 2010-06-23

**Authors:** Mário R Santos, Ana M Cosme, Jörg D Becker, João MC Medeiros, Márcia F Mata, Leonilde M Moreira

**Affiliations:** 1 IBB- Instituto de Biotecnologia e Bioengenharia, Centro de Engenharia Biológica e Química, Instituto Superior Técnico, Av. Rovisco Pais, 1049-001 Lisboa, Portugal; 2 Instituto Gulbenkian de Ciência, Rua da Quinta Grande No 6, 2780-156 Oeiras, Portugal

## Abstract

**Background:**

The TolC protein from *Sinorhizobium meliloti *has previously been demonstrated to be required for establishing successful biological nitrogen fixation symbiosis with *Medicago sativa*. It is also needed in protein and exopolysaccharide secretion and for protection against osmotic and oxidative stresses. Here, the transcriptional profile of free-living *S. meliloti *1021 *tolC *mutant is described as a step toward understanding its role in the physiology of the cell.

**Results:**

Comparison of *tolC *mutant and wild-type strains transcriptomes showed 1177 genes with significantly increased expression while 325 had significantly decreased expression levels. The genes with an increased expression suggest the activation of a cytoplasmic and extracytoplasmic stress responses possibly mediated by the sigma factor RpoH1 and protein homologues of the CpxRA two-component regulatory system of Enterobacteria, respectively. Stress conditions are probably caused by perturbation of the cell envelope. Consistent with gene expression data, biochemical analysis indicates that the *tolC *mutant suffers from oxidative stress. This is illustrated by the elevated enzyme activity levels detected for catalase, superoxide dismutase and glutathione reductase. The observed increase in the expression of genes encoding products involved in central metabolism and transporters for nutrient uptake suggests a higher metabolic rate of the *tolC *mutant. We also demonstrated increased swarming motility in the *tolC *mutant strain. Absence of functional TolC caused decreased expression mainly of genes encoding products involved in nitrogen metabolism and transport.

**Conclusion:**

This work shows how a mutation in the outer membrane protein TolC, common to many bacterial transport systems, affects expression of a large number of genes that act in concert to restore cell homeostasis. This finding further underlines the fundamental role of this protein in *Sinorhizobium meliloti *biology.

## Background

The outer membrane protein TolC belongs to a family of envelope proteins found in Gram-negative bacteria [1] and is essential for the export of a wide range of toxic substances such as antibiotics, dyes, disinfectants and natural substances produced by the hosts, including bile, hormones and defense molecules [2,3]. TolC is also required for export of a range of extracellular proteins such as metalloproteases, α-hemolysins, lipases, enterotoxin II [4], the siderophore enterobactin [5], colicin uptake and secretion [6] and bacteriophage adsorption [7]. The TolC protein from *Escherichia coli *was also suggested as possibly involved in the efflux of not yet determined cellular metabolites [8]. Intracellular metabolite accumulation caused upregulation of several transcription factors including MarA, SoxS and Rob. These in turn upregulate TolC, leading to a decrease in metabolite concentration and restoration of cell homeostasis [8]. TolC family members are also required for colonization and persistence of bacteria in their host organisms. For example, *Erwinia chrysanthemi *[9] and *Xylella fastidiosa *[10]*tolC *mutants were unable to grow *in planta *and their virulence was severely compromised. TolC-deficient strains of *Brucella suis *[11] and *Vibrio cholerae *[12] also displayed an attenuation of infection or colonization in animal models, respectively. The TolC protein of *Salmonella enterica *was shown to be required for efficient adhesion and invasion of epithelial cells and macrophages and to colonize poultry [13,14]. Webber and collaborators [13] demonstrated that *S. enterica *mutants lacking *acrA*, *acrB*, or *tolC *genes encoding an efflux pump showed repression of operons involved in pathogenesis. Operons included chemotaxis, motility and type III secretion system genes, offering a possible explanation for the attenuated pathogenesis of these strains [13].

TolC protein of *Sinorhizobium meliloti*, the symbiotic partner of the leguminous plant *Medicago sativa *was recently characterised [15]. A *S. meliloti tolC *insertion mutant induced none or only very few nodules in *M. sativa *roots. Any nodules formed were brownish-white, non-nitrogen fixing, in contrast to the pink elongated nitrogen fixing nodules formed by wild-type *S. meliloti *1021. The *tolC *gene mutation strongly affected the resistance phenotype to antimicrobial agents of plant origin and induced higher susceptibility to osmotic and oxidative stresses. Analysis of extracellular proteins showed that calcium-binding protein WgeA (formerly ExpE1), endoglycanase ExsH and the putative hemolysin-type calcium-binding protein SMc04171 were secreted in a TolC dependent manner. Another phenotype shown by the *S. meliloti tolC *mutant was absence of exopolysaccharides succinoglycan and galactoglucan from the culture supernatant [15]. Absence of galactoglucan in the *tolC *mutant is explained by the lack of WgeA protein secretion [16], but the contribution of TolC to succinoglycan production is so far not understood. Several phenotypes displayed by the *S. meliloti tolC *mutant strain illustrated the wide importance of this outer membrane protein to cellular functions. To better understand the contribution of TolC protein to *S. meliloti *cell physiology under free-living conditions, we investigated the effect of its inactivation on the transcriptome. Our data point towards an increased expression of genes encoding products involved in stress response, central metabolic pathways, and nutrient uptake transporters in the *tolC *mutant. Genes encoding products involved in nitrogen metabolism, transport and cell division displayed decreased expression.

## Results and Discussion

### Global changes in gene expression associated to a mutation in the *tolC *gene

**Figure 1:**
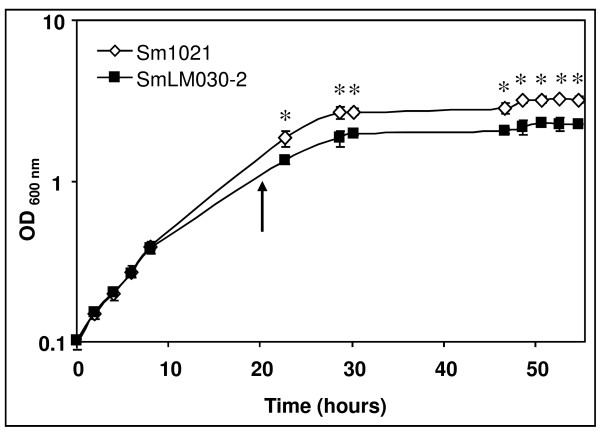
**Effect of *tolC *mutation on growth of *S. meliloti *1021**. Growth curves of *S. meliloti *1021 (◊) and SmLM030-2 *tolC *mutant (■) were obtained in GMS medium. Optical density values are the means of three independent experiments. The arrow indicates the time point where cells were collected for total RNA extraction. Error bars show standard deviations. Asterisks represent data points with significantly different means (p-value < 0.01).

**Figure 2:**
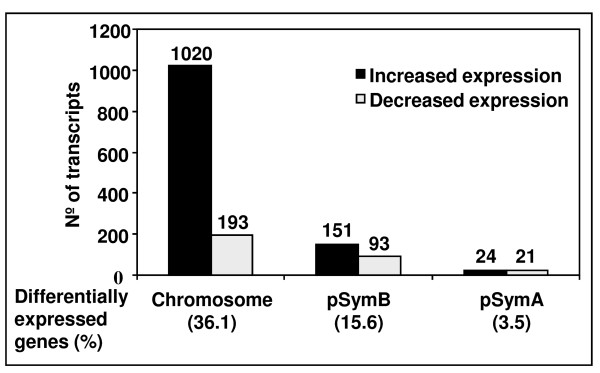
**Distribution of differentially expressed genes in function of the *S. meliloti *1021 replicons**. The histogram shows the number of differentially expressed genes obtained when the *tolC *mutant transcriptome was compared to the wild-type strain and their distribution on the chromosome and the two megaplasmids pSymA and pSymB.

Cosme et al. [15] disrupted the *S. meliloti *1021 *tolC *gene by inserting plasmid pK19mob2ΏHMB into its coding sequence, eliminating the last 102 nucleotides. This mutant, potentially expressing a truncated protein, displayed several phenotypes such as impaired symbiosis with *Medicago*, higher sensitivity to osmotic and oxidative stresses and absence of some extracellular proteins and exopolysaccharides [15]. Here, growth rates of wild-type and the *tolC *gene insertion mutant SmLM030-2 grown in GMS medium were determined (Fig. 1). During the first 8 hours the growth rate was comparable for both strains; subsequently the *tolC *mutant showed a lower growth rate and reduced biomass formation. To gain insight into what underlies these differences, transcriptomes of the wild-type and the *tolC *mutant strains cultured in GMS medium for 20 hours were compared. Microarray data analyzed using dChip (≥1.2-fold change lower confidence bound and a ≤0.4% FDR as cutoffs) and Partek Genomics Suite (FDR ≤ 5%; p-value ≤ 0.017) identified 2067 probe sets in common as being differentially expressed. From this list, we removed duplicated probes for the same genes and those covering intergenic regions, giving a subset of 1809 genes with differential expression (See Additional file 1: Table S1 and Additional file 2: Table S2). Clusters of Orthologous Groups (COGs) could be attributed to 1502 of these according to predicted gene functions (See Additional file 1: Table S1 and Additional file 2: Table S2). Replicon distribution of the 1502 *tolC*-dependent differentially expressed genes indicated that 1213 (80%) were chromosomal, 244 (16%) from pSymB and 45 (3%) from pSymA (Fig. 2). The annotated genome of *S. meliloti *1021 has 54% of genes located in the chromosome, 25% on pSymB and 21% on pSymA. The distribution of *tolC*-dependently expressed genes shows a replicon bias with 1.50-fold higher impact on the chromosome encoded transcripts. Contrastingly, genes from pSymB and pSymA were under-represented with 0.65- and 0.14-fold, respectively.

**Figure 3:**
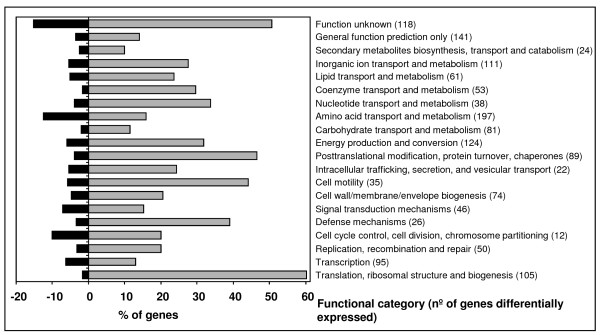
**Distribution of genes with differentially altered expression into COGs**. Effect of the *tolC *gene mutation on the *S. meliloti *transcriptome analyzed according to the distribution of the genes with altered expression into 20 functional categories (COGs) as predicted using NCBI database. The black and grey bars represent the percentage of genes in each functional category whose transcription was decreased and increased, respectively, in the *tolC *mutant SmLM030-2 by comparison to the wild-type strain 1021.

A total of 1177 genes (Table 1 and Additional file 1: Table S1) had significantly increased expression in the *tolC *mutant. These could be classified in 20 functional categories. Fig. 3 summarizes the percentages of differentially expressed genes in comparison to genes of the same category represented on the microarray. The largest categories, with more than 30% of the genes with significantly increased expression, included genes involved into protein synthesis, defense, cell motility, protein modification and turnover, energy production, nucleotide metabolism, and genes of unknown function (Fig. 3, grey bars). Microarray analysis revealed that expression of 325 genes was significantly decreased in the *tolC *mutant (Table 2 and Additional file 2: Table S2). Largest categories, with more than 10% of the genes with a significantly decreased expression include the genes involved in cell division, amino acid transport and metabolism, and of unknown function (Fig. 3, black bars).

**Table 1:** Genes with more than 8-fold increased expression in the *tolC *mutant strain.

Gene identifier	Annotation or description	**Fold change**^**1 **^**(*tolC *vs. wild-type)**
Signal transduction		
		
SMb21560	Putative two-component sensor histidine kinase	14.7
SMb21561	Putative two-component response regulator	27.1
		
Translation		
		
SMc00320	*rbfA *probable ribosome-binding factor A, rRNA processing protein	8.9
SMc00323	*rpsO *robable 30 S ribosomal protein S15	8.7
SMc00324	*pnp *probable polyribonucleotide nucleotidyltransferase	10.1
SMc00335	*rpsA *30 S ribosomal protein S1	10.2
SMc00485	*rpsD *probable 30 S ribosomal subunit protein S4	9.2/8.8
SMc00522	*rhlE1 *putative ATP-dependent RNA helicase	8.5
SMc00565	*rplI *probable 50 S ribosomal protein L9	13.4
SMc00567	*rpsR *putative 30 S ribosomal protein S18	21.9
SMc00568	*rpsF *putative 30 S ribosomal protein S6	25.9
SMc01287	*rpsM *probable 30 S ribosomal protein S13	8.5
SMc01290	*rplO *probable 50 S ribosomal protein L15	10.5
SMc01291	*rpmD *probable 50 S ribosomal protein L30	12.9
SMc01292	*rpsE *probable 30 S ribosomal protein S5	15.9
SMc01293	*rplR *probable 50 S ribosomal protein L18	24.7/12.5
SMc01294	*rplF *probable 50 S ribosomal protein L6	12.3
SMc01295	*rpsH *probable 30 S ribosomal protein S8	12.9
SMc01296	*rpsN *probable 30 S ribosomal protein S14	13.3
SMc01297	*rplE *probable 50 S ribosomal protein L5	15.4
SMc01298	*rplX *probable 50 S ribosomal protein L24	13.1
SMc01299	*rplN *probable 50 S ribosomal protein L14	16.1/13.2
SMc01300	*rpsQ *probable 30 S ribosomal protein S17	20.8/12.0
SMc01301	*rpmC *probable 50 S ribosomal protein L29	13.1
SMc01302	*rplP *probable 50 S ribosomal protein L16	12.4
SMc01303	*rpsC *probable 30 S ribosomal protein S3	17.5/10.6
SMc01304	*rplV *probable 50 S ribosomal protein L22	13.2
SMc01305	*rpsS *probable 30 S ribosomal protein S19	15.2
SMc01306	*rplB *probable 50 S ribosomal protein L2	20.5/18.1
SMc01307	*rplW *probable 50 S ribosomal protein L23	31.9
SMc01308	*rplD *probable 50 S ribosomal protein L4	24.1
SMc01309	*rplC *probable 50 S ribosomal protein L3	22.4/16.5
SMc01310	*rpsJ *probable 30 S ribosomal protein S10	25.6/19.7
SMc01312	*fusA1 *probable elongation factor G	29.6/21.0
SMc01313	*rpsG *probable 30 S ribosomal protein S7	30.4
SMc01314	*rpsL *probable 30 S ribosomal protein S12	19.5
SMc01326	*tuf *probable elongation factor TU protein	10.2/10.1
SMc02050	*tig *probable trigger factor	9.1
SMc02053	*trmFO *methylenetetrahydrofolate-tRNA-(uracil-5-)-methyltransferase	10.4
SMc02100	*tsf *probable elongation factor TS (EF-TS) protein	10.8
SMc02101	*rpsB *probable 30 S ribosomal protein S2	13.7
SMc03242	*typA *predicted membrane GTPase	14.4
SMc03859	*rpsP *probable 30 S ribosomal protein S16	8.2
		
Metabolism		
		
SMa0680	Decarboxylase (lysine, ornithine, arginine)	11.2
SMa0682	Decarboxylase (lysine, ornithine, arginine)	8.3
SMa0765	*fixN2 *cytochrome c oxidase subunit I	9.8
SMa0767	*fixQ2 *nitrogen fixation protein	11.5
SMa1179	*nosR *regulatory protein	13.8
SMa1182	*nosZ *nitrous oxide reductase	24.3
SMa1183	*nosD *nitrous oxidase accessory protein	12.4
SMa1188	*nosX *accesory protein	10.7
SMa1208	*fixS1 *nitrogen fixation protein	10.6
SMa1209	*fixI1 *ATPase	24.4
SMa1210	*fixH *nitrogen fixation protein	10.1
SMa1213	*fixP1 *di-heme c-type cytochrome	28.2
SMa1214	*fixQ1 *nitrogen fixation protein	37.2
SMa1216	*fixO1 *cytochrome C oxidase subunit	18.5
SMa1243	*azu1 *pseudoazurin	9.6
SMb21487	*cyoA *putative cytochrome o ubiquinol oxidase chain II	14.2
SMb21488	*cyoB *putative cytochrome o ubiquinol oxidase chain I	22.2
SMb21489	*cyoC *putative cytochrome o ubiquinol oxidase chain III	13.6
SMc00090	*cyoN *putative sulfate adenylate transferase cysteine biosynthesis protein	37.5
SMc00091	*cysD *putative sulfate adenylate transferase subunit 2 cysteine biosynthesis protein	21.1
SMc00092	*cysH *phosphoadenosine phosphosulfate reductase	13.4
SMc00595	*ndk *probable nucleoside diphosphate kinase	8.6
SMc00868	*atpF *probable ATP synthase B chain transmembrane protein	8.1/8.0
SMc00869	*atpF2 *probable ATP synthase subunit B' transmembrane protein	8.7
SMc00871	*atpB *probable ATP synthase A chain transmembrane protein	8.3
SMc01053	*cysG *probable siroheme synthase	13.9
SMc01169	*ald *probable alanine dehydrogenase oxidoreductase	26.2
SMc01923	*nuoJ *probable NADH dehydrogenase I chain J transmembrane protein	9.1
SMc01925	*nuoL *probable NADH dehydrogenase I chain L transmembrane protein	10.0
SMc02123	Sulfate or sulfite assimilation protein	12.6
SMc02124	*cysI *putative sulfite reductase	20.2
SMc02479	*mdh *probable malate dehydrogenase	9.9
SMc02480	*sucC *probable succinyl-CoA synthetase beta chain	9.4
SMc02481	*sucD *probable succinyl-CoA synthetase alpha chain	9.3
SMc02499	*atpA *probable ATP synthase subunit alpha	8.2
SMc02500	*atpG *probable ATP synthase gamma chain	16.2/11.1
SMc02502	*atpC *probable ATP synthase epsilon chain	9.8
SMc03858	*pheA *putative chorismate mutase	8.4
		
Transport		
		
SMa1185	*nosY *permease	8.5
SMb20346	Putative efflux transmembrane protein	8.3
SMc00873	*kup1 *probable KUP system potassium uptake transmembrane protein	11.4
SMc02509	*sitA *manganese ABC transporter periplasmic substrate binding protein	9.4
SMc03157	*metQ *probable D-methionine -binding lipoprotein MetQ	8.7/14.9
SMc03158	*metI *probable D-methionine transport system permease protein MetI	12.3
SMc03167	MFS-type transport protein	41.1
SMc03168	Multidrug resistance efflux system	41.5
		
Stress related		
		
SMa0744	*groEL2 *chaperonin	18.3/13.7
SMa0745	*groES2 *chaperonin	19.3
SMa1126	Putative protease, transmembrane protein	16.4
SMb21549	*thtR *putative exported sulfurtransferase, Rhodanese protein	29.3
SMb21562	Hypothetical membrane-anchored protein	69.6
SMc00913	*groEL1 *60 KD chaperonin A	17.5
SMc02365	*degP1 *probable serine protease	20.4/18.5
		
Motility		
		
SMc03014	*fliF *flagellar M-ring transmembrane protein	8.3
SMc03022	*motA *chemotaxis (motility protein A) transmembrane	16.2
SMc03024	*flgF *lagellar basal-body rod protein	15.6
SMc03027	*flgB *flagellar basal-body rod protein	9.3
SMc03028	*flgC *flagellar basal-body rod protein	12.9
SMc03030	*flgG *flagellar basal-body rod protein	11.0
SMc03047	*flgE *flagellar hook protein	8.1
SMc03054	*flhA *probable flagellar biosynthesis transmembrane protein	9.7

^1 ^Some *S. meliloti *genes have more than one probe set represented on the array. In these cases, more than one fold change value is shown.

**Table 2:** Genes with more than 5-fold decreased expression in the *tolC *mutant strain.

Gene identifier	Annotation or description	Fold change^1 ^(*tolC *vs. wild-type)
Transcription and signal transduction		
		
SMa0402	Transcriptional regulator, GntR family	-8.4
SMb21115	Putative response regulator	-20.2
SMc01042	*ntrB *nitrogen assimilation regulatory protein	-8.0
SMc01043	*ntrC *nitrogen assimilation regulatory protein	-6.9
SMc01504	Receiver domain	-7.2
SMc01819	Transcription regulator TetR family	-10.0
SMc03806	*glnK *probable nitrogen regulatory protein PII 2	-9.1
		
Metabolism		
		
SMa0387	*hisC3 *histidinol-phosphate aminotransferase	-11.4
SMa0398	*hisD2 *histidinol dehydrogenase	-10.6
SMa1683	Arylsulfatase	-5.0
SMb20984	*nirB *nitrite reductase NAD(P)H	-22.7
SMb20985	*nirD *nitrite reductase NAD(P)H	-26.6
SMb20986	*narB *putative nitrate reductase, large subunit	-14.1
SMb20987	Putative uroporphiryn-III C-methyltransferase	-7.6
SMb21094	*argH2 *argininosuccinate lyase	-20.7
SMb21163	*hutU *urocanate hydratase (urocanase)	-10.3
SMb21164	*hutG *Putative formiminoglutamase	-11.5
SMb21165	*hutH *Putative histidine ammonia-lyase histidase	-7.7
SMc01041	*dusB *tRNA-dihydrouridine synthase B	-9.5
SMc01814	Probable glutamate synthase small chain	-12.5
SMc01820	Putative N-carbamyl-L-amino acid amidohydrolase	-12.7
SMc01967	*speB2 *putative agmatinase	-18.7
SMc03208	*hmgA *homogentisate 1,2-dioxygenase	-5.5
SMc04026	*gltD *probable glutamate synthase small chain	-9.2
SMc04028	*gltB *probable glutamate synthase NADPH large chain	-11.7
SMc04153	Putative aminomethyltransferase	-8.7
SMc04323	Probable aminotransferase	-7.8
		
Transport		
		
SMa0391	ABC transporter, ATP-binding protein	-15.6
SMa0392	ABC transporter, periplasmic solute-binding protein	-8.3/-23.5
SMa0394	ABC transporter, permease	-10.5
SMa0396	ABC transporter, permease	-10.1
SMa0581	*nrtC *nitrate transporter, ATP binding protein	-24.8
SMa0583	*nrtB *nitrate transporter, permease	-33.0
SMa0585	*nrtA *nitrate ABC transporter, periplasmic nitrate binding protein	-34.8
SMb20436	Probable nitrate transporter	-62.2/-63.5
SMb20602	ABC transporter, ATP-binding protein	-12.0
SMb20603	ABC transporter, permease	-15.7
SMb20604	ABC transporter, permease	-25.0
SMb20605	ABC transporter, periplasmic solute-binding protein	-22.4
SMb21095	ABC transporter, permease	-10.3
SMb21096	ABC transporter, permease	-10.7
SMb21097	ABC transporter periplasmic solute-binding protein	-17.5
SMb21114	Putative nitrate transport protein	-10.3
SMb21707	ABC transporter, ATP-binding protein	-14.4
SMc01597	Putative amino acid permease	-8.1
SMc01963	Spermidine/putrescine transport system permease	-5.2
SMc01964	Putative spermidine/putrescine transport system permease ABC transporter	-5.8
SMc01965	Spermidine/putrescine ABC transporter ATP-binding subunit	-7.4
SMc01966	Putative spermidine/putrescine-binding periplasmic ABC transporter	-12.4
SMc03807	*amtB *probable ammonium transporter	-8.1
SMc04147	Putative amino acid permease	-10.7

^1 ^Some *S. meliloti *genes have more than one probe set represented on the array. In these cases, more than one fold change value is shown.

To confirm data obtained by microarray analysis, we examined expression of ten representative genes of the functional categories of signal transduction, secondary metabolism, carbohydrate, amino acids and inorganic ion transport and metabolism, and protein turnover by real-time RT-PCR (Table 3). Gene *glnA *was chosen because its expression was moderately decreased in the *tolC *mutant background; genes *smoG *and *rem *had a moderately increased expression; genes *gltB*, *argH2 *and *nrtA *showed greatly decreased expression; SMc03167, *cysN *and *degP1 *gene expression was highly increased. *wgeA *was included as a control, since microarray analysis showed that its expression was not significantly altered. Results obtained by quantitative real-time PCR were in agreement with microarray data (Table 3). Below we discuss a selected subgroup of differentially expressed genes.

**Table 3:** Quantitative real-time RT-PCR analysis performed in *S. meliloti *1021 wild-type and *tolC *mutant cells.

Gene	Microarray Fold change	Real-time PCR Fold change ± SD
*rem*	4.2	4.6 ± 1.4
*wgeA*	1.0	1.0 ± 0.2
*SMoG*	3.9	2.9 ± 0.8
SMc03167	41.1	58.2 ± 7.2
*glnA*	-3.8	-2.3 ± 0.1
*gltB*	-11.7	-11.0 ± 1.6
*argH2*	-20.7	-4.5 ± 1.7
*nrtA*	-34.8	-19.4 ± 2.4
*cysN*	37.5	19.4 ± 0.9
*degP*	18.5	31.2 ± 1.1

### Oxidative stress is induced in the *tolC *mutant

Bacteria have developed several different strategies to cope with fatal stress conditions. One is mediated by alternative sigma-32 factor (RpoH) that, besides high temperature, is activated by conditions that destabilize folded proteins or make correct nascent protein folding more difficult [17]. *S. meliloti*, as well as some other rhizobia, has two *rpoH *genes named *rpoH1 *and *rpoH2*. Comparison of the transcriptome of *S. meliloti tolC *mutant with the one of the wild-type strain, revealed a 3.4-fold increase in *rpoH1 *gene expression. *rpoH2 *gene expression was not altered. Both RpoH1 and RpoH2 sigma factors were studied in *Rhizobium etli*, with the results suggesting that they operate under different stress conditions: RpoH1 in heat-shock and oxidative stress, and RpoH2 in osmotic tolerance [18]. The increase of *rpoH1 *gene expression in the *tolC *mutant is probably a consequence of the stress conditions caused by accumulation of intracellular proteins or of other unknown cell metabolites and probably due to a higher metabolic rate. Similarly to the increase of *rpoH1 *gene expression, significantly increased expression of many genes that in other organisms are known to belong to the *rpoH *regulon was observed. These encompass genes encoding chaperones DnaJ, DnaK, ClpB, GroESL1, GroESL2, GroESL3, GroEL5, GrpE, HslO, HtpG, and IbpA, involved in the folding of newly synthesized proteins or in refolding of denatured proteins to maintain homeostasis. Under free-living conditions, *S. meliloti *RpoH1 seems to control the expression of *groESL5 *but not other chaperone encoding genes [19]. Here, beside *groESL5*, more than 17-fold increase of *groESL1 *and *groESL2 *operons expression was detected (Table 1), suggesting they may be regulated by another transcription factor or by RpoH1 in a stress condition dissimilar from the heat-shock tested by Bittner and Oke [19]. Several genes encoding proteases and protein modification enzymes such as ClpP1, ClpP2, ClpX, Lon, HslUV, HflCKX, FtsH, HtpX and Dcp also showed significantly increased expression in the *tolC *mutant. In addition to protecting proteins from destruction or degradation of the denatured ones the *rpoH *regulon also protects other macromolecules like DNA and RNA [17]. In the *tolC *mutant we observed increased expression of the gene encoding Mfd which recruits the DNA repair machinery to lesions, as well as genes such as *mutM*, *recJ*, *topA *and *xerD *encoding products known to maintain genomic integrity [20].

Reinforcing the idea of the *tolC *mutant strain being under stress, the expression of many transcripts encoding enzymes involved in detoxification and protection against oxidative stress was increased. Examples include *gst1*, *gst4*, *gst7 *and *gst11*, all of which encode glutathione S-transferases. Glutathione transferase proteins catalyze nucleophilic attack by the tripeptide glutathione (GSH) on a wide range of hydrophobic toxic compounds. They are also capable of non-catalytically binding a large number of endogenous compounds, playing an active role in protection against oxidative stress and detoxification of harmful xenobiotics [21]. Other genes with increased expression were *katA *(3.7-fold) encoding a catalase, *sodB *(2.4-fold) encoding a superoxide dismutase, *cpo *(2.5-fold) encoding a chloride peroxidase, and *gor *(1.8-fold) encoding a glutathione reductase. Gene *thtR *showed the greatest expression in this functional class with a 29.3-fold increase (Table 1). *thtR *encodes a protein homologous to tiosulphate sulfurtransferases of the Rhodanese family, which catalyze the transfer of the sulphate atom of thiosulphate to cyanide, to form sulphite and thyocianate. Several studies indicate that these proteins may function as antioxidants capable of scavenging oxidative species that would otherwise lead to inactivation of enzymes such as those containing Fe-S clusters [22].

**Figure 4:**
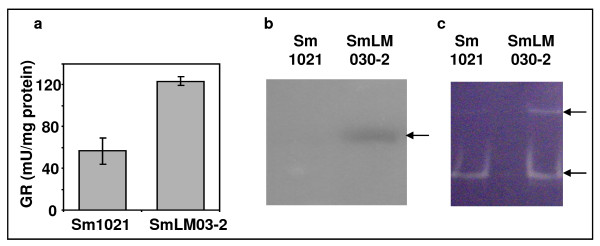
**Activity of enzymes combating oxidative stress**. Enzymatic activities of (a) glutathione reductase as measured spectrophotometrically at 412 nm; (b) catalase and (c) superoxidase dismutase in native gel after staining. Total protein extracts were obtained after growing the wild-type strain Sm1021 and the *tolC *mutant strain SmLM030-2 for 20 hours in GMS medium. 20 μg of crude extract were loaded in each lane. Arrows indicate the position of bands obtained.

To confirm microarray data and demonstrate that the *tolC *mutant is under oxidative stress, enzymatic activities of catalase, superoxide dismutase and glutathione reductase were determined in cells grown in GMS medium for 20 hours (Fig. 4). Results showed that the specific activity of glutathione reductase in the total protein extract of the *tolC *mutant was twice that of the wild-type strain (Fig. 4a). In-gel activity staining was used to visualize catalase activity. Despite increased expression of the *katA *gene and decreased *katB *expression compared to the wild-type strain, increased catalase activity was detected in the *tolC *mutant (Fig. 4b). SOD activity was also higher in the *tolC *mutant (Fig. 4c). The active SodB protein is a dimer [23] and corresponds probably to the lower band, while the upper band must be a multimeric form. Taken together, the increase of the three enzyme activities analyzed provides evidence that under conditions where the outer membrane protein TolC is non functional, cells suffer internal oxidative stress. Sikora et al. [24] recently demonstrated that mutants of *Vibrio cholerae *with compromised membrane phenotypes showed higher concentrations of radical oxygen species (ROS), induction of oxidative stress and changes in iron physiology. It is possible that the observed oxidative stress response of the *S. meliloti tolC *mutant is mainly caused by a compromised cell envelope, although a higher metabolic rate and accumulation of proteins and metabolites which can not be secreted may also contribute to stress.

In both *Vibrio cholerae *and *E. coli*, cell envelope perturbations resulted in induction of the extracytoplasmic stress factor RpoE, which directs transcription of genes involved in envelope maintenance [25]. We observed decreased expression of *rpoE2*, as well SMc01505 which is co-transcribed with *rpoE2 *and encoding an anti-sigma factor, suggesting that the lack of a functional TolC protein does not trigger RpoE-dependent stress response. Instead, by comparing the expression profile of the *S. meliloti tolC *mutant with that of the wild-type strain, we observed 69-, 27-, and 14-fold increased expression in genes SMb21562, SMb21561, and SMb21560, respectively (Table 1). Amino acid sequence of SMb21562 shows identity with the periplasmic protein CpxP from several Enterobacteria, displaying two characteristic LTxxQ motifs (data not shown). SMb21560 encodes a putative sensor histidine kinase homologous to CpxA. SMb21561 encodes a putative response regulator homologous to CpxR. The Cpx two-component regulator is a well characterized system to sense misfolded proteins in the periplasm and other perturbations in the cell envelope [26,27]. In Cpx signaling, unfolded proteins are recognized by CpxP, a periplasmically located inhibitor of the signaling sensor kinase CpxA, preventing CpxA to autophosphorylate. Nonphosphorylated CpxA is then unable to phosphorylate the cytoplasmic response regulator CpxR. The Cpx regulon of *E. coli *strain MC4100 contains at least 50 genes, some directly involved in maintenance of cell envelope proteins. These include periplasmic serine endoprotease DegP, disulfide oxidoreductase Dsb, periplasmic peptidyl-prolyl isomerase PpiA, phosphatidyl serine decarboxylase Psd, YccA, a modulator of FtsH proteolysis, periplasmic protein CpxP, and the two-component regulator CpxAR [28]. In addition, outer membrane protein OmpF, shikimate kinase AroK, and sigma-E regulator RpoE-RseABC are under negative control by Cpx [28]. Targets of the CpxR homologue in *S. meliloti *are completely unknown, but expression of genes encoding DegP proteases (*degP1P3P4*) and peptidyl-prolyl isomerase Ppi (*ppiABD*) were significantly increased in *tolC *mutant. A search for the *E. coli *CpxR binding site GTAAAN_5_GTAAA consensus sequence in the upstream coding regions of *S. meliloti *using the RSA-tools web interface revealed that this sequence matched the putative promoter region upstream of the predicted operon SMb21562/SMb21561/SMb21560. In a recent study, the CpxR protein from *Yersinia enterocolitica *was shown to negatively affect transcription of gene *rpoE*, coding for the extracytoplasmic sigma-E factor [29]. We also observed decreased expression of *rpoE2 *and *rpoE8 *genes. Our data suggest that in the absence of a functional TolC, cells trigger a Cpx instead of an RpoE-mediated response. A very different situation was observed in wild-type *S. meliloti *cells grown under different stress conditions such as osmotic shock [30,31], high metal ion concentration [32], acidic pH [33], heat shock and entry in stationary phase [34] where an *rpoE2*-mediated response was induced. This seems to indicate that the external stress imposed on the cells triggers a well defined extracytoplasmic response. When perturbations to the cell envelope, such as the absence of a functional outer membrane protein occur, cells seem to activate a distinct stress response pathway.

### Genes involved in transcription and translation

It is possible that under the cytoplasmic and extracytoplasmic stress conditions experienced by the *tolC *mutant, many proteins and cofactors become inactive and need to be synthesized *de novo *or protected from denaturation. It is then not surprising that many genes encoding proteins involved in transcription and translation were found to have significantly increased expression in the *tolC *mutant strain. This was the case for genes encoding all RNA polymerase subunits (*rpoABCZ*), genes *nusA *and *nusG *involved in transcriptional pausing, termination, and antitermination, and the gene encoding transcription termination factor Rho. RNA degradation is mediated by the RNA degradosome, a multiprotein complex involving RNase E, polynucleotide phosphorylase (PNPase), helicase RhlB, and enolase [35]. In *S. meliloti*, those components are encoded by the genes *rne*, *pnp*, *deaD*, and *eno*, respectively, all of them showing increased expression in *tolC *mutant suggesting that, besides increased expression of genes encoding products involved in transcription, the mutant also increases expression of genes encoding products participating in RNA degradation.

Of the 105 genes differentially expressed and involved in translation and ribosome biogenesis only three had a decreased expression in the *tolC *mutant. Genes with increased expression encode 53 ribosomal proteins, along with initiation (*infA*, *infB *and *infC*), elongation (*tsf*, *fusA1*, *efp*) and release factors (*prfA*, *prfB *and *prfC*) (Table 1). In the *tolC *mutant we observed an increased expression of *rbfA *and *rimM*, coding for a ribosome binding factor and an rRNA-processing protein, respectively. Both gene products are essential for efficient processing of 16 S rRNA in *E. coli *[36]. The *rrmJ *gene encoding a ribosomal RNA large subunit methyltransferase and genes *ksgA *and *hemK1 *encoding two methylases involved in quality control by the small subunit of the ribosome [37] and methylation of release factors [38], respectively, also showed increased expression in the *tolC *mutant. Concerning amino acyl-tRNA modification we observed increased expression of the *trmFO *gene encoding a folate-dependent tRNA methyltransferase in the *tolC *mutant (Table 1). Maturation of tRNA precursors into functional tRNA molecules requires trimming of the primary transcript at both the 5'and 3'ends and is catalyzed by RNase P and RNase PH. Expression of genes encoding RNase P (*rnpA*) and RNase PH (*rph*), and genes encoding Rnase D (*rnd1 *and *rnd2*) which contribute to the 3'maturation of several stable RNAs also displayed increased expression levels in the *tolC *mutant. In contrast to *S. meliloti *cells exposed to osmotic stress which showed decreased expression of genes involved in protein metabolism [30,31], *tolC *mutant cells showed increased expression of these genes. As mentioned previously, a plausible explanation would be the need for new proteins to replace denatured ones due to oxidative stress conditions and the higher levels of metabolic enzymes needed for the cell to produce energy.

### Genes involved in energy and central intermediary metabolism

**Figure 5:**
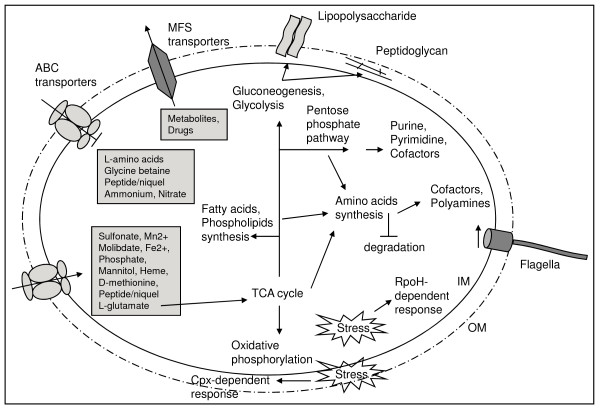
**Altered pathways and phenotypes on the dependence of *tolC *mutation in *S. meliloti *as depicted from the expression data**. Arrows represent processes/pathways whose genes displayed increased expression and blocked arrows decreased expression in absence of a functional TolC protein. IM, inner membrane; OM, outer membrane.

We found increased expression of multiple genes involved in central metabolism and energy production in the *tolC *mutant (Fig. 5), suggesting a higher metabolic rate in response to *tolC *gene mutation. For instance, genes encoding 11 out of 12 of the enzymes involved in the tricarboxylic acid cycle (TCA) (*acnA*, *icd*, *sucABCD*, *lpdA1A2*, *sdhABCD*, *fumC *and *mdh*), along with genes encoding many enzymes of the Calvin-Benson-Bassham reductive pentose phosphate pathway (*rbcL*, *pgk*, *fbaB*, *cbbF*, *tkt2*, *cbbT*, *rpiA *and *rpe*) and most genes encoding enzymes for the glycolysis and gluconeogenesis pathways (*cbbF*, *fbaB*, *tpiA1*, *gap*, *pgk*, *eno*, *pdhA*) had significantly increased expression (Fig. 5). Alongside the increased expression of the genes encoding TCA enzymes, all genes encoding different protein complexes in the respiratory chain had also an increased expression. Genes include *nuoA1B1C1D1E1F1G1HIJK1LMN *and *ndh *forming NADH dehydrogenase (complex I); *sdhABCD *from fumarate reductase (complex II); *fbcBCF *from cytochrome *c *reductase (complex III); *ctaCDEG *and SMc01800 from cytochrome *c *oxidase (complex IV); and *atpCDGABEF2FH *from ATP synthase (complex V) (Table 1). *cbb3*-type cytochromes encoded by genes *fixN2O2Q2P2 *and *fixP1Q1O1N1*, and a low O_2 _affinity oxidase encoded by the *cyoABC *operon also showed increased expression in the *tolC *mutant (Table 1). Increased expression of all genes listed suggests that the *tolC *mutant strain may be metabolically more active. Nevertheless, the *tolC *mutant forms less biomass as seen in Fig. 1. This apparent contradiction can be explained if stress inflicted by cell envelope perturbations due to the absence of functional TolC protein results in a higher ATP turnover. Additional ATP would be consumed to maintain cell homeostasis and not to form biomass. It is also a formal possibility that perturbations to the cell envelope may reduce the proton electrochemical gradient, negatively affecting ATP synthesis and therefore creating the need to increase the expression of genes related to energy metabolism.

Due to the general increase in expression of genes involved in translation, it was not surprising to see increased expression of genes encoding proteins involved in amino acid and cofactors biosynthesis in the *tolC *mutant (Fig. 5). Regarding cofactor biosynthesis we observed an increased expression in the *tolC *mutant of genes encoding enzymes for thiamine (*thiE2*, *nifS*), folate (*folBCE*, *exsC*), riboflavin (*ribADEH*), nicotinate and nicotimanide metabolism (*nadABC*, *pntBAaAb*), as well as genes *panBC*, *coaAD*, *ilvCD2HI *and *acpS *encoding enzymes required for pantothenate and CoA biosynthesis. Regarding amino acid metabolism by the *tolC *mutant there was an increased expression of genes encoding enzymes involved in the biosynthesis of the majority of them. These included *serAB*, *glyA*, SMc04029, *lysC*, *asd*, *thrABC1*, *metAZHK*, *sda *and *metK1K2 *for L-serine, L-glycine, L-threonine, L-methionine and L-cysteine biosynthesis; the genes *leuBD*, *ilvCD2E1HI *and *pdhAaAb *encoding enzymes for the synthesis of L-isoleucine, L-valine and L-leucine; the gene *ald *(Table 1) encoding an alanine dehydrogenase oxidoreductase synthesizing L-alanine from ammonia and pyruvate; the genes *aroABCEFKQ*, *pheAAa*, *trpABDEF*, *tatA*, *tyrC*, and *aatAB *encoding enzymes for biosynthesis of aromatic amino acids L-phenylalanine, L-tyrosine and L-tryptophan and genes *hisABC1C2DEFGHIZ *for the biosynthesis of L-histidine. Contrastingly, *hutGHH2U *genes involved in L-histidine degradation had more than 7-fold decreased expression (Table 2). Genes encoding enzymes for the biosynthesis of amino acid lysine (*lysAC*, *asd*, *dapAA3BDF*) had increased expression and those for degradation reduced expression levels (SMb21181, *fadAB*, *phbA*). Genes encoding urea cycle enzymes are *argBDEJ*, *arcA1A2B *and *argF1GH1H2*. With the exception of *arcA2 *and *argH2 *which encode a second copy of arginine deiminase and argininosuccinate lyase, respectively, all of them showed increased expression levels in *tolC *mutant. Commencing from ornithine or arginine it is possible to obtain the polyamines putrescine and agmatine. SMa0680 and SMa0682 (Table 1) encoding putative amino acid decarboxylases and the putative agmatinase encoded by gene *speB *were induced in the *tolC *mutant (Table 1). Polyamines are polycationic molecules that have important functions in cell physiology, contributing to stabilization of nucleic acids, production and function of outer membrane porins or are free radical scavengers when cells are exposed to oxidative stress [39]. Polyamine biosynthesis can therefore be another strategy used by the *tolC *mutant when under stress conditions.

In accordance with the hypothetical higher availability of metabolic intermediary compounds in the *tolC *mutant, *fabBFGHIZ *and *accABCD *encoding the enzymes for fatty acid biosynthesis; *gpsA*, *plsC*, *cdsA*, *pgsA*, *pssA*, and *pcs *involved in phospholipid biosynthesis; *pyrBCDEFGH*, *cmk *and *ndk *involved in pyrimidine nucleotides biosynthesis, and *purBCDEFHKLMNQS *and *guaAB *for purine nucleotides all had an increased expression in this mutant.

We observed 7-fold decreased expression of the genes *ntrBC *encoding the two-component regulatory system NtrBC in the *tolC *mutant, and decreased expression of NtrC-dependent genes encoding glutamine synthetases (*glnII*, *glnA*), regulatory P_II _proteins (*glnB*, *glnK*), and the AmtB transporter (*amtB*) (Table 2). A possible explanation could be intracellular differences in the C/N ratio between the two strains studied. Patriarca et al. [40] showed in *Rhizobium etli *cells grown in the presence of glutamine as single carbon and nitrogen source that the intracellular α-ketoglutarate/glutamine ratio influence NtrC activity.

### Genes involved in transport

In keeping with the hypothesis of a higher metabolic rate in the *tolC *mutant, many genes related to nutrient uptake and assimilation showed increased expression in this strain including *cysA2P2*, SMb21132 and SMb21133 putatively involved in sulfate transport and *cysDHIK1K2N *encoding products involved in sulfate assimilation (Table 1). SMc04049 encoding a putative sulfite oxidase that converts sulfite back to sulfate had a decreased expression, possibly ensuring that in the *tolC *mutant sulfur flows in the direction of assimilation only. Other genes with increased expression in the *tolC *mutant were genes *modABC *encoding a putative molybdate ABC transporter; genes *sitABCD *encoding a manganese transporter; the genes *pstABS *and *phoCDT *encoding putative phosphate transporters; genes associated to biotin uptake (*bioMN*); *kup1 *and *kup2 *and *corA2 *putatively involved in K^+ ^and Mg^2+^/Co^2+ ^uptake, respectively; many genes related to iron (SMb21429, SMb21430, SMb21431 and SMb21432) and Fe^3+^-siderophore uptake (SMa1741, SMa1742, SMa1745, SMa1746 and *exbBD*); and genes encoding heme compound transporters (*hmuTUV *and *ccmBC*) (Fig. 5). An increase in the *tolC *mutant of the expression of *smoEFGK *genes involved in the uptake of mannitol, a carbon source provided in our experiments, was also observed. As regards amino acid transport, the genes *metINQ*, encoding an ABC transporter putatively involved in the transport of D-methionine (Table 1) also showed increased expression in the *tolC *mutant. We observed a strong decrease in the expression of genes involved nitrate, ammonium and amino acids transport in the *tolC *mutant (Fig. 5). For example, nitrate transporters encoded by *nrtABC*, SMb21114 and SMb20436 showed in excess of 10-fold decreased expression while the ammonium transporter encoded by the *amtB *gene showed 8-fold decreased expression. Genes associated with general amino acid transport (*aapJMPQ*) and branched-chain amino acids transport (SMb20602, SMb20603, SMb20604, SMb20605 and SMb21707) also displayed more than 12-fold decreased expression (Table 2). Genes encoding another ABC-type transporter putatively involved in the transport of spermidine/putrescine (SMc01963, SMc01964, SMc01965 and SMc01966) had 5-fold decreases expression while two putative ABC-type transporter systems of unknown function (SMb21095, SMb21096, SMb21097 and SMa0391, SMa0392, SMa0394 and SMa0396) had 10-fold decreased expression in the *tolC *mutant (Table 2). The decreased expression of genes involved in nitrogen-rich compound transport is probably an effect of decreased NtrC expression and is maybe a way to prevent a futile export and import cycle of these compounds.

**Figure 6:**
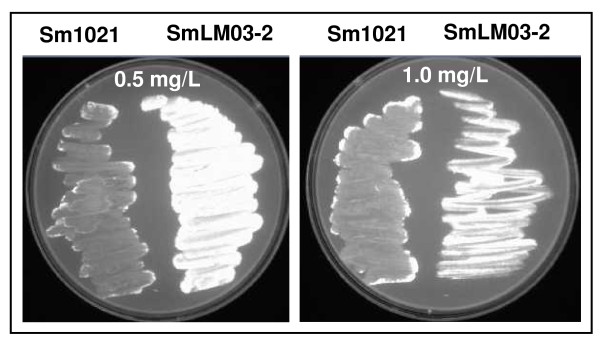
**Evaluation of efflux activity**. *S. meliloti *1021 (left side of the plate) and the *tolC *mutant SmLM03-2 (right side of the plate) were swabbed in GMS plates containing 0.5 and 1.0 mg/ml of ethidium bromide, following incubation at 30°C for 48 hours and detection under UV light.

The *tolC *mutant exhibits an envelope defect, typified by its sensitivity to membrane-disrupting agents such as sodium dodecyl sulfate and deoxycholate [15]. When wild-type *S. meliloti *and *tolC *mutant strains were grown in solid GMS media supplemented with ethidium bromide it was observed that *tolC *mutant cells were fluorescent whilst wild-type cells were not (Fig. 6). This fluorescence results from the accumulation of ethidium bromide inside the *tolC *mutant cells, probably caused by their inability to pump this toxic compound out. This result suggests impairment of transport functions, most probably caused by the absence of the functional outer membrane protein TolC. Even when the *tolC *mutant is grown in GMS medium in the absence of toxic extracellular compounds, it is possible that unknown metabolites can not be secreted and accumulate in the cells, causing toxicity. To relieve that negative effect, cells would increase the expression of genes encoding certain transporters. This could explain the 5- and 41-fold increase in the expression of genes SMb20345/SMb20346 and SMc03167/SMc03168, respectively, which encode two putative transporters from the major facilitator superfamily, and the 1.4-fold increase in expression of truncated *tolC *gene. Similar reasoning was suggested by Rosner and Martin [8] in the case of *E. coli *TolC protein (together with other transport proteins) regarding the secretion of unknown cellular metabolites.

### Genes with a role in cell division and envelope biogenesis

In our data set the *dnaA *gene encoding a protein controlling chromosome replication initiation had increased expression in the *tolC *mutant. In *C. crescentus *DnaA controls expression of approximately 40 genes involved in, amongst others, DNA replication, recombination and repair, cell division and cell envelope biogenesis [41]. Expression profiles of genes putatively regulated by DnaA and involved in DNA replication, such as genes encoding subunits of DNA polymerase III, DnaB helicase, single strand DNA binding protein Ssb, RNase H and DNA polymerase I; DNA recombination (*recJ*, *recN*, *recR*, *ruvC*); and DNA repair (*mutS*, *mutT*, *mutM*, *uvrA*, *uvrB*, *uvrC*, *uvrD*, *mfd*) showed an increased expression in the *tolC *mutant. *ctrA*, encoding a member of the two-component signal transduction family involved in silencing replication initiation showed significantly decreased expression in the *tolC *mutant. We also observed increased expression of two genes encoding Maf-like proteins (SMc02311 and SMc02792). Expression of a *maf*-like gene was also increased in *S. meliloti *after NaCl osmotic shock [30]. In *Bacillus subtilis*, overexpression of *maf *results in inhibition of septation, leading to extensive filamentation [42]. To evaluate whether the *tolC *mutant cells showed morphological changes, microscopic analysis after staining of cells with crystal violet was performed at 17, 24 and 48 hours of growth. No significant differences were seen concerning size or shape of the two cell types at any time point (data not shown). Increased expression of *maf*-like genes could suggest inhibition of cell division in the *tolC *mutant in accordance to the lower optical density observed in the growth curve (Fig. 1). On the other hand, we observed an increased expression of genes involved in chromosomal replication. This apparent contradiction could be explained if, at the time of cell collection and total RNA extraction, the wild-type cells were growing less quickly than the *tolC *mutant cells, due to entry into stationary phase.

Expression profiling of genes encoding enzymes needed for lipopolysaccharide synthesis (LPS), such as the *lpxABDKL *genes involved in lipid A biosynthesis, and *lpsBCDES*, *kdsA, kdsB *and *kdtA *encoding enzymes for the biosynthesis of the LPS core showed a significantly increased expression in the *tolC *mutant. Regarding peptidoglycan biosynthesis we observed increased expression in the *tolC *mutant of *murACEFG *genes, the undecaprenyl pyrophosphate phosphatase *uppP *and synthase *uppS*. Three penicillin-binding protein encoding genes (*mrcA1*, *mrcB *and *dac*) and several putative lytic murein transglycosylases (SMc04411, *mltB1*, *mltB2*, SMc02785) also displayed increased expression. Multiple genes involved in capsular polysaccharide biosynthesis, including the *rkpUAGHIJ *and *kpsF3 *genes located on the chromosome and previously demonstrated to be involved in symbiotic capsular polysaccharide biosynthesis [43] and four genes from pSymB (*rkpT2*, *rkpZ2*, SMb20824, SMb20825) possibly also related to capsular polysaccharide biosynthesis showed increased expression in the *tolC *mutant. Increased expression of genes encoding products for synthesis of LPS, peptidoglycan and capsular polysaccharide may be linked to extracytoplasmic stress response activation to neutralize the compromised cell envelope.

We had previously shown that the *tolC *mutant strain is unable to produce succinoglycan in GMS medium [15]. Whether that was related to differences at transcriptional level or to post-transcriptional regulation was unknown. *exo *gene expression is positively regulated by the regulator MucR [44] and negatively by ExoR [45]. Here *mucR *gene expression was significantly increased whilst *exoR *was decreased when the transcription profile of the *tolC *mutant was compared to that of the wild-type strain. This could suggest increased expression of the *exo *genes directing succinoglycan biosynthesis in the *tolC *mutant. However, none of the *exo *genes had significant changes at the level of expression, with the exception of *exoN *encoding UDP-glucose pyrophosphorylase, which showed decreased expression, and the gene *exoU *encoding a glycosyltransferase the expression of which was increased. Apparently the absence of succinoglycan from the *tolC *mutant is not caused by differences at the transcription level. It appears more probable that, due to cell envelope perturbations, the exopolysaccharide polymerization and secretion multienzyme complex does not assemble properly or is inactive and therefore no exopolysaccharide is secreted. Also no difference was observed in the expression of genes involved in galactoglucan biosynthesis, with the exception of the transcriptional activator encoding gene *wggR *[46] that showed a decreased expression. Our results contrast with those obtained for *S. meliloti *cells stressed with salt or acid pH, where genes encoding proteins for exopolysaccharide biosynthesis showed increased expression [30,33].

### Genes involved in motility and chemotaxis

**Figure 7:**
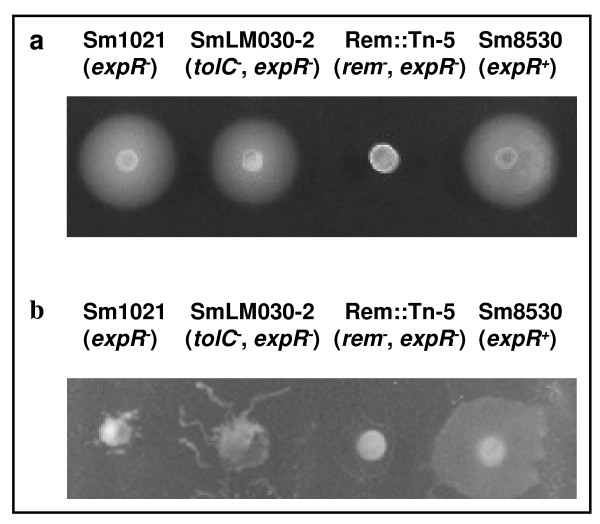
**Swimming (a) and swarming (b) tests**. Swimming and swarming plates containing 0.3% and 0.6% purified agar, respectively, were spotted with 5 μl of late exponential *S. meliloti *cultures grown overnight in GMS medium. The photographs were taken after 1 day of incubation for swarming and 3 days for swimming at 30°C.

Analysis of gene expression levels in the flagellar regulon indicated an approximately 2-fold increased expression in the *tolC *mutant of *cheABDRW1W2XY1Y2 *and *mcpU *genes, whose products are involved in chemotaxis. Most of the *fli*, *flh*, *mot*, *flg *and *fla *genes encoding proteins for the basal body, L and P rings, hook filament, motor switch and flagellum also displayed increased expression in the *tolC *mutant (Table 1). To test whether differences in the expression of motility genes leads to a phenotype in GMS semi-solid media, swimming and swarming tests were performed using the two strains. Two further strains used in this test were an *S. meliloti *transposon mutant (Rem::Tn-5) as a negative control, unable to swim or swarm due to inactivation of the transcription regulator Rem, and strain Sm8530 (*expR*^+^) as positive control for the two types of motility. Representative images are shown in Fig. 7. Despite increased expression in the *tolC *mutant of several *fli*, *flh*, *mot*, *flg *and *fla *genes, we observed no difference between swimming motility of the *tolC *mutant and the wild-type strains, with both strains being able to swim (Fig. 7a). Regarding swarming motility, we found that after 24 hours of incubation the *tolC *mutant displayed a higher surface motility than the wild-type strain (Fig. 7b), consistent with our gene expression data. The swarming behavior of wild-type and *tolC *mutant strains was markedly different from the *expR*^+ ^positive control strain Sm8530, which spread over the agar uniformly in all directions whilst the two first strains had a growth branching out from the center of the colony (Fig. 7b). *S. meliloti *cells stressed with acidic pH or increased osmotic pressure due to salt or sucrose showed decreased expression of genes involved in chemotaxis and motility, consistent with the cell needing to conserve energy [30,31,33]. Why the *tolC *mutant has increased swarming motility is not known.

## Conclusions

The transcriptomic data presented here indicate that the absence of functional TolC protein in *S. meliloti *compromises cell homeostasis as reflected by the concomitant increase in expression levels of many genes putatively involved in cytoplasmic and extracytoplasmic stress responses. Intracellular stress can possibly be caused by accumulation of proteins and metabolites that can not be secreted combined with oxidative stress. To ameliorate adverse effects, a RpoH-dependent response is triggered with an increase in the expression of many genes encoding products protecting macromolecules like DNA, RNA and proteins and helping their turnover. Perturbations in the cell envelope caused by a potential accumulation of proteins such as the truncated TolC in the periplasm may have triggered a Cpx-dependent stress response with a set of genes encoding periplasmic proteases, chaperones and protein modifying enzymes having increased expression. Increased protein synthesis causes increased expression of the genes responsible for transcription, translation and energy producing pathways. The hypothetical higher metabolic demand was mirrored by increased expression of genes encoding nutrient uptake transport systems. Further support for our observations that cell envelope perturbation leads to extracytoplasmic and to oxidative stress comes from recent studies in *Vibrio cholerae *type II secretion mutants [24]. Sikora et al. [24] showed that type II secretion mutants having compromised membrane integrity, suffer from internal oxidative stress and increased levels of intracellular ferrous iron. Nevertheless, they observed the induction of an RpoE-mediated stress response, whilst we observed a Cpx-mediated stress response, emphasising the differences between the two types of mutations/organisms. Responses to stress caused by *S. meliloti *lack of functional TolC are distinct from other stress conditions such as osmotic shock and acid pH [30,33]. In the latter two there is general shut-down of the expression of genes involved in central metabolism, protein metabolism, iron uptake and chemotaxis. In contrast, the *tolC *mutant shows an increased expression of genes involved in all of these pathways. One possible explanation could be the higher need for energy and reducing power to combat oxidative stress and the possible accumulation of proteins that can not be secreted. Another possibility is related to an eventually compromised electrochemical proton gradient across the membrane. Since TolC is the outer membrane component of many transport systems [1], its inactivation may affect both proton transport and ATP synthesis and possibly the cell responds by increasing expression of genes involved in central metabolism to synthesize more ATP. Although many questions remain unanswered, our results highlight the mechanisms by which a large number of genes act together to restore cell homeostasis and, in particular, points to TolC protein as being fundamental in the biology of this microorganism.

## Methods

### Bacterial strains and growth conditions

Bacterial strains used in this study were wild-type *S. meliloti *1021 (Sm1021) [47], SmLM030-2 (Sm1021, pLS378 integrated into the *tolC *gene region) [15], Sm8530 (Sm1021, *expR*^+^) [48], and Rem::Tn-5 (Sm1021, *rem*^-^) [49]. For gene expression profiling, overnight cultures of *S. meliloti *1021 and *tolC *mutant strain SmLM030-2 grown in TY complex medium [50] were diluted to an initial OD_600 _= 0.1 in GMS medium (Zevenhuizen, 1986). Triplicate flasks of each strain were cultured at 30°C in GMS medium at 180 rpm for 20 hours.

### Isolation and processing of RNA samples

Cells were harvested, resuspended in RNAprotect bacteria reagent (Qiagen), and total RNA extraction was carried out using the RNeasy MiniKit (Qiagen) with DNase treatment following manufacturer's recommendations. Once absence of residual DNA was confirmed, concentration and purity were determined using a Nanodrop ND-1000 UV-visible spectrophotometer. RNA integrity was checked with an Agilent 2100 Bioanalyser using a RNA Nano assay (Agilent Technologies).

RNA was processed for use on Affymetrix (Santa Clara, CA, USA) GeneChip Medicago/Sinorhizobium Genome Arrays, according to the manufacturer's Prokaryotic Target Preparation Assay. Briefly, 10 μg of total RNA containing spiked in Poly-A RNA controls (GeneChip Expression GeneChip Eukaryotic Poly-A RNA Control Kit; Affymetrix) were used in a reverse transcription reaction with random primers (Invitrogen Life Technologies) to generate first-strand cDNA. After removal of RNA, 2 μg of cDNA was fragmented with DNase and end-labeled (GeneChip^® ^WT Terminal Labeling Kit; Affymetrix). Size distribution of the fragmented and end-labeled cDNA, was assessed using an Agilent 2100 Bioanalyzer. 2 μg of end-labeled fragmented cDNA was used in a 200-μl hybridization cocktail containing added hybridization controls and hybridized on arrays for 16 hours at 48°C. Standard post hybridization wash and double-stain protocols (FS450_0001; GeneChip HWS kit, Affymetrix) were used on an Affymetrix GeneChip Fluidics Station 450. Arrays were scanned on an Affymetrix GeneChip scanner 3000 7G.

### Microarray analysis

Scanned arrays were first analyzed using Affymetrix Expression Console software to obtain Absent/Present calls and assure that all quality parameters were in the recommended range. Subsequent analysis was carried out with DNA-Chip Analyzer 2008. First a digital mask was applied, leaving for analysis only the 8305 probe sets on the array representing *Sinorhizobium meliloti *transcripts. Then the 6 arrays were normalized to a baseline array with median CEL intensity by applying an Invariant Set Normalization Method [51]. Normalized CEL intensities of the arrays were used to obtain model-based gene expression indices based on a PM (Perfect Match)-only model [52]. Replicate data (triplicates) for each of the wild-type and *tolC *mutant strains were weighted gene-wise by using inverse squared standard error as weights. Genes compared were considered to be differentially expressed if the 90% lower confidence bound of the fold change between experiment and baseline was above 1.2, resulting in 3155 differentially expressed transcripts with a median False Discovery Rate (FDR) of 0.4%. The lower confidence bound criterion means that we can be 90% confident that the fold change is a value between the lower confidence bound and a variable upper confidence bound. Li and Wong [52] have shown that the lower confidence bound is a conservative estimate of the fold change and therefore more reliable as a ranking statistic for changes in gene expression. For a second analysis Partek Genomics Suite 6.4 was used. Here the 6 arrays were normalized and modeled using Robust Multichip Averaging (RMA). After RMA, probe sets analyzing expression of transcripts of *Medicago truncatula *and *Medicago sativa*, were filtered out. For the remaining *S. meliloti *probe sets differential expression was determined using 1-way Analysis of Variance (ANOVA). FDR analysis with a cut-off of 5% determined 2842 transcripts as differentially expressed, corresponding to an ANOVA p-value cut-off of <0.017. A set of 2067 differentially expressed transcripts was identified in the two independent analyses performed. All further analyses focused on this core set. Fold change values presented in Tables 1 and 2 and in the additional files 1 and 2 were obtained using Partek Genomics Suite 6.4.

### Quantitative real-time RT-PCR

DNA microarray data were validated by quantitative real-time RT-PCR. For reverse transcription 1 μg of total RNA from *S. meliloti *1021 and *tolC *mutant strains, derived from three independent samples, was used. cDNA was synthesized using TaqMan^R ^Reverse Transcription Reagents (Applied Biosystems) according to the manufacturer's instructions. Primers used to amplify selected *S. meliloti *genes (See Additional file 3: Table S3) were designed using Primer Express 3.0 software (Applied Biosystems). RT-PCR amplification mixtures used 400 ng of template cDNA, 2× SYBR Green PCR Master Mix and 0.4 mM of reverse and forward primers for each gene in a total volume of 25 μl. Reactions containing nuclease-free water instead of the reverse transcriptase were included as negative control. Reactions were performed using a model 7500 thermocycler (Applied Biosystems). The expression ratio of the target genes was determined relative to reference gene *hemA*, which showed no variation in the transcript abundance under the experimental conditions used here. Relative quantification of gene expression by real-time RT-PCR was determined by applying the ΔΔCt method [53].

### Preparation of cell lysates and measuring enzymatic activities

*S. meliloti *wild-type and *tolC *mutant cells were grown in GMS medium for 20 hours. Cells were harvested, washed and disrupted by sonication. The total protein concentration was measured by the Bradford method [54]. Catalase and superoxide dismutase activities were determined using the method of Clare et al. [55]. Crude extract (20 μg) of each sample was loaded on a standard nondenaturing polyacrylamide gel and samples electrophoresed for 6 hours at 70 V. To measure catalase activity, the gel was soaked in 50 mg/ml of horseradish peroxidase in 50 mM potassium phosphate, pH 7.0, at room temperature for 45 min and rinsed twice with phosphate buffer. The gel was then incubated with 5.0 mM H_2_O_2 _for 10 min then stained with 0.5 mg/ml diaminobenzidine in phosphate buffer. For superoxide dismutase measurement, the gel was soaked in the dark in 2.5 mM nitro blue tetrazolium with 3 mM H_2_O_2 _supplementation for 20 minutes. Gels were then incubated with 0.028 mM riboflavin and 2.8 mM TEMED in 36 mM phosphate buffer, pH 7.8 for 20 minutes, followed by irradiation with visible light until achromatic bands appeared. Glutathione reductase (GR) activity was measured as described by Smith et al. [56] following the disappearance of NADPH spectrophotometrically at 340 nm (*E *= 6.2 mM^-1 ^cm^-1^). The reaction mixture contained 400 mM phosphate buffer (pH 7.5), 10 mM oxidized glutathione, 1 mM NADPH, 10 mM EDTA, 3 mM Dithionitrobenzoic acid and crude extract.

### Assessment of cells efflux activity

Efflux activity was assayed by ethidium bromide agar screening [57]. Briefly, each *S. meliloti *culture was swabbed onto GMS plates containing ethidium bromide concentrations of 0.5 and 1.0 mg/L. Plates were incubated at 30°C for 48 hours, after which fluorescence under UV light associated with the bacterial mass was recorded.

### Motility assays

Motility assays were carried out as described by Soto et al. [58]. Swimming plates with 0.3% bacto agar (Difco) and swarming plates with 0.6% Noble agar (Difco) were prepared using GMS medium. For estimation of motility, overnight GMS cultures (5 μl) were inoculated on the surface of the agar and incubated at 30°C for 1 and 3 days to measure swarming and swimming motility, respectively. Three separate experiments, each containing two technical replicates were performed.

### Microarray data accession number

The microarray data were deposited in the Array Express database under accession number E-MEXP-2561.

## Authors' contributions

LFM and JDB designed the work, supervised the research study, and prepared the manuscript. MRS, AMC, JMCM and MFM performed all experimental work. All authors read and approved the final manuscript.

## Supplementary Material

Additional file 1 **Genes with increased expression in the *S. meliloti tolC *mutant**. Table S1. Complete list of all *S. meliloti *SmLM030-2 genes with increased expression (>1.2-fold change; p < 0.017) compared to the expression in the wild-type *S. meliloti *1021. Genes classified into COGs are the ones analyzed. Click here for file

Additional file 2 **Genes with decreased expression in the *S. meliloti tolC *mutant**. Table S2. Complete list of all *S. meliloti *SmLM030-2 genes with decreased expression (>1.2-fold change; p < 0.017) compared to expression in the wild-type *S. meliloti *1021. Genes classified into COGs are the ones analyzed. Click here for file

Additional file 3 **Primer sequences used in this study**. Table S3. Gene-specific primers used for real-time RT-PCR. Click here for file
