# Supplementary material for: Absence of functional TolC protein causes increased stress response gene expression in Sinorhizobium meliloti
Source: BMC Microbiol. 2010 Jun 23;10:180. doi: 10.1186/1471-2180-10-180 (PMC2912261; doi:10.1186/1471-2180-10-180)
Supplement: Additional file 1 — Genes with increased expression in the S. meliloti tolC mutant. Table S1. Complete list of all S. meliloti SmLM030-2 genes with increased expression (>1.2-fold change; p < 0.017) compared to the expression in the wild-type S. meliloti 1021. Genes classified into COGs are the ones analyzed. [file 1471-2180-10-180-S1.DOC]

### **Additional file 1 - Table S1. Genes with increased expression in the S. meliloti tolC mutant.**

Complete list of all *S. meliloti* SmLM030-2 genes with increased expression (>1.2-fold change; p<0.017) compared to the expression in the wild-type *S. meliloti* 1021. The genes classified into COGs are the ones analyzed.

| **ID code** | **RMA FC** | **Gene** | **COG** | **Annotation** |
| --- | --- | --- | --- | --- |
| SMc04865 | **1.50** | *-* | *-* | hypothetical protein SMc04865 |
| SMc04480 | **2.14** | *-* | *-* | reapet Sm-1 |
| SMc04461 | **2.79** | *tolB1* | COG0823U | translocation protein TolB |
| SMc04459 | **1.93** | *ftsH* | COG0465O | metalloprotease transmembrane protein |
| SMc04458 | **5.65** | *secA* | COG0653U | preprotein translocase subunit SecA |
| SMc04454 | **4.19** | *-* | COG0488R | putative ABC transporter ATP-binding protein |
| SMc04452 | **4.74/2.59** | *Ndh* | COG1252C | putative NADH dehydrogenase transmembrane protein |
| SMc04450 | **6.83** | *mfd* | COG1197LK | transcription-repair coupling factor (TRCF) protein |
| SMc04429 | **2.23** | *-* | *-* | hypothetical protein SMc04429 |
| SMc04411 | **3.92** | *-* | COG2951M | putative membrane-bound lytic murein transglycosylase signal peptide protein |
| SMc04410 | **5.81/4.72** | *asd* | COG0136E | aspartate-semialdehyde dehydrogenase |
| SMc04405 | **6.72** | *leuB* | COG0473CE | 3-isopropylmalate dehydrogenase |
| SMc04363 | **1.43** | *-* | COG1737K | hypothetical protein SMc04363 |
| SMc04351 | **2.87** | *macB* | COG0577V, COG1136V | transmembrane ATP-binding ABC transporter protein |
| SMc04350 | **4.53** | *macA* | COG0845M | putative multidrug efflux system transmembrane protein |
| SMc04348 | **2.31** | *-* | COG1309K | putative transcription regulator protein |
| SMc04347 | **2.63** | *-* | COG3894R | hypothetical protein SMc04347 |
| SMc04346 | **2.43** | *ilvC* | COG0059EH | ketol-acid reductoisomerase |
| SMc04345 | **1.80** | *-* | *-* | hypothetical protein SMc04345 |
| SMc04342 | **3.45** | *-* | COG1410E | methyltetrahydrofolate:corrinoid/iron-sulfur protein methyltransferase |
| SMc04330 | **2.79** | *mttB1* | COG5598H | putative trimethylamine methyltransferase protein |
| SMc04325 | **3.74** | *-* | COG0646E | methionine synthase I |
| SMc04320 | **2.02** | *rpsU1* | COG0828J | 30S ribosomal protein S21 |
| SMc04319 | **2.73/2.58** | *-* | *-* | hypothetical protein SMc04319 |
| SMc04318 | **2.33/2.08** | *csp1* | COG1278K | cold shock transcription regulator protein |
| SMc04317 | **2.72** | *afuA* | COG1840P | IRON-binding periplasmic protein |
| SMc04316 | **3.25** | *afuB* | COG1178P | iron ABC transporter permease |
| SMc04313 | **2.23** | *-* | COG3668R | hypothetical protein SMc04313 |
| SMc04310 | **5.29** | *-* | *-* | hypothetical protein SMc04310 |
| SMc04302 | **2.32** | *cobO* | COG2109H | Probable Cob(I)alamin adenosyltransferase |
| SMc04286 | **3.82** | *-* | COG0730R | hypothetical protein SMc04286 |
| SMc04284 | **2.09** | *cobA* | COG0007H | uroporphyrin-III C-methyltransferase protein |
| SMc04282 | **3.09** | *cobB* | COG1797H | cobyrinic acid a,c-diamide synthase |
| SMc04278 | **4.00** | *acpXL* | *-* | acyl carrier protein |
| SMc04277 | **3.42** | *-* | COG0764I | putative (3R)-hydroxymyristoyl- |
| SMc04275 | **3.27** | *fabF2* | COG0304IQ | 3-oxoacyl-(acyl carrier protein) synthase II |
| SMc04273 | **3.55** | *-* | COG0304IQ | 3-oxoacyl-(acyl carrier protein) synthase II |
| SMc04272 | **1.98** | *-* | COG0494LR | hypothetical protein SMc04272 |
| SMc04270 | **2.87** | *adhA2* | COG0604CR | putative dehydrogenase protein |
| SMc04268 | **3.99/2.28** | *lpxXL* | COG1560M | lipid A biosynthesis lauroyl acyltransferase |
| SMc04267 | **3.80** | *lpsS* | COG4424S | LPS sulfotransferase |
| SMc04263 | **3.02** | *-* | COG1115E | putative amino acid carrier transmembrane protein |
| SMc04262 | **2.56** | *gnd* | COG0362G | 6-phosphogluconate dehydrogenase |
| SMc04261 | **2.59** | *-* | *-* | hypothetical protein SMc04261 |
| SMc04260 | **2.42** | *-* | COG1609K | putative transcription regulator protein |
| SMc04252 | **2.23** | *-* | COG0667C | putative oxidoreductase protein |
| SMc04251 | **1.82** | *smoM* | COG4663Q | putative mannitol-binding periplasmic signal peptide protein |
| SMc04247 | **5.84** | *-* | COG3507G | putative BETA-xylosidase protein |
| SMc04239 | **1.74** | *-* | *-* | hypothetical protein SMc04239 |
| SMc04234 | **1.26** | *csp4* | COG1278K | putative cold shock-like transcription regulator protein |
| SMc04231 | **2.31** | *uvrB* | COG0556L | excinuclease ABC subunit B |
| SMc04230 | **2.15** | *-* | COG3169S | putative transmembrane signal peptide protein |
| SMc04219 | **2.75** | *-* | COG1131V | ABC transporter ATP-binding protein |
| SMc04218 | **3.04** | *-* | COG0842V | putative transport transmembrane protein |
| SMc04217 | **2.34** | *-* | COG3577R | putative transmembrane signal peptide protein |
| SMc04216 | **2.40** | *-* | COG3313R | hypothetical protein SMc04216 |
| SMc04214 | **2.91** | *cobU* | COG2038H | nicotinate-nucleotide-dimethylbenzimidazole phosphoribosyltransferase |
| SMc04213 | **2.47** | *dgkA* | COG0818M | diacylglycerol kinase protein |
| SMc04209 | **7.35** | *-* | *-* | hypothetical protein SMc04209 |
| SMc04202 | **2.33** | *-* | *-* | putative transmembrane protein |
| SMc04199 | **1.88** | *-* | *-* | hypothetical protein SMc04199 |
| SMc04185 | **2.68** | *-* | COG0463M | putative glycosyltransferase protein |
| SMc04175 | **1.80** | *-* | COG0861P | putative transmembrane protein |
| SMc04164 | **2.10** | *-* | *-* | hypothetical protein SMc04164 |
| SMc04128 | **1.59** | *-* | COG2217P | putative heavy metal transporting ATPase protein |
| SMc04094 | **3.31/2.95** | *-* | COG3820S | hypothetical protein SMc04094 |
| SMc04093 | **1.79** | *acsA1* | COG0365I | acetyl-CoA synthetase |
| SMc04092 | **2.44** | *-* | *-* | hypothetical protein SMc04092 |
| SMc04091 | **1.57** | *htpX* | COG0501O | heat shock protein HtpX |
| SMc04090 | **2.63** | *rsmB1* | *-* | putative sun protein |
| SMc04088 | **3.86** | *purH* | COG0138F | bifunctional phosphoribosylaminoimidazolecarboxamide formyltransferase/IMP cyclohydrolase |
| SMc04085 | **1.71** | *-* | COG2902E | hypothetical protein SMc04085 |
| SMc04045 | **2.09** | *ilvD2* | COG0129EG | dihydroxy-acid dehydratase |
| SMc04042 | **1.98** | *-* | COG0483G | putative monophosphatase protein |
| SMc04041 | **1.81** | *pldB* | COG2267I | lysophospholipase L2 protein |
| SMc04040 | **6.66** | *ibpA* | COG0071O | heat shock protein |
| SMc04030 | **3.92** | *-* | COG0385R | hypothetical protein SMc04030 |
| SMc04029 | **2.25** | *-* | COG2008E | putative low specificity L-threonine aldolase protein |
| SMc04020 | **1.95** | *-* | COG0330O | hypothetical protein SMc04020 |
| SMc04019 | **2.48** | *hemH* | *-* | ferrochelatase |
| SMc04016 | **1.97** | *hss* | COG5310Q | homospermidine synthase protein |
| SMc04012 | **1.57** | *pepF* | COG1164E | putative oligoendopeptidase F protein |
| SMc04009 | **3.43/2.80** | *-* | COG3750S | hypothetical protein SMc04009 |
| SMc04007 | **1.93** | *-* | COG3931E | hypothetical protein SMc04007 |
| SMc04006 | **1.75** | *-* | COG5480S | hypothetical protein SMc04006 |
| SMc04005 | **1.66** | *pykA* | COG0469G | pyruvate kinase |
| SMc04003 | **4.81** | *rpmJ* | COG0257J | 50S ribosomal protein L36 |
| SMc04001 | **1.68** | *purE* | COG0041F | phosphoribosylaminoimidazole carboxylase catalytic subunit protein |
| SMc04000 | **3.33/1.55** | *-* | *-* | hypothetical protein SMc04000 |
| SMc03997 | **2.82** | *-* | COG0730R | hypothetical protein SMc03997 |
| SMc03996 | **2.48** | *thiE2* | COG0352H | thiamine-phosphate pyrophosphorylase |
| SMc03995 | **3.73** | *-* | COG0790R | hypothetical protein SMc03995 |
| SMc03994 | **2.13** | *suhB* | COG0483G | putative inositol monophosphatase protein |
| SMc03991 | **4.01** | *-* | COG1132V | ABC transporter ATP-binding protein |
| SMc03990 | **4.46** | *rpmE* | COG0254J | 50S ribosomal protein L31 |
| SMc03986 | **2.30** | *-* | *-* | hypothetical protein SMc03986 |
| SMc03985 | **1.98** | *cyaF2* | COG0457R, COG2114T | adenylate/guanylate cyclase protein |
| SMc03983 | **4.07** | *fbaB* | COG3588G | fructose-bisphosphate aldolase class I protein |
| SMc03981 | **4.94** | *pgk* | COG0126G | phosphoglycerate kinase |
| SMc03980 | **4.30** | *-* | COG3554S | hypothetical protein SMc03980 |
| SMc03979 | **3.83/2.70** | *gap* | COG0057G | glyceraldehyde-3-phosphate dehydrogenase |
| SMc03978 | **4.19/3.74** | *tkt2* | COG0021G | transketolase |
| SMc03975 | **2.24** | *-* | COG0583K | putative transcription regulator protein |
| SMc03974 | **2.08** | *-* | COG0212H | hypothetical protein SMc03974 |
| SMc03973 | **2.90** | *-* | COG1692S | hypothetical protein SMc03973 |
| SMc03969 | **6.72/4.10** | *-* | COG0217S | hypothetical protein SMc03969 |
| SMc03968 | **2.54** | *-* | COG2141C | putative oxidoreductase protein |
| SMc03967 | **2.00** | *ruvC* | COG0817L | Holliday junction resolvase |
| SMc03957 | **1.61** | *tolR* | COG0848U | putative transport transmembrane protein |
| SMc03956 | **2.01** | *tolA* | *-* | putative signal peptide protein |
| SMc03950 | **5.46** | *pntAa* | COG3288C | proton-translocating nicotinamide nucleotide transhydrogenase subunit protein |
| SMc03941 | **2.43** | *-* | *-* | hypothetical protein SMc03941 |
| SMc03939 | **2.96** | *pntAb* | COG3288C | NAD(P) transhydrogenase subunit alpha |
| SMc03938 | **5.13** | *pntB* | COG1282C | NAD(P) transhydrogenase subunit beta |
| SMc03934 | **2.15** | *rpsU* | *-* | 30S ribosomal protein S21 |
| SMc03933 | **2.87** | *soxB2* | COG0665E | putative sarcosine oxidase subunit B protein |
| SMc03932 | **3.81** | *soxD2* | COG4311E | putative sarcosine oxidase delta subunit protein |
| SMc03928 | **1.92** | *-* | COG4427S | hypothetical protein SMc03928 |
| SMc03927 | **1.79** | *nodN2* | COG2030I | putative nodulation protein |
| SMc03926 | **1.80** | *glgX1* | COG1523G | glycosyl hydrolase protein |
| SMc03925 | **1.43** | *pgm* | COG0033G | phosphoglucomutase |
| SMc03924 | **2.05** | *glgA* | COG0297G | glycogen synthase |
| SMc03923 | **2.03** | *glgC* | COG0448G | glucose-1-phosphate adenylyltransferase |
| SMc03900 | **1.73** | *ndvA* | COG1132V | cyclic beta-1,2-glucan ABC transporter |
| SMc03891 | **2.88** | *-* | COG0834ET | putative amino acid-binding periplasmic ABC transporter protein |
| SMc03888 | **2.59** | *ispG* | COG0821I | 4-hydroxy-3-methylbut-2-en-1-yl diphosphate synthase |
| SMc03887 | **2.00** | *-* | COG1280E | putative amino acid efflux protein |
| SMc03885 | **2.04** | *hisC2* | COG0079E | histidinol-phosphate aminotransferase |
| SMc03884 | **1.62** | *ispA* | COG0142H | geranyltranstransferase protein |
| SMc03881 | **4.22** | *rpmF* | COG0333J | 50S ribosomal protein L32 |
| SMc03877 | **7.30** | *-* | COG0513LKJ | Putative ATP-dependent RNA helicase |
| SMc03876 | **3.71** | *-* | COG1188J | Heat shock protein 15 (HSP15) |
| SMc03875 | **2.65** | *-* | COG1146C | putative ferredoxin protein |
| SMc03872 | **1.93** | *-* | COG0501O, COG4784R | hypothetical protein SMc03872 |
| SMc03867 | **2.81** | *-* | COG1564H | hypothetical protein SMc03867 |
| SMc03866 | **4.84** | *-* | COG0488R | ABC transporter ATP-binding protein |
| SMc03864 | **1.52** | *-* | COG0834ET | putative amino acid-binding periplasmic (signal peptide) ABC transporter protein |
| SMc03863 | **3.82** | *rplS* | COG0335J | 50S ribosomal protein L19 |
| SMc03862 | **2.39/2.02** | *-* | COG0730R | hypothetical protein SMc03862 |
| SMc03860 | **4.35** | *rimM* | COG0806J | 16S rRNA-processing protein |
| SMc03859 | **8.24/5.03** | *rpsP* | COG0228J | 30S ribosomal protein S16 |
| SMc03858 | **8.44** | *pheAa* | COG1605E | chorismate mutase |
| SMc03857 | **5.29** | *ffh* | COG0541U | signal recognition particle protein |
| SMc03856 | **4.54** | *dapF* | COG0253E | diaminopimelate epimerase |
| SMc03855 | **4.55** | *-* | COG0621J | hypothetical protein SMc03855 |
| SMc03854 | **4.17** | *ftsY* | COG0552U | putative cell division protein |
| SMc03853 | **2.31** | *ispZ* | COG2917D | intracellular septation protein A |
| SMc03851 | **1.98** | *ccmG* | COG0526OC | putative thiol:disulfide interchange protein (cytochrome C biogenesis protein) |
| SMc03849 | **1.71** | *ccmC* | COG0755O | putative HEME exporter C (cytochrome C-type biogenesis protein) transmembrane |
| SMc03848 | **2.44** | *ccmB* | COG2386O | putative HEME exporter B (cytochrome C-type biogenesis protein) transmembrane |
| SMc03846 | **3.30** | *acnA* | COG1048C | aconitate hydratase |
| SMc03845 | **3.25/2.01** | *-* | COG5429S | hypothetical protein SMc03845 |
| SMc03838 | **3.52** | *-* | COG3127Q | hypothetical protein SMc03838 |
| SMc03837 | **2.66** | *-* | COG4181Q | ABC transporter ATP-binding protein |
| SMc03836 | **1.80** | *tesA* | *-* | putative acyl-CoA thioesterase i protein |
| SMc03833 | **3.33/2.88** | *-* | COG3339S | hypothetical protein SMc03833 |
| SMc03831 | **3.93** | *rlmN* | COG0820R | Putative ribosomal RNA large subunit methyltransferase N |
| SMc03830 | **3.65** | *-* | COG2984R | hypothetical protein SMc03830 |
| SMc03829 | **4.55** | *-* | *-* | putative transport system permease ABC transporter protein |
| SMc03828 | **3.46** | *-* | COG1101R | ABC transporter ATP-binding protein |
| SMc03826 | **3.09** | *argG* | COG0137E | argininosuccinate synthase |
| SMc03818 | **3.31** | *xthA3* | COG0708L | putative exodeoxyribonuclease III protein |
| SMc03804 | **1.58** | *-* | COG0654HC | 2-octaprenyl-6-methoxyphenyl hydroxylase |
| SMc03802 | **2.63** | *-* | COG2802R | hypothetical protein SMc03802 |
| SMc03801 | **3.12** | *-* | COG3118O | putative thioredoxin protein |
| SMc03797 | **2.28** | *metA* | COG1897E | homoserine O-succinyltransferase |
| SMc03796 | **3.17** | *-* | COG0251J | hypothetical protein SMc03796 |
| SMc03795 | **3.08** | *leuD* | COG0066E | isopropylmalate isomerase small subunit |
| SMc03791 | **3.10** | *-* | COG3824S | hypothetical protein SMc03791 |
| SMc03785 | **1.85** | *ialA* | COG0494LR | dinucleoside polyphosphate hydrolase |
| SMc03784 | **2.26** | *-* | COG2861S | hypothetical protein SMc03784 |
| SMc03782 | **1.90** | *-* | COG4942D | putative signal peptide protein |
| SMc03775 | **1.87** | *cgtA* | *-* | GTPase ObgE |
| SMc03772 | **1.68** | *rpmA* | COG0211J | 50S ribosomal protein L27 |
| SMc03770 | **1.67** | *rplU* | COG0261J | 50S ribosomal protein L21 |
| SMc03746 | **3.85** | *-* | *-* | hypothetical protein SMc03746 |
| SMc03744 | **2.29** | *-* | COG2960S | hypothetical protein SMc03744 |
| SMc03297 | **2.53** | *-* | *-* | hypothetical protein SMc03297 |
| SMc03277 | **3.18** | *-* | COG0477GEPR | putative transport transmembrane protein |
| SMc03267 | **2.51** | *-* | COG2355E | putative dipeptidase protein |
| SMc03252 | **2.09** | *proB2* | COG0263E | Putative glutamate 5-kinase |
| SMc03251 | **4.51** | *-* | COG3367S | hypothetical protein SMc03251 |
| SMc03242 | **14.43** | *typA* | COG1217T | GTP-binding protein |
| SMc03241 | **4.29** | *-* | COG0454KR | hypothetical protein SMc03241 |
| SMc03238 | **3.49** | *-* | COG0762S | hypothetical protein SMc03238 |
| SMc03229 | **3.22** | *gpsA* | COG0240C | glycerol-3-phosphate dehydrogenase protein |
| SMc03228 | **5.45** | *-* | COG2350S | YciI-like protein |
| SMc03227 | **2.84** | *-* | COG2947S | hypothetical protein SMc03227 |
| SMc03226 | **2.26** | *-* | *-* | hypothetical protein SMc03226 |
| SMc03205 | **2.44** | *purU1* | COG0788F | formyltetrahydrofolate deformylase |
| SMc03202 | **2.98** | *bkdAb* | COG0022C | 2-oxoisovalerate dehydrogenase beta subunit |
| SMc03201 | **6.29** | *bkdAa* | COG1071C | 2-oxoisovalerate dehydrogenase alpha subunit protein |
| SMc03198 | **1.92** | *modC* | COG4148P | molybdenum ABC transporter ATP-binding protein |
| SMc03196 | **1.35** | *modA* | COG0725P | molybdate ABC transporter periplasmic molybdate-binding protein |
| SMc03192 | **2.29** | *cobH* | COG2082H | precorrin-8X methylmutase |
| SMc03171 | **2.39** | *-* | COG0861P | hypothetical protein SMc03171 |
| SMc03170 | **4.73** | *-* | COG2207K, COG3708S | putative transcription regulator protein |
| SMc03169 | **4.86/4.79** | *-* | COG1309K | putative transcription regulator protein |
| SMc03168 | **41.50** | *-* | COG1566V | putative multidrug efflux system protein |
| SMc03167 | **41.14** | *-* | COG0477GEPR | putative multidrug efflux system protein |
| SMc03166 | **4.48** | *-* | COG5555N | hypothetical protein SMc03166 |
| SMc03163 | **5.14** | *xylA* | COG2115G | xylose isomerase |
| SMc03159 | **4.45** | *metN* | COG1135P | ABC transporter ATP-binding protein |
| SMc03158 | **12.29** | *metI* | COG2011P | ABC transporter permease |
| SMc03157 | **14.91/8.66** | *metQ* | COG1464P | putative outer membrane lipoprotein transmembrane |
| SMc03156 | **3.05** | *-* | COG1054R | hypothetical protein SMc03156 |
| SMc03152 | **2.50/2.35** | *-* | COG2979S | hypothetical protein SMc03152 |
| SMc03151 | **2.42** | *-* | COG1670J | hypothetical protein SMc03151 |
| SMc03127 | **3.43** | *-* | COG0601EP | putative transport system permease ABC transporter protein |
| SMc03125 | **2.22** | *-* | COG0444EP | putative transport system ATP-binding ABC transporter protein |
| SMc03124 | **3.26** | *-* | COG0747E | putative periplasmic binding ABC transporter protein |
| SMc03112 | **2.39/1.73** | *metH* | COG0646E, COG1410E | B12-dependent methionine synthase |
| SMc03110 | **2.97** | *-* | COG4702S | hypothetical protein SMc03110 |
| SMc03109 | **3.45** | *mak* | COG1940KG | Putative fructokinase |
| SMc03096 | **2.04** | *-* | COG2911S | putative signal peptide protein |
| SMc03072 | **6.64** | *-* | *-* | hypothetical protein SMc03072 |
| SMc03071 | **4.64** | *-* | COG3951MNO | hypothetical protein SMc03071 |
| SMc03064 | **3.80** | *aglA* | COG0366G | alpha-glucosidase protein |
| SMc03057 | **7.84** | *-* | *-* | hypothetical protein SMc03057 |
| SMc03056 | **2.02** | *-* | *-* | hypothetical protein SMc03056 |
| SMc03053 | **2.09** | *fliQ* | COG1987NU | flagellar biosynthesis protein FliQ |
| SMc03052 | **2.17** | *flgD* | COG1843N | flagellar basal body rod modification protein |
| SMc03051 | **5.68** | *flbT* | *-* | flagellar biosynthesis repressor FlbT |
| SMc03050 | **7.22** | *flaF* | *-* | flagellar biosynthesis regulatory protein FlaF |
| SMc03049 | **7.12** | *flgL* | COG1344N | flagellar hook-associated protein FlgL |
| SMc03048 | **6.89** | *flgK* | COG1256N | flagellar hook-associated protein FlgK |
| SMc03047 | **8.06** | *flgE* | COG1749N | flagellar hook protein FlgE |
| SMc03046 | **4.24** | *rem* | COG0745TK | putative transcription regulator protein |
| SMc03045 | **5.94** | *-* | COG0741M | hypothetical protein SMc03045 |
| SMc03044 | **6.33** | *fliK* | COG3144N | chemotaxis protein (motility protein D) |
| SMc03042 | **3.40** | *motB* | COG1360N | flagellar motor protein MotB |
| SMc03041 | **7.88** | *-* | *-* | hypothetical protein SMc03041 |
| SMc03040 | **3.68** | *flaC* | COG1344N | flagellin protein |
| SMc03036 | **3.50** | *fliP* | COG1338NU | flagellar biosynthesis protein FliP |
| SMc03035 | **6.12** | *fliL* | *-* | flagellar transmembrane protein |
| SMc03034 | **3.82** | *flgH* | COG2063N | flagellar basal body L-ring protein |
| SMc03032 | **6.73** | *flgI* | COG1706N | flagellar basal body P-ring protein |
| SMc03031 | **3.46** | *flgA* | COG1261NO | flagellar basal body P-ring biosynthesis protein FlgA |
| SMc03030 | **11.03/4.06** | *flgG* | COG4786N | flagellar basal body rod protein FlgG |
| SMc03029 | **7.08** | *fliE* | COG1677NU | flagellar hook-basal body protein FliE |
| SMc03028 | **12.85** | *flgC* | COG1558N | flagellar basal body rod protein FlgC |
| SMc03027 | **9.30** | *flgB* | COG1815N | flagellar basal body rod protein FlgB |
| SMc03025 | **4.69** | *fliI* | COG1157NU | flagellum-specific ATP synthase |
| SMc03024 | **15.64** | *flgF* | COG4786N | flagellar basal body rod protein FlgF |
| SMc03023 | **13.92** | *-* | COG0001H | hypothetical protein SMc03023 |
| SMc03022 | **16.20** | *motA* | COG1291N | flagellar motor protein MotA |
| SMc03021 | **3.75** | *fliM* | COG1868N | flagellar motor switch transmembrane protein |
| SMc03020 | **6.89** | *fliN* | COG1886NU | flagellar motor switch protein |
| SMc03019 | **5.74** | *fliG* | COG1536N | flagellar motor switch protein G |
| SMc03018 | **4.96** | *flhB* | COG1377NU | flagellar biosynthesis protein FlhB |
| SMc03017 | **2.61** | *-* | *-* | hypothetical protein SMc03017 |
| SMc03014 | **8.30** | *fliF* | COG1766NU | flagellar MS-ring protein |
| SMc03013 | **2.24** | *-* | *-* | hypothetical protein SMc03013 |
| SMc03012 | **2.59** | *cheD* | COG1871NT | chemoreceptor glutamine deamidase CheD |
| SMc03010 | **1.71** | *cheB* | COG2201NT | chemotaxis-specific methylesterase |
| SMc03009 | **1.94** | *cheR* | COG1352NT | chemotaxis protein methyltransferase |
| SMc03008 | **1.76** | *cheW1* | COG0835NT | chemotaxis protein |
| SMc03006 | **1.55** | *cheY1* | COG0784T | chemotaxis regulator protein |
| SMc03005 | **1.53** | *-* | *-* | hypothetical protein SMc03005 |
| SMc02989 | **2.34/2.13** | *-* | *-* | hypothetical protein SMc02989 |
| SMc02984 | **1.96** | *-* | COG0583K | putative transcription regulator protein |
| SMc02983 | **1.82** | *-* | COG0019E | putative ornithine, DAP, or arginine decarboxylase protein |
| SMc02982 | **1.97** | *-* | COG3153R | hypothetical protein SMc02982 |
| SMc02980 | **3.16** | *-* | *-* | hypothetical protein SMc02980 |
| SMc02978 | **2.96** | *-* | COG0523R | hypothetical protein SMc02978 |
| SMc02975 | **1.69** | *pckR* | COG1609K | putative phosphoenolpyruvate carboxykinase regulator transcription regulator protein |
| SMc02941 | **2.18** | *-* | COG1729S | hypothetical protein SMc02941 |
| SMc02940 | **2.78** | *tilS* | COG0037D | Putative tRNA(Ile)-lysidine synthase |
| SMc02914 | **4.92/4.65** | *infB* | COG0532J | translation initiation factor IF-2 |
| SMc02913 | **4.97** | *-* | COG2740K | hypothetical protein SMc02913 |
| SMc02912 | **5.71** | *nusA* | COG0195K | transcription elongation factor NusA |
| SMc02911 | **6.11/4.36** | *-* | COG0779S | hypothetical protein SMc02911 |
| SMc02909 | **3.07** | *mltB1* | COG2951M | putative transglycosylase transmembrane protein |
| SMc02908 | **2.60** | *recR* | COG0353L | recombination protein RecR |
| SMc02906 | **2.50** | *-* | COG0718S | hypothetical protein SMc02906 |
| SMc02905 | **4.35/2.50** | *dnaX* | COG2812L | Putative DNA polymerase III subunit TAU protein |
| SMc02898 | **2.57/2.40** | *kdsB* | COG1212M | 3-deoxy-manno-octulosonate cytidylyltransferase |
| SMc02897 | **4.20** | *-* | COG3474C | putative cytochrome C transmembrane protein |
| SMc02890 | **3.74** | *-* | COG1629P | putative outer membrane receptor protein |
| SMc02886 | **2.24/1.54** | *-* | *-* | putative signal peptide protein |
| SMc02885 | **1.77** | *msrA1* | COG0225O | methionine sulfoxide reductase A |
| SMc02883 | **3.19** | *-* | COG2900S | hypothetical protein SMc02883 |
| SMc02882 | **4.26/3.31** | *-* | COG2981E | CysZ-like protein |
| SMc02868 | **3.94/2.84** | *-* | COG0845M | putative multidrug efflux system protein |
| SMc02867 | **5.85/2.67** | *-* | COG0841V | putative multidrug-efflux system transmembrane protein |
| SMc02866 | **4.17** | *-* | COG1309K | putative transcription regulator protein |
| SMc02865 | **2.20** | *-* | COG0454KR | putative acetyltransferase protein |
| SMc02862 | **1.80** | *-* | COG1392P | Pit accessory protein |
| SMc02861 | **2.23** | *pit* | COG0306P | phosphate transporter |
| SMc02857 | **2.69** | *dnaK* | COG0443O | molecular chaperone DnaK |
| SMc02855 | **2.67** | *-* | COG0697GER | hypothetical protein SMc02855 |
| SMc02850 | **1.94** | *polA* | COG0258L, COG0749L | DNA polymerase I |
| SMc02849 | **1.77** | *gyaR* | COG1052CHR | 2-hydroxyacid dehydrogenase |
| SMc02846 | **3.13** | *-* | COG0524G | putative sugar kinase protein |
| SMc02844 | **1.68** | *-* | COG1309K | putative transcription regulator protein |
| SMc02838 | **2.08** | *gpmA* | COG0588G | phosphoglyceromutase |
| SMc02837 | **2.14** | *dapB* | COG0289E | dihydrodipicolinate reductase |
| SMc02836 | **4.03** | *-* | COG1132V | ABC transporter ATP-binding protein |
| SMc02831 | **1.91** | *-* | COG4174R | ABC transporter permease |
| SMc02821 | **2.01** | *-* | COG4965U | hypothetical protein SMc02821 |
| SMc02803 | **1.25** | *-* | *-* | hypothetical protein SMc02803 |
| SMc02802 | **2.12** | *holA* | COG1466L | DNA polymerase III subunit delta |
| SMc02801 | **1.84** | *parB* | COG1475K | chromosome partitioning protein ParB |
| SMc02798 | **2.31** | *mnmG* | COG0486R | tRNA uridine 5-carboxymethylaminomethyl modification enzyme GidA |
| SMc02796 | **2.20** | *rho* | COG1158K | transcription termination factor Rho |
| SMc02795 | **3.70** | *-* | COG0407H | hypothetical protein SMc02795 |
| SMc02792 | **1.93** | *maf* | COG0424D | Maf-like protein |
| SMc02791 | **1.69** | *aroE* | COG0169E | shikimate 5-dehydrogenase |
| SMc02788 | **4.85** | *secB* | COG1952U | preprotein translocase subunit SecB |
| SMc02786 | **1.28** | *-* | COG4395S | putative translocase transmembrane protein |
| SMc02785 | **2.53** | *-* | COG2821M | putative lytic murein transglycosylase A protein |
| SMc02780 | **3.21** | *gabD1* | COG1012C | succinate-semialdehyde dehydrogenase |
| SMc02769 | **2.05** | *-* | *-* | hypothetical protein SMc02769 |
| SMc02768 | **2.94** | *-* | COG1451R | hypothetical protein SMc02768 |
| SMc02767 | **3.37** | *trpF* | COG0135E | N-(5'-phosphoribosyl)anthranilate isomerase |
| SMc02766 | **3.10** | *trpB* | COG0133E | tryptophan synthase subunit beta |
| SMc02765 | **3.72** | *trpA* | COG0159E | tryptophan synthase subunit alpha |
| SMc02764 | **2.85** | *accD* | COG0777I | acetyl-CoA carboxylase subunit beta |
| SMc02763 | **3.67** | *folC* | COG0285H | bifunctional folylpolyglutamate synthase/dihydrofolate synthase |
| SMc02761 | **2.03** | *trxA* | COG0526OC | thioredoxin protein |
| SMc02760 | **2.75** | *-* | COG1074L | putative ATP-dependent nuclease/helicase protein |
| SMc02757 | **2.10** | *-* | COG0802R, COG3178R | hypothetical protein SMc02757 |
| SMc02755 | **1.80** | *ahcY* | COG0499H | S-adenosyl-L-homocysteine hydrolase |
| SMc02753 | **3.12** | *-* | COG2893G | putative IIA component of PTS system protein |
| SMc02735 | **1.49** | *-* | *-* | hypothetical protein SMc02735 |
| SMc02734 | **2.03** | *-* | *-* | hypothetical protein SMc02734 |
| SMc02732 | **1.71** | *-* | COG3385L | hypothetical protein SMc02732 |
| SMc02724 | **3.32** | *-* | COG0053P | hypothetical protein SMc02724 |
| SMc02723 | **2.75** | *queF* | COG0780R | 7-cyano-7-deazaguanine reductase |
| SMc02722 | **3.01** | *-* | COG3231J | putative aminoglycoside 3'-phosphotransferase protein |
| SMc02720 | **1.65** | *clpP2* | COG3385L | ATP-dependent Clp protease proteolytic subunit |
| SMc02707 | **2.17** | *-* | COG3153R | hypothetical protein SMc02707 |
| SMc02701 | **2.81** | *nudL* | COG0494LR | Nudix hydrolase family protein |
| SMc02697 | **3.07** | *-* | COG2030I | hypothetical protein SMc02697 |
| SMc02696 | **3.19** | *-* | COG2030I | hypothetical protein SMc02696 |
| SMc02695 | **4.91** | *engD* | COG0012J | Putative GTP-dependent nucleic acid-binding protein EngD |
| SMc02692 | **7.73/6.38** | *rplY* | COG1825J | 50S ribosomal protein L25/general stress protein Ctc |
| SMc02686 | **7.39** | *prsA* | COG0462FE | ribose-phosphate pyrophosphokinase |
| SMc02682 | **3.40** | *-* | COG1565S | hypothetical protein SMc02682 |
| SMc02681 | **3.42** | *lgt* | COG0682M | prolipoprotein diacylglyceryl transferase |
| SMc02659 | **2.58** | *relA* | COG0317TK | putative GTP pyrophosphokinase (ATP:GTP 3'-pyrophosphotransferase) protein |
| SMc02657 | **2.82** | *-* | *-* | hypothetical protein SMc02657 |
| SMc02655 | **2.30** | *-* | COG3216S | hypothetical protein SMc02655 |
| SMc02654 | **2.95** | *acpS* | COG0736I | 4'-phosphopantetheinyl transferase |
| SMc02653 | **3.07** | *lepB* | COG0681U | signal peptidase I transmembrane protein |
| SMc02651 | **2.65** | *era* | COG1159R | GTP-binding protein Era |
| SMc02646 | **1.99** | *-* | COG0584C | hypothetical protein SMc02646 |
| SMc02636 | **7.73** | *-* | COG1376S | hypothetical protein SMc02636 |
| SMc02603 | **1.49** | *-* | COG0477GEPR | putative transport transmembrane protein |
| SMc02601 | **1.66** | *nadA* | COG0379H | quinolinate synthetase |
| SMc02599 | **2.21** | *nadB* | COG0029H | L-aspartate oxidase |
| SMc02598 | **2.91** | *nadC* | COG0157H | nicotinate-nucleotide pyrophosphorylase carboxylating protein |
| SMc02585 | **1.52** | *actS* | COG0642T | sensor histidine kinase transmembrane protein |
| SMc02584 | **2.06/2.06** | *actR* | COG4567TK | transcription regulator protein |
| SMc02582 | **4.99** | *-* | COG1376S | hypothetical protein SMc02582 |
| SMc02577 | **3.45** | *hslU* | COG1220O | ATP-dependent protease ATP-binding subunit |
| SMc02576 | **5.71/4.54** | *-* | COG1670J | acetyltransferase |
| SMc02575 | **5.20** | *hslV* | COG5405O | ATP-dependent protease peptidase subunit |
| SMc02574 | **2.25** | *hisB* | COG0131E | imidazoleglycerol-phosphate dehydratase |
| SMc02573 | **3.21** | *-* | *-* | hypothetical protein SMc02573 |
| SMc02572 | **3.36** | *hisH* | COG0118E | imidazole glycerol phosphate synthase subunit HisH |
| SMc02570 | **2.89** | *hisA* | COG0106E | 1-(5-phosphoribosyl)-5- |
| SMc02569 | **2.36** | *hisF* | COG0107E | imidazole glycerol phosphate synthase subunit HisF |
| SMc02568 | **2.39** | *hisE* | COG0140E | phosphoribosyl-ATP pyrophosphatase |
| SMc02567 | **2.25** | *coaA* | COG1072H | pantothenate kinase |
| SMc02563 | **3.84** | *-* | COG1186J | hypothetical protein SMc02563 |
| SMc02562 | **4.36/3.10** | *pckA* | COG1866C | phosphoenolpyruvate carboxykinase |
| SMc02558 | **2.55** | *-* | COG0354R | hypothetical protein SMc02558 |
| SMc02557 | **3.09** | *-* | *-* | hypothetical protein SMc02557 |
| SMc02552 | **2.97** | *-* | *-* | hypothetical protein SMc02552 |
| SMc02548 | **2.03** | *-* | COG3791S | hypothetical protein SMc02548 |
| SMc02547 | **2.25** | *pip1* | *-* | putative proline iminopeptidase protein |
| SMc02545 | **6.08** | *-* | COG2962R | hypothetical protein SMc02545 |
| SMc02519 | **1.59** | *-* | COG3839G | ABC transporter ATP-binding protein |
| SMc02509 | **9.39** | *sitA* | COG0803P | putative IRON-binding periplasmic ABC transporter protein |
| SMc02508 | **4.96** | *sitB* | COG1121P | putative IRON transport ATP-binding ABC transporter protein |
| SMc02507 | **3.05** | *sitC* | COG1108P | putative IRON transport system membrane ABC transporter protein |
| SMc02506 | **5.42** | *sitD* | COG1108P | putative IRON transport system membrane ABC transporter protein |
| SMc02503 | **3.98** | *-* | COG1748E | hypothetical protein SMc02503 |
| SMc02502 | **9.84** | *atpC* | COG0355C | F0F1 ATP synthase subunit epsilon |
| SMc02501 | **7.69** | *atpD* | COG0055C | F0F1 ATP synthase subunit beta |
| SMc02500 | **16.24/11.08** | *atpG* | COG0224C | F0F1 ATP synthase subunit gamma |
| SMc02499 | **8.18/4.32** | *atpA* | COG0056C | F0F1 ATP synthase subunit alpha |
| SMc02498 | **7.19** | *atpH* | *-* | F0F1 ATP synthase subunit delta |
| SMc02487 | **2.47** | *lpdA2* | COG1249C | dihydrolipoamide dehydrogenase |
| SMc02486 | **2.41** | *-* | COG1028IQR | putative oxidoreductase protein |
| SMc02484 | **1.56** | *-* | COG1280E | putative amino acid efflux transmembrane protein |
| SMc02483 | **5.73/3.84** | *sucB* | COG0508C | dihydrolipoamide acetyltransferase |
| SMc02482 | **7.17** | *sucA* | COG0567C | alpha-ketoglutarate decarboxylase |
| SMc02481 | **9.31** | *sucD* | COG0074C | succinyl-CoA synthetase subunit alpha |
| SMc02480 | **9.42/9.37** | *sucC* | COG0045C | succinyl-CoA synthetase subunit beta |
| SMc02479 | **9.87** | *mdh* | COG0039C | malate dehydrogenase |
| SMc02471 | **3.21** | *-* | COG1653G | putative periplasmic binding ABC transporter protein |
| SMc02469 | **2.42** | *-* | COG0673R | putative oxidoreductase protein |
| SMc02466 | **2.99** | *sdhB* | COG0479C | succinate dehydrogenase iron-sulfur subunit |
| SMc02465 | **3.21** | *sdhA* | COG1053C | succinate dehydrogenase flavoprotein subunit |
| SMc02464 | **2.86** | *sdhD* | COG2142C | succinate dehydrogenase membrane anchor subunit protein |
| SMc02463 | **3.86** | *Sdh* | COG2009C | succinate dehydrogenase cytochrome B-556 subunit transmembrane protein |
| SMc02451 | **2.27** | *-* | COG0760O | hypothetical protein SMc02451 |
| SMc02450 | **2.83** | *argJ* | *-* | bifunctional ornithine acetyltransferase/N-acetylglutamate synthase protein |
| SMc02448 | **2.38** | *mutT* | COG0494LR | putative mutator protein 7,8-dihydro-8-oxoguanine-triphosphatase |
| SMc02445 | **2.32** | *-* | COG0500QR | hypothetical protein SMc02445 |
| SMc02443 | **4.27** | *grxC* | COG0695O | glutaredoxin 3 protein |
| SMc02442 | **4.15** | *-* | COG0388R | putative hydrolase protein |
| SMc02441 | **1.93** | *-* | COG5319S | hypothetical protein SMc02441 |
| SMc02440 | **1.95** | *ubiG* | COG2227H | 3-demethylubiquinone-9 3-methyltransferase |
| SMc02438 | **5.56** | *lysC* | COG0527E | aspartate kinase |
| SMc02437 | **2.18** | *ptsP* | COG3605T | phosphoenolpyruvate phosphotransferase PTSP (enzyme I-Ntr) protein |
| SMc02436 | **3.65** | *prfA* | COG0216J | peptide chain release factor 1 |
| SMc02435 | **3.23** | *hemK1* | COG2890J | putative methyltransferase protein |
| SMc02434 | **2.40** | *-* | *-* | hypothetical protein SMc02434 |
| SMc02433 | **2.40** | *clpB* | COG0542O | ATP-dependent protease |
| SMc02432 | **2.92** | *-* | COG0739M | hypothetical protein SMc02432 |
| SMc02408 | **3.26** | *rpoZ* | COG1758K | DNA-directed RNA polymerase subunit omega |
| SMc02407 | **1.88** | *-* | COG1432S | hypothetical protein SMc02407 |
| SMc02405 | **3.54** | *smpB* | COG0691O | SsrA-binding protein |
| SMc02404 | **3.15** | *dapA* | COG0329EM | dihydrodipicolinate synthase |
| SMc02391 | **3.34** | *-* | *-* | hypothetical protein SMc02391 |
| SMc02390 | **2.94** | *gst7* | COG0625O | putative glutathione S-transferase protein |
| SMc02389 | **2.40** | *-* | *-* | hypothetical protein SMc02389 |
| SMc02388 | **2.48** | *-* | *-* | hypothetical protein SMc02388 |
| SMc02384 | **2.10** | *-* | COG0463M | putative glycosyltransferase transmembrane protein |
| SMc02382 | **2.25** | *-* | COG1597IR | hypothetical protein SMc02382 |
| SMc02372 | **4.77** | *-* | COG0477GEPR | putative transport transmembrane protein |
| SMc02368 | **5.60** | *glnE* | COG1391OT | putative glutamate-ammonia-ligase adenylyltransferase protein |
| SMc02367 | **3.66** | *-* | COG0642T | putative sensor histidine kinase transmembrane protein |
| SMc02366 | **4.95** | *ragA* | COG0745TK | Probable response regulator |
| SMc02365 | **20.37/18.53** | *degP1* | COG0265O | protease precursor protein |
| SMc02363 | **1.84** | *cycK* | COG1138O | cytochrome C-type biogenesis transmembrane protein |
| SMc02351 | **4.89** | *-* | COG2346R | hypothetical protein SMc02351 |
| SMc02350 | **2.75** | *-* | COG0526OC | hypothetical protein SMc02350 |
| SMc02349 | **3.47** | *asfA* | COG1053C | putative oxidoreductase protein |
| SMc02347 | **3.64** | *asfB* | COG1146C | putative ferredoxin ASFB IRON-sulfur protein |
| SMc02346 | **3.17** | *-* | COG1116P | Putative glycine-betaine and choline ABC transporter, ATP-binding component |
| SMc02345 | **3.34/2.98** | *-* | COG0600P | Putative glycine-betaine and choline ABC transporter, permease component |
| SMc02344 | **3.99/3.93** | *-* | COG0715P | Putative glycine-betaine and choline ABC transporter, periplasmic solute-binding component |
| SMc02343 | **4.01** | *-* | COG0477GEPR | putative transport transmembrane protein |
| SMc02312 | **2.79** | *-* | COG3024S | zinc-binding protein |
| SMc02311 | **4.22** | *maf* | COG0424D | Maf-like protein |
| SMc02310 | **5.74** | *infA* | COG0361J | translation initiation factor IF-1 |
| SMc02308 | **2.00** | *-* | *-* | hypothetical protein SMc02308 |
| SMc02307 | **2.68** | *hisD* | COG0141E | histidinol dehydrogenase |
| SMc02306 | **2.28** | *-* | *-* | hypothetical protein SMc02306 |
| SMc02305 | **2.76** | *murA* | COG0766M | UDP-N-acetylglucosamine 1-carboxyvinyltransferase |
| SMc02278 | **3.72** | *-* | *-* | hypothetical protein SMc02278 |
| SMc02274 | **1.93** | *rkpU* | *-* | capsule polysaccharide exporter protein |
| SMc02273 | **2.09** | *rkpA* | COG3321Q | fatty acid synthase transmembrane protein |
| SMc02272 | **2.52** | *rkpG* | COG0156H | acyl-transferase transferase protein |
| SMc02270 | **2.17** | *rkpI* | COG1368M | capsular polysaccharide biosynthesis\export transmembrane protein |
| SMc02268 | **2.15** | *kpsF3* | COG0517R, COG0794M | putative capsule expression protein |
| SMc02265 | **6.42** | *secD2* | COG0341U, COG0342U | bifunctional preprotein translocase subunit SecD/SecF |
| SMc02264 | **8.57/3.95** | *-* | *-* | hypothetical protein SMc02264 |
| SMc02255 | **1.80** | *qxtA* | COG1271C | putative quinol oxidase subunit I transmembrane protein |
| SMc02253 | **3.35** | *pchB* | COG1605E | putative salicylate biosynthesis protein |
| SMc02248 | **1.85** | *-* | COG2197TK | putative transcription regulator protein |
| SMc02245 | **1.77** | *pyrD* | COG0167F | dihydroorotate dehydrogenase 2 |
| SMc02243 | **2.93** | *-* | COG2340S | hypothetical protein SMc02243 |
| SMc02242 | **2.82** | *-* | *-* | hypothetical protein SMc02242 |
| SMc02235 | **2.44** | *-* | COG1522K | putative transcription regulator protein |
| SMc02221 | **4.76** | *-* | *-* | hypothetical protein SMc02221 |
| SMc02217 | **3.03** | *metZ* | COG0626E | O-succinylhomoserine sulfhydrylase |
| SMc02203 | **1.96** | *-* | *-* | hypothetical protein SMc02203 |
| SMc02202 | **2.36** | *-* | *-* | hypothetical protein SMc02202 |
| SMc02199 | **1.98** | *-* | *-* | hypothetical protein SMc02199 |
| SMc02178 | **5.12** | *-* | *-* | hypothetical protein SMc02178 |
| SMc02177 | **2.75** | *-* | *-* | hypothetical protein SMc02177 |
| SMc02166 | **2.01** | *pyrC* | COG0418F | dihydroorotase |
| SMc02165 | **1.67** | *pyrE* | COG0461F | orotate phosphoribosyltransferase |
| SMc02164 | **1.93** | *frk* | COG0524G | fructokinase protein |
| SMc02160 | **2.28** | *-* | *-* | ABC transporter permease |
| SMc02155 | **2.13** | *-* | COG1483R | hypothetical protein SMc02155 |
| SMc02154 | **1.98** | *-* | COG1743L | hypothetical protein SMc02154 |
| SMc02146 | **1.56** | *-* | COG0226P | putative phosphate-binding periplasmic protein |
| SMc02145 | **3.34/1.74** | *-* | *-* | putative signal peptide protein |
| SMc02143 | **3.03** | *pstA* | COG0581P | phosphate ABC transporter permease |
| SMc02142 | **2.86** | *pstB* | COG1117P | phosphate ABC transporter ATP-binding protein |
| SMc02141 | **2.87** | *phoU* | COG0704P | phosphate transporter PhoU |
| SMc02140 | **2.91** | *phoB* | COG0745TK | phosphate regulon transcriptional regulatory protein |
| SMc02137 | **2.11** | *argF1* | COG0078E | ornithine carbamoyltransferase |
| SMc02136 | **5.06** | *hslO* | COG1281O | Hsp33-like chaperonin |
| SMc02124 | **20.18** | *cysI* | COG0155P | putative nitrite reductase protein |
| SMc02123 | **12.60** | *-* | COG3749S | hypothetical protein SMc02123 |
| SMc02122 | **4.16** | *fpr* | COG1018C | ferredoxin--NADP reductase |
| SMc02112 | **2.12** | *-* | *-* | hypothetical protein SMc02112 |
| SMc02108 | **2.06** | *-* | *-* | hypothetical protein SMc02108 |
| SMc02101 | **13.67** | *rpsB* | COG0052J | 30S ribosomal protein S2 |
| SMc02100 | **10.79/5.58** | *tsf* | COG0264J | elongation factor Ts |
| SMc02099 | **2.91/2.42** | *pyrH* | COG0528F | uridylate kinase |
| SMc02097 | **1.42** | *uppS* | COG0020I | putative undecaprenyl pyrophosphate synthetase protein |
| SMc02096 | **2.27** | *cdsA* | COG0575I | phosphatidate cytidylyltransferase transmembrane protein |
| SMc02095 | **2.59** | *-* | COG0750M | hypothetical protein SMc02095 |
| SMc02094 | **1.84** | *omp* | COG4775M | putative outer membrane transmembrane protein |
| SMc02093 | **2.77** | *lpxD* | COG1044M | Probable UDP-3-O-3-hydroxymyristoyl glucosamine N-acyltransferase |
| SMc02092 | **2.71** | *fabZ* | COG0764I | (3R)-hydroxymyristoyl-ACP dehydratase |
| SMc02091 | **3.03** | *lpxA* | COG1043M | UDP-N-acetylglucosamine acyltransferase |
| SMc02090 | **2.80** | *-* | COG3494S | hypothetical protein SMc02090 |
| SMc02085 | **2.61** | *exbB* | COG0811U | biopolymer transport transmembrane protein |
| SMc02084 | **3.60** | *exbD* | COG0848U | biopolymer transport transmembrane protein |
| SMc02082 | **1.37** | *tolC* | COG1538MU | putative outer membrane secretion protein |
| SMc02078 | **1.88** | *exoR* | COG0790R | exopolysaccharide biosynthesis regulatory protein |
| SMc02075 | **1.32** | *-* | COG0316S | hypothetical protein SMc02075 |
| SMc02068 | **2.08** | *-* | COG3842E | putative ABC transporter ATP-binding protein |
| SMc02067 | **3.18/2.54** | *tatA* | COG1826U | twin argininte translocase protein A |
| SMc02063 | **1.71** | *surE* | COG0496R | stationary phase survival protein SurE |
| SMc02061 | **5.15** | *bioS* | *-* | biotin-regulated protein |
| SMc02058 | **2.59** | *-* | COG1862U | YAJC protein |
| SMc02057 | **2.72/2.56** | *secD1* | COG0341U, COG0342U | bifunctional preprotein translocase subunit SecD/SecF |
| SMc02056 | **2.44** | *-* | COG3737S | hypothetical protein SMc02056 |
| SMc02055 | **3.41** | *crtB* | COG1562I | putative phytoene synthase protein |
| SMc02053 | **10.39** | *trmFO* | COG1206J | Methylenetetrahydrofolate--tRNA-(uracil-5-)-methyltransferase |
| SMc02052 | **9.49** | *-* | COG5457S | hypothetical protein SMc02052 |
| SMc02050 | **9.14/5.32** | *tig* | COG0544O | trigger factor |
| SMc01942 | **2.28** | *ureD* | COG0829O | urease accessory protein |
| SMc01935 | **4.14** | *lolC* | COG4591M | Putative lipoprotein-releasing system transmembrane protein |
| SMc01934 | **2.95** | *proS* | COG0442J | prolyl-tRNA synthetase |
| SMc01931 | **1.64** | *-* | *-* | hypothetical protein SMc01931 |
| SMc01930 | **2.76** | *-* | COG0346E | hypothetical protein SMc01930 |
| SMc01929 | **4.27** | *-* | *-* | hypothetical protein SMc01929 |
| SMc01928 | **3.18** | *birA* | COG0340H | Probable biotin--[acetyl-CoA-carboxylase] synthetase |
| SMc01927 | **6.64** | *nuoN* | COG1007C | NADH dehydrogenase subunit N |
| SMc01926 | **7.79** | *nuoM* | COG1008C | NADH dehydrogenase subunit M |
| SMc01925 | **10.00** | *nuoL* | COG1009CP | NADH dehydrogenase subunit L |
| SMc01924 | **7.59** | *nuoK1* | COG0713C | NADH dehydrogenase subunit K |
| SMc01923 | **9.12** | *nuoJ* | COG0839C | NADH dehydrogenase subunit J |
| SMc01922 | **7.86** | *nuoI* | COG1143C | NADH dehydrogenase subunit I |
| SMc01921 | **6.16** | *nuoH* | COG1005C | NADH dehydrogenase subunit H |
| SMc01920 | **5.78** | *nuoG1* | COG1034C | NADH dehydrogenase subunit G |
| SMc01919 | **3.11** | *-* | *-* | hypothetical protein SMc01919 |
| SMc01918 | **5.99/5.05** | *nuoF1* | COG1894C | NADH dehydrogenase I subunit F |
| SMc01917 | **5.77** | *nuoE1* | COG1905C | NADH dehydrogenase subunit E |
| SMc01915 | **6.41** | *nuoD1* | COG0649C | NADH dehydrogenase subunit D |
| SMc01914 | **7.70** | *nuoC1* | COG0852C | NADH dehydrogenase subunit C |
| SMc01913 | **3.95** | *nuoB1* | COG0377C | NADH dehydrogenase subunit B |
| SMc01912 | **5.83** | *nuoA1* | COG0838C | NADH dehydrogenase subunit A |
| SMc01910 | **3.96** | *-* | COG4717S | hypothetical protein SMc01910 |
| SMc01909 | **4.74** | *-* | COG0420L | hypothetical protein SMc01909 |
| SMc01907 | **4.88/3.44** | *-* | COG4222S | hypothetical protein SMc01907 |
| SMc01906 | **1.75** | *hrm* | COG0776L | histone-like protein |
| SMc01905 | **2.52/1.60** | *lon* | COG0466O | ATP-dependent protease LA protein |
| SMc01904 | **1.17** | *clpX* | COG1219O | ATP-dependent protease ATP-binding subunit |
| SMc01903 | **2.50** | *clpP* | COG0740OU | ATP-dependent Clp protease proteolytic subunit |
| SMc01902 | **3.22** | *-* | *-* | hypothetical protein SMc01902 |
| SMc01881 | **2.80** | *panB* | COG0413H | 3-methyl-2-oxobutanoate hydroxymethyltransferase |
| SMc01880 | **3.65** | *panC* | COG0414H | pantoate--beta-alanine ligase |
| SMc01877 | **3.53** | *recN* | COG0497L | DNA repair protein |
| SMc01876 | **2.02** | *-* | COG4105R | hypothetical protein SMc01876 |
| SMc01869 | **4.38** | *-* | COG0477GEPR | putative transport transmembrane protein |
| SMc01867 | **2.24** | *murC* | COG0773M | UDP-N-acetylmuramate--L-alanine ligase |
| SMc01866 | **1.93** | *murG* | COG0707M | N-acetylglucosaminyl transferase |
| SMc01862 | **1.92** | *murF* | COG0770M | UDP-N-acetylmuramoylalanyl-D-glutamyl-2, 6-diaminopimelate--D-alanyl-D-alanyl ligase protein |
| SMc01861 | **1.87** | *murE* | COG0769M | UDP-N-acetylmuramoylalanyl-D-glutamate--2,6-diaminopimelate ligase |
| SMc01856 | **2.04** | *-* | COG0665E | putative sarcosine oxidase protein |
| SMc01855 | **3.20** | *-* | COG0741M | hypothetical protein SMc01855 |
| SMc01854 | **3.36** | *-* | COG3023V | putative amidase (AMPD protein) |
| SMc01853 | **2.14** | *-* | COG1076O | hypothetical protein SMc01853 |
| SMc01845 | **1.84** | *-* | COG2951M | putative transglycosylase transmembrane protein |
| SMc01843 | **1.89** | *metF* | COG0685E | 5,10-methylenetetrahydrofolate reductase oxidoreductase protein |
| SMc01842 | **2.78** | *-* | COG0500QR, COG0640K | putative methyltransferase transcription regulator protein |
| SMc01834 | **2.56** | *-* | COG0678O | hypothetical protein SMc01834 |
| SMc01827 | **1.90** | *-* | COG0715P | Putative uracil and uridine ABC transporter, periplasmic solute-binding protein |
| SMc01817 | **2.33** | *-* | COG0583K | putative transcription regulator protein |
| SMc01811 | **1.98** | *TRm20C* | *-* | putative partial transposase protein |
| SMc01809 | **1.68** | *-* | COG2873E | O-acetylhomoserine aminocarboxypropyltransferase |
| SMc01808 | **1.42** | *-* | COG1832R | hypothetical protein SMc01808 |
| SMc01806 | **2.20** | *-* | COG1024I | enoyl-CoA hydratase |
| SMc01804 | **7.06** | *rplM* | COG0102J | 50S ribosomal protein L13 |
| SMc01803 | **6.49** | *rpsI* | COG0103J | 30S ribosomal protein S9 |
| SMc01802 | **1.94** | *speB* | COG0010E | agmatinase |
| SMc01800 | **3.44** | *-* | COG1612O | putative cytochrome C oxidase assembly transmembrane protein |
| SMc01799 | **2.87** | *-* | *-* | putative signal peptide protein |
| SMc01785 | **2.18** | *fabH* | COG0332I | 3-oxoacyl-(acyl carrier protein) synthase III |
| SMc01784 | **3.34** | *plsX* | COG0416I | putative glycerol-3-phosphate acyltransferase PlsX |
| SMc01783 | **1.65** | *-* | *-* | hypothetical protein SMc01783 |
| SMc01782 | **3.48/1.99** | *-* | *-* | hypothetical protein SMc01782 |
| SMc01781 | **2.32** | *-* | COG2913J | hypothetical protein SMc01781 |
| SMc01779 | **4.34** | *-* | COG0477GEPR | putative transport transmembrane protein |
| SMc01777 | **3.79** | *ribH* | COG0054H | riboflavin synthase subunit beta |
| SMc01774 | **6.33** | *-* | *-* | hypothetical protein SMc01774 |
| SMc01773 | **3.38** | *ribE* | COG0307H | riboflavin synthase subunit alpha |
| SMc01772 | **3.04** | *ribD* | COG0117H, COG1985H | riboflavin biosynthesis protein |
| SMc01771 | **2.70** | *-* | COG1327K | transcriptional regulator NrdR |
| SMc01770 | **4.38/3.84/3.68** | *glyA* | COG0112E | serine hydroxymethyltransferase |
| SMc01767 | **2.11** | *-* | *-* | hypothetical protein SMc01767 |
| SMc01766 | **2.71** | *hemB* | COG0113H | delta-aminolevulinic acid dehydratase |
| SMc01765 | **2.27** | *-* | COG1714S | hypothetical protein SMc01765 |
| SMc01763 | **1.77** | *-* | COG0697GER | hypothetical protein SMc01763 |
| SMc01756 | **3.90** | *aspS* | COG0173J | aspartyl-tRNA synthetase |
| SMc01755 | **3.46** | *-* | COG0477GEPR | putative transport transmembrane protein |
| SMc01754 | **2.43** | *-* | COG2132Q | putative oxidoreductase protein |
| SMc01732 | **1.56** | *dapD* | *-* | 2,3,4,5-tetrahydropyridine-2-carboxylate N-succinyltransferase |
| SMc01731 | **5.77/3.72** | *-* | COG1611R | hypothetical protein SMc01731 |
| SMc01730 | **3.08** | *-* | COG1011R | hypothetical protein SMc01730 |
| SMc01729 | **1.91** | *-* | COG0697GER | hypothetical protein SMc01729 |
| SMc01726 | **4.40** | *argB* | COG0548E | acetylglutamate kinase |
| SMc01722 | **2.39** | *engB* | COG0218R | GTPase EngB |
| SMc01721 | **4.17** | *-* | COG0706U | putative inner membrane protein translocase component YidC |
| SMc01720 | **7.93** | *rnpA* | COG0594J | ribonuclease P |
| SMc01704 | **2.73** | *-* | COG2020O | hypothetical protein SMc01704 |
| SMc01700 | **6.94/6.14** | *ppiA* | COG0652O | putative peptidyl-prolyl cis-trans isomerase A signal peptide protein |
| SMc01660 | **3.49** | *-* | *-* | hypothetical protein SMc01660 |
| SMc01659 | **4.12** | *-* | COG0614P | putative transport transmembrane protein |
| SMc01658 | **2.98** | *-* | COG4114R | putative transport protein |
| SMc01657 | **2.32** | *fhuA2* | COG1629P | putative ferrichrome-IRON receptor precursor protein |
| SMc01628 | **3.31** | *-* | COG1653G | putative periplasmic binding ABC transporter protein |
| SMc01611 | **5.78** | *fhuA1* | COG1629P | putative ferrichrome-IRON receptor precursor protein |
| SMc01587 | **1.51** | *-* | COG0491R | hypothetical protein SMc01587 |
| SMc01584 | **4.22** | *-* | COG0697GER | hypothetical protein SMc01584 |
| SMc01580 | **2.10** | *-* | *-* | hypothetical protein SMc01580 |
| SMc01578 | **4.89** | *aatA* | COG0436E | aspartate aminotransferase |
| SMc01573 | **2.05** | *-* | COG3453S | hypothetical protein SMc01573 |
| SMc01569 | **2.60** | *carA* | COG0505EF | carbamoyl phosphate synthase small subunit |
| SMc01568 | **1.92** | *-* | COG1610S | hypothetical protein SMc01568 |
| SMc01563 | **3.30/2.15** | *rpoD* | COG0568K | RNA polymerase sigma factor RpoD |
| SMc01557 | **2.42** | *-* | *-* | putative signal peptide protein |
| SMc01547 | **4.55** | *-* | *-* | hypothetical protein SMc01547 |
| SMc01545 | **1.94** | *-* | COG0454KR | hypothetical protein SMc01545 |
| SMc01529 | **1.65** | *dppF2* | COG1124EP | peptide ABC transporter ATP-binding protein |
| SMc01524 | **1.77** | *-* | COG2355E | putative dipeptidase protein |
| SMc01523 | **4.79** | *emrE* | COG2076P | putative methyl viologen/ethidium resistance transmembrane protein |
| SMc01522 | **3.82** | *-* | COG1309K | hypothetical protein SMc01522 |
| SMc01520 | **3.12** | *-* | COG2346R | hypothetical protein SMc01520 |
| SMc01519 | **2.56** | *-* | COG2363S | hypothetical protein SMc01519 |
| SMc01518 | **2.33** | *-* | COG2329R | hypothetical protein SMc01518 |
| SMc01517 | **3.17** | *-* | *-* | hypothetical protein SMc01517 |
| SMc01516 | **2.00** | *-* | *-* | hypothetical protein SMc01516 |
| SMc01515 | **1.92** | *-* | COG0810M | hypothetical protein SMc01515 |
| SMc01512 | **1.16** | *hmuT* | *-* | putative hemin binding periplasmic transmembrane protein |
| SMc01511 | **1.77** | *hmuU* | COG0609P | putative hemin transport system permease transmembrane protein |
| SMc01503 | **2.15** | *-* | COG1069C | putative sugar kinase protein |
| SMc01502 | **4.02** | *-* | COG0637R | putative hydrolase phosphatase protein |
| SMc01501 | **4.03** | *mtlK* | COG0246G | mannitol 2-dehydrogenase protein |
| SMc01500 | **4.22** | *smoS* | COG1028IQR | sorbitol dehydrogenase |
| SMc01499 | **4.27** | *smoK* | COG3839G | ATP-binding transport ABC transporter protein |
| SMc01498 | **3.86** | *smoG* | COG0395G | sorbitol/mannitol transport inner membrane protein |
| SMc01497 | **3.94** | *smoF* | COG1175G | sorbitol/mannitol transport inner membrane transmembrane protein |
| SMc01496 | **1.94** | *smoE* | COG1653G | sorbitol-binding periplasmic protein |
| SMc01488 | **3.25** | *-* | *-* | hypothetical protein SMc01488 |
| SMc01472 | **3.67** | *prmA* | COG2264J | ribosomal protein L11 methyltransferase |
| SMc01471 | **2.59** | *senC* | COG1999R | putative cytochrome C oxidase assembly factor transmembrane protein |
| SMc01468 | **1.64** | *cheW2* | *-* | chemotaxis protein |
| SMc01466 | **4.22/2.13** | *TRm21* | COG5433L | transposase |
| SMc01465 | **3.82** | *creA* | COG3045S | putative CREA protein |
| SMc01457 | **4.21** | *-* | COG0841V | putative transport transmembrane protein |
| SMc01454 | **1.87** | *-* | *-* | hypothetical protein SMc01454 |
| SMc01452 | **1.97/1.68** | *-* | COG4321R | hypothetical protein SMc01452 |
| SMc01441 | **3.31** | *hflK* | COG0330O | putative membrane bound protease protein |
| SMc01440 | **2.43** | *hflC* | COG0330O | putative hydrolase serine protease transmembrane protein |
| SMc01439 | **1.91** | *-* | COG3603S | hypothetical protein SMc01439 |
| SMc01437 | **2.34** | *-* | COG1670J | putative acetyl transferase protein |
| SMc01431 | **3.27** | *ilvI* | COG0028EH | acetolactate synthase 3 catalytic subunit |
| SMc01430 | **3.68** | *ilvH* | COG0440E | acetolactate synthase 3 regulatory subunit |
| SMc01428 | **2.94** | *cspA2* | COG1278K | cold shock transcription regulator protein |
| SMc01427 | **3.20** | *-* | *-* | hypothetical protein SMc01427 |
| SMc01406 | **1.88** | *-* | COG1167KE | putative transcription regulator protein |
| SMc01376 | **4.69** | *lolD* | COG1136V | putative lipoprotein-releasing system ATP-binding protein |
| SMc01369 | **4.47** | *rpmG* | COG0267J | 50S ribosomal protein L33 |
| SMc01368 | **5.58** | *-* | COG0477GEPR | putative transport transmembrane protein |
| SMc01366 | **3.10** | *-* | *-* | hypothetical protein SMc01366 |
| SMc01365 | **4.01** | *rnr* | COG0557K | putative exoribonuclease II protein |
| SMc01364 | **3.41** | *topA* | COG0550L, COG1754R | DNA topoisomerase I |
| SMc01362 | **2.82** | *-* | COG0344S | putative glycerol-3-phosphate acyltransferase PlsY |
| SMc01361 | **5.73** | *-* | COG0044F | dihydroorotase |
| SMc01349 | **3.03** | *-* | COG0454KR | hypothetical protein SMc01349 |
| SMc01348 | **1.58** | *-* | *-* | NADH dehydrogenase |
| SMc01347 | **2.07** | *-* | COG4765S | hypothetical protein SMc01347 |
| SMc01345 | **3.17** | *accC* | *-* | acetyl-CoA carboxylase biotin carboxylase subunit |
| SMc01344 | **2.25** | *accB* | COG0511I | acetyl-CoA carboxylase biotin carboxyl carrier protein subunit |
| SMc01343 | **2.38/2.24** | *aroQ* | COG0757E | 3-dehydroquinate dehydratase |
| SMc01342 | **1.93** | *-* | COG1651O | hypothetical protein SMc01342 |
| SMc01336 | **3.43** | *rne* | COG1530J | ribonuclease E protein |
| SMc01334 | **1.79** | *mrcA1* | COG5009M | penicillin-binding 1A transmembrane protein |
| SMc01333 | **7.22** | *prfB* | COG1186J | peptide chain release factor 2 |
| SMc01329 | **2.28** | *acpD* | COG1182I | acyl carrier protein phosphodiesterase |
| SMc01327 | **2.79** | *-* | COG0566J | putative tRNA/rRNA methyltransferase protein |
| SMc01326 | **10.16/10.09** | *tuf* | COG0050J | elongation factor Tu |
| SMc01323 | **2.41** | *secE* | COG0690U | preprotein translocase subunit SecE |
| SMc01322 | **5.05/3.72** | *nusG* | COG0250K | transcription antitermination protein NusG |
| SMc01321 | **4.52** | *rplK* | COG0080J | 50S ribosomal protein L11 |
| SMc01320 | **5.63** | *rplA* | COG0081J | 50S ribosomal protein L1 |
| SMc01319 | **5.94** | *rplJ* | COG0244J | 50S ribosomal protein L10 |
| SMc01318 | **6.28/4.42** | *rplL* | COG0222J | 50S ribosomal protein L7/L12 |
| SMc01317 | **4.25** | *rpoB* | COG0085K | DNA-directed RNA polymerase subunit beta |
| SMc01316 | **3.86** | *rpoC* | COG0086K | DNA-directed RNA polymerase subunit beta' |
| SMc01315 | **2.07** | *-* | *-* | hypothetical protein SMc01315 |
| SMc01314 | **19.54** | *rpsL* | COG0048J | 30S ribosomal protein S12 |
| SMc01313 | **30.44** | *rpsG* | COG0049J | 30S ribosomal protein S7 |
| SMc01312 | **29.55/20.98** | *fusA1* | COG0480J | elongation factor G |
| SMc01310 | **25.56/19.70** | *rpsJ* | COG0051J | 30S ribosomal protein S10 |
| SMc01309 | **22.35/16.47** | *rplC* | COG0087J | 50S ribosomal protein L3 |
| SMc01308 | **24.06** | *rplD* | COG0088J | 50S ribosomal protein L4 |
| SMc01307 | **31.88** | *rplW* | COG0089J | 50S ribosomal protein L23 |
| SMc01306 | **20.50/18.05** | *rplB* | COG0090J | 50S ribosomal protein L2 |
| SMc01305 | **15.22** | *rpsS* | COG0185J | 30S ribosomal protein S19 |
| SMc01304 | **13.24** | *rplV* | COG0091J | 50S ribosomal protein L22 |
| SMc01303 | **17.47/10.55** | *rpsC* | COG0092J | 30S ribosomal protein S3 |
| SMc01302 | **12.36** | *rplP* | COG0197J | 50S ribosomal protein L16 |
| SMc01301 | **13.08** | *rpmC* | COG0255J | 50S ribosomal protein L29 |
| SMc01300 | **20.80/11.99** | *rpsQ* | COG0186J | 30S ribosomal protein S17 |
| SMc01299 | **16.14/13.24** | *rplN* | COG0093J | 50S ribosomal protein L14 |
| SMc01298 | **13.09** | *rplX* | COG0198J | 50S ribosomal protein L24 |
| SMc01297 | **15.38/5.75** | *rplE* | COG0094J | 50S ribosomal protein L5 |
| SMc01296 | **13.26** | *rpsN* | COG0199J | 30S ribosomal protein S14 |
| SMc01295 | **12.87** | *rpsH* | COG0096J | 30S ribosomal protein S8 |
| SMc01294 | **12.32** | *rplF* | COG0097J | 50S ribosomal protein L6 |
| SMc01293 | **24.68/12.53** | *rplR* | COG0256J | 50S ribosomal protein L18 |
| SMc01292 | **15.91/6.68** | *rpsE* | COG0098J | 30S ribosomal protein S5 |
| SMc01291 | **12.92** | *rpmD* | COG1841J | 50S ribosomal protein L30 |
| SMc01290 | **10.47/7.67** | *rplO* | COG0200J | 50S ribosomal protein L15 |
| SMc01289 | **7.01** | *secY* | COG0201U | preprotein translocase subunit SecY |
| SMc01288 | **3.30/2.90** | *adk* | *-* | adenylate kinase |
| SMc01287 | **8.52/5.65** | *rpsM* | COG0099J | 30S ribosomal protein S13 |
| SMc01286 | **6.90/4.70** | *rpsK* | COG0100J | 30S ribosomal protein S11 |
| SMc01285 | **4.57** | *rpoA* | COG0202K | DNA-directed RNA polymerase subunit alpha |
| SMc01283 | **4.29** | *rplQ* | COG0203J | 50S ribosomal protein L17 |
| SMc01280 | **1.90** | *degP3* | COG0265O | protease protein |
| SMc01278 | **2.08** | *sugE* | COG2076P | chaperone homologue transmembrane protein |
| SMc01276 | **2.24** | *gph2* | COG0546R | putative phosphoglycolate phosphatase protein |
| SMc01275 | **2.28** | *rluC* | COG0564J | ribosomal large subunit pseudouridine synthase C protein |
| SMc01270 | **1.70** | *adhC1* | COG1062C | alcohol dehydrogenase class III/glutathione-dependent formaldehyde dehydrogenase protein |
| SMc01268 | **5.81** | *lipB* | COG0321H | lipoyltransferase |
| SMc01263 | **1.68** | *-* | *-* | hypothetical protein SMc01263 |
| SMc01260 | **2.36** | *-* | COG0789K | putative transcription regulator protein |
| SMc01258 | **2.18** | *-* | COG3503S | hypothetical protein SMc01258 |
| SMc01256 | **2.05** | *sda* | COG1760E | L-serine dehydratase |
| SMc01242 | **6.65** | *-* | COG2847S, COG4549S | signal peptide protein |
| SMc01236 | **2.41** | *-* | COG1801S | hypothetical protein SMc01236 |
| SMc01235 | **2.37** | *uvrA* | COG0178L | excinuclease ABC subunit A |
| SMc01233 | **2.37** | *ssb* | COG0629L | single-strand DNA-binding protein |
| SMc01231 | **1.72** | *gyrA* | COG0188L | DNA gyrase subunit A |
| SMc01228 | **3.57** | *-* | COG3570V | putative antibiotic resistance (kinase) protein |
| SMc01227 | **2.84** | *nerA* | COG1902C | putative glycerol trinitrate (GTN) reductase protein |
| SMc01226 | **5.63** | *-* | COG0640K | putative transcription regulator protein |
| SMc01225 | **3.37/1.63** | *lsrB* | COG0583K | Transcription regulator LysR family |
| SMc01224 | **2.04** | *trxB* | COG0492O | thioredoxin reductase protein |
| SMc01222 | **3.72** | *lpsC* | COG0463M | putative lipopolysaccharide core biosynthesis glycosyl transferase protein |
| SMc01221 | **4.16** | *lpsD* | COG0438M | putative lipopolysaccharide core biosynthesis glycosyl transferase protein |
| SMc01220 | **4.07** | *lpsE* | COG0438M | putative lipopolysaccharide core biosynthesis glycosyl transferase protein |
| SMc01219 | **2.76/2.13** | *lpsB* | COG0438M | putative lipopolysaccharide core biosynthesis mannosyltransferase protein |
| SMc01216 | **2.67** | *-* | *-* | hypothetical protein SMc01216 |
| SMc01215 | **2.86** | *carB* | COG0458EF | carbamoyl phosphate synthase large subunit |
| SMc01212 | **2.03** | *-* | COG0477GEPR | putative transport transmembrane protein |
| SMc01211 | **3.98** | *-* | COG0534V | hypothetical protein SMc01211 |
| SMc01210 | **3.10** | *-* | *-* | hypothetical protein SMc01210 |
| SMc01209 | **5.26** | *coaD* | COG0669H | phosphopantetheine adenylyltransferase |
| SMc01208 | **3.71** | *ppiB* | COG0652O | putative peptidyl-prolyl cis-trans isomerase B protein |
| SMc01205 | **1.77** | *-* | COG0834ET | putative amino-acid-binding periplasmic signal peptide protein |
| SMc01204 | **2.51** | *-* | COG0300R | putative transmembrane oxidoreductase protein |
| SMc01203 | **2.11** | *-* | COG2020O | hypothetical protein SMc01203 |
| SMc01193 | **2.51** | *-* | COG0084L | hypothetical protein SMc01193 |
| SMc01188 | **1.97** | *dac* | COG1686M | putative penicillin-binding precursor (D-alanyl-D-alanine carboxypeptidase fraction A) transmembrane protein |
| SMc01180 | **5.03** | *-* | *-* | hypothetical protein SMc01180 |
| SMc01179 | **4.07** | *-* | COG2076P | putative multidrug transmembrane resistance signal peptide protein |
| SMc01174 | **2.20** | *cysK2* | COG0031E | cysteine synthase A |
| SMc01172 | **1.81** | *sseA* | COG2897P | putative thiosulfate sulfurtransferase protein |
| SMc01171 | **2.20** | *-* | COG0454KR | hypothetical protein SMc01171 |
| SMc01170 | **3.45** | *-* | *-* | hypothetical protein SMc01170 |
| SMc01169 | **26.17** | *ald* | COG0686E | alanine dehydrogenase oxidoreductase protein |
| SMc01155 | **2.51** | *ubiE* | COG2226H | ubiquinone/menaquinone biosynthesis methyltransferase |
| SMc01154 | **2.13** | *fpg* | COG0266L | formamidopyrimidine-DNA glycosylase |
| SMc01152 | **6.28** | *rpsT* | COG0268J | 30S ribosomal protein S20 |
| SMc01147 | **3.76** | *-* | COG0635H | coproporphyrinogen III oxidase |
| SMc01146 | **2.96** | *-* | COG0127F | putative deoxyribonucleotide triphosphate pyrophosphatase |
| SMc01144 | **2.80** | *rph* | COG0689J | ribonuclease PH |
| SMc01142 | **3.03** | *grpE* | COG0576O | heat shock protein |
| SMc01138 | **3.97** | *-* | COG1137R | ABC transporter ATP-binding protein |
| SMc01137 | **1.86** | *-* | COG1934S | hypothetical protein SMc01137 |
| SMc01136 | **5.08** | *-* | COG5375S | hypothetical protein SMc01136 |
| SMc01131 | **1.90** | *-* | COG1092R | hypothetical protein SMc01131 |
| SMc01130 | **1.88** | *-* | COG0566J | putative tRNA/rRNA methyltransferase protein |
| SMc01127 | **3.68** | *-* | COG3176R | hypothetical protein SMc01127 |
| SMc01126 | **2.55** | *tme* | COG0280C, COG0281C | malic enzyme |
| SMc01125 | **2.26** | *mutS* | COG0249L | DNA mismatch repair protein |
| SMc01124 | **6.03** | *glnD* | COG2844O | PII uridylyl-transferase |
| SMc01123 | **4.49/1.91** | *mviN* | COG0728R | putative virulence factor MviN-like protein |
| SMc01120 | **1.38** | *-* | *-* | hypothetical protein SMc01120 |
| SMc01119 | **2.20** | *-* | COG0694O | hypothetical protein SMc01119 |
| SMc01112 | **3.88** | *-* | COG1253R | hypothetical protein SMc01112 |
| SMc01109 | **2.04** | *metK* | COG0192H | S-adenosylmethionine synthetase |
| SMc01108 | **3.41** | *trmB* | COG0220R | tRNA (guanine-N(7))-methyltransferase |
| SMc01106 | **3.90** | *-* | COG0071O | small heat shock protein |
| SMc01102 | **1.95** | *-* | COG1322S | hypothetical protein SMc01102 |
| SMc01101 | **2.06** | *def* | COG0242J | peptide deformylase |
| SMc01097 | **2.89** | *-* | *-* | hypothetical protein SMc01097 |
| SMc01094 | **1.52** | *mexE1* | *-* | putative multidrug efflux system transmembrane protein |
| SMc01092 | **3.22/2.65** | *-* | COG0583K | putative transcription regulator protein |
| SMc01090 | **3.78** | *deaD* | COG0513LKJ | putative ATP-dependent RNA helicase protein |
| SMc01054 | **13.22** | *-* | *-* | hypothetical protein SMc01054 |
| SMc01053 | **13.88** | *cysG* | COG0007H, COG1648H | siroheme synthase protein |
| SMc01052 | **5.33** | *-* | COG0590FJ | hypothetical protein SMc01052 |
| SMc01049 | **2.19** | *hflX* | COG2262R | putative GTP-binding protein |
| SMc01048 | **1.43** | *hfq* | *-* | RNA-binding protein Hfq |
| SMc01047 | **2.07** | *-* | COG0115EH | D-amino acid aminotransferase |
| SMc01046 | **2.53** | *trkA* | COG0569P | potassium transporter peripheral membrane component |
| SMc01045 | **1.99** | *ntrX* | COG2204T | nitrogen regulation protein |
| SMc01044 | **1.51** | *ntrY* | COG5000T | nitrogen regulation transmembrane protein |
| SMc01037 | **3.00** | *lipA* | COG0320H | lipoyl synthase |
| SMc01036 | **4.84/3.40** | *-* | COG2261S | hypothetical protein SMc01036 |
| SMc01035 | **4.27/3.02** | *lpdA1* | COG1249C | dihydrolipoamide dehydrogenase |
| SMc01034 | **2.41** | *-* | COG0456R | hypothetical protein SMc01034 |
| SMc01033 | **2.04** | *-* | COG2755E | arylesterase protein |
| SMc01032 | **1.94** | *pdhB* | COG0508C | dihydrolipoamide S-acetyltransferase protein |
| SMc01031 | **3.35/2.08** | *pdhAb* | COG0022C, COG0508C | pyruvate dehydrogenase subunit beta |
| SMc01030 | **2.60/1.83** | *pdhAa* | COG1071C | pyruvate dehydrogenase alpha2 subunit protein |
| SMc01028 | **3.63/2.99** | *eno* | COG0148G | enolase protein |
| SMc01027 | **2.54** | *kdsA* | COG2877M | 2-dehydro-3-deoxyphosphooctonate aldolase |
| SMc01025 | **2.18** | *pyrG* | COG0504F | CTP synthetase |
| SMc01024 | **4.21** | *secG* | *-* | preprotein translocase subunit SecG |
| SMc01023 | **3.46** | *tpiA1* | COG0149G | triosephosphate isomerase protein |
| SMc01018 | **1.73** | *parE* | COG0187L | DNA topoisomerase IV subunit B |
| SMc01012 | **4.47/3.01** | *-* | COG0778C | hypothetical protein SMc01012 |
| SMc01011 | **3.40/3.07** | *-* | *-* | hypothetical protein SMc01011 |
| SMc01009 | **2.47** | *-* | COG3797S | hypothetical protein SMc01009 |
| SMc01007 | **2.35** | *-* | COG0759S | hypothetical protein SMc01007 |
| SMc01006 | **1.80** | *-* | COG0822C | hypothetical protein SMc01006 |
| SMc01005 | **3.36/2.22** | *folE* | COG0302H | GTP cyclohydrolase I |
| SMc01004 | **3.27** | *hisI* | COG0139E | phosphoribosyl-AMP cyclohydrolase |
| SMc01001 | **2.98** | *-* | COG0705R | hypothetical protein SMc01001 |
| SMc00995 | **2.67** | *-* | *-* | hypothetical protein SMc00995 |
| SMc00993 | **3.20** | *purD* | COG0151F | phosphoribosylamine--glycine ligase |
| SMc00990 | **1.62** | *Fsr* | COG0477GEPR | putative fosmidomycin resistance antibiotic resistance transmembrane protein |
| SMc00988 | **2.34** | *ubiA* | COG0382H | prenyltransferase |
| SMc00986 | **12.12** | *-* | *-* | hypothetical protein SMc00986 |
| SMc00975 | **2.31** | *mcpU* | *-* | chemoreceptor methyl-accepting chemotaxis transmembrane protein |
| SMc00973 | **2.10** | *-* | *-* | hypothetical protein SMc00973 |
| SMc00972 | **2.15** | *dxs* | COG1154HI | 1-deoxy-D-xylulose-5-phosphate synthase |
| SMc00968 | **2.49** | *-* | COG0604CR | putative oxidoreductase protein |
| SMc00966 | **2.24** | *-* | COG0183I | putative acetyl-COA acyltransferase protein |
| SMc00965 | **3.74** | *-* | COG0318IQ | hypothetical protein SMc00965 |
| SMc00964 | **3.07** | *bioB* | COG1268R | Putative biotin transporter |
| SMc00963 | **3.24** | *bioN* | COG0619P | Biotin ABC transporter, permease component |
| SMc00960 | **2.49** | *-* | COG0312R | hypothetical protein SMc00960 |
| SMc00950 | **4.61** | *-* | *-* | putative signal peptide protein |
| SMc00949 | **1.59** | *-* | COG3785S | hypothetical protein SMc00949 |
| SMc00943 | **2.04** | *wrbA1* | COG0655R | TrpR binding protein WrbA |
| SMc00937 | **1.98** | *trkH* | COG0168P | TRK system potassium uptake transmembrane protein |
| SMc00933 | **2.10** | *-* | *-* | hypothetical protein SMc00933 |
| SMc00925 | **1.87** | *-* | COG1376S | putative signal peptide protein |
| SMc00916 | **3.41** | *gst11* | COG0625O | putative glutathione S-transferase protein |
| SMc00914 | **2.93/2.75** | *-* | COG0446R | putative oxidoreductase protein |
| SMc00913 | **17.45** | *groEL1* | COG0459O | chaperonin GroEL |
| SMc00912 | **4.41** | *groES1* | COG0234O | co-chaperonin GroES |
| SMc00910 | **2.80** | *-* | COG0647G | hypothetical protein SMc00910 |
| SMc00907 | **2.73** | *-* | *-* | hypothetical protein SMc00907 |
| SMc00906 | **6.27** | *-* | COG0454KR | putative acetyltransferase protein |
| SMc00905 | **2.10/1.80** | *-* | COG0590FJ | putative deaminase protein |
| SMc00904 | **3.40** | *-* | COG1187J | hypothetical protein SMc00904 |
| SMc00902 | **5.10** | *-* | COG0742L | hypothetical protein SMc00902 |
| SMc00897 | **2.06** | *pmbA* | COG0312R | PMBA protein |
| SMc00896 | **2.46** | *-* | COG0483G | putative sulfite biosynthesis protein |
| SMc00895 | **2.95** | *-* | COG0743I | hypothetical protein SMc00895 |
| SMc00894 | **3.83** | *kdtA* | COG1519M | 3-deoxy-D-manno-octulosonic-acid transferase |
| SMc00893 | **2.47** | *-* | COG0546R | hypothetical protein SMc00893 |
| SMc00892 | **2.78** | *lpxK* | COG1663M | tetraacyldisaccharide 4'-kinase |
| SMc00888 | **1.80** | *-* | COG0784T | putative contains A 2-component receiver domain protein |
| SMc00876 | **1.84** | *-* | COG0489D | putative MRP protein homolog ATP-binding |
| SMc00874 | **3.42** | *corA2* | COG0598P | magnesium/cobalt transporter CorA |
| SMc00873 | **11.40** | *kup1* | COG3158P | KUP system potassium uptake transmembrane protein |
| SMc00872 | **5.27** | *atpI* | *-* | putative FOF1 ATP synthase, subunit I transmembrane protein |
| SMc00871 | **8.26/2.60** | *atpB* | COG0356C | F0F1 ATP synthase subunit A |
| SMc00870 | **6.42/4.44** | *atpE* | COG0636C | F0F1 ATP synthase subunit C |
| SMc00869 | **8.65** | *atpF2* | COG0711C | F0F1 ATP synthase subunit B' |
| SMc00868 | **8.11/7.98** | *atpF* | COG0711C | F0F1 ATP synthase subunit B |
| SMc00857 | **3.99** | *sohB* | COG0616OU | Probable proteinase |
| SMc00856 | **2.45** | *-* | *-* | hypothetical protein SMc00856 |
| SMc00854 | **5.69** | *-* | *-* | hypothetical protein SMc00854 |
| SMc00850 | **2.13** | *-* | COG1683S | hypothetical protein SMc00850 |
| SMc00830 | **2.31** | *-* | *-* | hypothetical protein SMc00830 |
| SMc00827 | **2.16** | *-* | *-* | putative transport transmembrane protein |
| SMc00825 | **4.10** | *gsh1* | *-* | putative glutamate--cysteine ligase precursor protein |
| SMc00819 | **3.71** | *katA* | COG0753P | catalase hydroperoxidase HPII(III) protein |
| SMc00817 | **1.92** | *-* | COG0412Q | putative carboxymethylenebutenolidase (dienelactone hydrolase) protein |
| SMc00815 | **7.44** | *guaB* | COG0516F, COG0517R | inositol-5-monophosphate dehydrogenase |
| SMc00812 | **2.53** | *-* | COG0346E | hypothetical protein SMc00812 |
| SMc00811 | **4.18** | *rrmJ* | COG0293J | Ribosomal RNA large subunit methyltransferase J |
| SMc00810 | **2.36** | *ppx2* | COG0248FP | Putative exopolyphosphatase |
| SMc00808 | **1.85** | *chrA* | COG2059P | chromate transporter |
| SMc00797 | **1.82** | *-* | COG0665E | putative oxidoreductase protein |
| SMc00791 | **1.67** | *-* | COG0251J | hypothetical protein SMc00791 |
| SMc00775 | **1.97** | *fbpB* | COG1178P | putative IRON(III) permease protein |
| SMc00761 | **1.95** | *ordL2* | COG0665E | putative oxidoreductase protein |
| SMc00741 | **2.05** | *-* | COG0365I | putative fatty-acid-CoA ligase protein |
| SMc00738 | **3.03** | *-* | COG1495O | hypothetical protein SMc00738 |
| SMc00730 | **1.98** | *-* | COG0705R | hypothetical protein SMc00730 |
| SMc00729 | **1.76** | *etfB1* | *-* | putative electron transfer flavoprotein BETA-subunit BETA-ETF flavoprotein small subunit |
| SMc00727 | **2.08** | *hbdA* | COG1250I | 3-hydroxybutyryl-CoA dehydrogenase |
| SMc00726 | **2.84/2.35** | *tlpA* | COG0526OC | putative thiol:disulfide interchange redox-active center transmembrane protein |
| SMc00723 | **4.00/3.41** | *lysA* | COG0019E | diaminopimelate DAP decarboxylase protein |
| SMc00715 | **2.31** | *-* | COG1434S | hypothetical protein SMc00715 |
| SMc00714 | **5.59** | *-* | COG0204I | putative 1-acyl-SN-glycerol-3-phosphate acyltransferase (PLSC) protein |
| SMc00710 | **4.12** | *hisC1* | COG0079E | histidinol-phosphate aminotransferase |
| SMc00704 | **6.00** | *rpmB* | COG0227J | 50S ribosomal protein L28 |
| SMc00703 | **6.17** | *-* | COG1738S | hypothetical protein SMc00703 |
| SMc00702 | **3.25/2.31** | *-* | COG1235R | putative signal peptide protein |
| SMc00701 | **3.40/2.78** | *cobT* | COG4547H | cobalamin biosynthesis protein |
| SMc00700 | **2.19** | *cobS* | COG0714R | cobalamin biosynthesis protein |
| SMc00699 | **2.82** | *-* | COG2214O | hypothetical protein SMc00699 |
| SMc00698 | **1.53** | *-* | COG0271T | putative transcription regulator protein |
| SMc00697 | **2.57** | *-* | COG4536P | hypothetical protein SMc00697 |
| SMc00696 | **1.71** | *aroB* | COG0337E | 3-dehydroquinate synthase |
| SMc00695 | **1.72** | *aroK* | COG0703E | putative shikimate kinase I protein |
| SMc00691 | **2.82** | *xerD* | COG4974L | site-specific tyrosine recombinase XerD |
| SMc00690 | **3.42** | *accA* | COG0825I | acetyl-CoA carboxylase carboxyltransferase subunit alpha |
| SMc00687 | **2.11** | *-* | COG1487R | hypothetical protein SMc00687 |
| SMc00682 | **1.46** | *hipO1* | COG1473R | putative hippurate hydrolase protein |
| SMc00681 | **1.53** | *lrp* | COG1522K | leucine-responsive regulatory protein |
| SMc00647 | **2.32** | *rluD* | COG0564J | putative ribosomal large subunit pseudouridine synthase protein |
| SMc00646 | **3.87/3.36** | *rpoH1* | COG0568K | RNA polymerase factor sigma-32 |
| SMc00644 | **2.01** | *-* | *-* | hypothetical protein SMc00644 |
| SMc00643 | **3.61** | *purA* | COG0104F | adenylosuccinate synthetase |
| SMc00642 | **2.19/1.85** | *-* | COG0697GER | hypothetical protein SMc00642 |
| SMc00641 | **1.36** | *serA* | COG0111HE | D-3-phosphoglycerate dehydrogenase |
| SMc00622 | **2.04** | *rnd1* | COG0349J | ribonuclease D protein |
| SMc00620 | **2.49** | *-* | COG4222S | putative signal peptide protein |
| SMc00619 | **2.79** | *ppx1* | COG0248FP | Putative exopolyphosphatase |
| SMc00616 | **2.50** | *perM* | COG0628R | putative permease protein |
| SMc00615 | **2.98** | *purM* | COG0150F | phosphoribosylaminoimidazole synthetase |
| SMc00614 | **3.21** | *purN* | COG0299F | phosphoribosylglycinamide formyltransferase |
| SMc00605 | **1.84** | *-* | COG0625O | hypothetical protein SMc00605 |
| SMc00602 | **2.61** | *uvrC* | COG0322L | excinuclease ABC subunit C |
| SMc00601 | **1.60** | *pgsA* | COG0314H | CDP-diacylglycerol--glycerol-3-phosphate 3-phosphatidyltransferase transmembrane protein |
| SMc00599 | **1.73** | *moaE* | COG0314H | molybdopterin MPT converting factor subunit 2 |
| SMc00595 | **8.61** | *ndk* | COG0105F | nucleoside diphosphate kinase |
| SMc00594 | **2.55** | *ligE* | COG0625O | putative BETA-etherase (BETA-aryl ether cleaving enzyme) protein |
| SMc00592 | **4.69** | *-* | COG0318IQ, COG1020Q | hypothetical protein SMc00592 |
| SMc00591 | **3.07** | *-* | *-* | hypothetical protein SMc00591 |
| SMc00590 | **3.91** | *-* | COG0488R | putative ABC transporter ATP-binding protein |
| SMc00586 | **3.00** | *-* | COG2927L | DNA polymerase III subunit chi |
| SMc00585 | **2.20** | *pepA* | COG0260E | leucyl aminopeptidase |
| SMc00583 | **1.98** | *-* | COG0795R | hypothetical protein SMc00583 |
| SMc00582 | **1.80** | *-* | COG1452M | hypothetical protein SMc00582 |
| SMc00581 | **1.69** | *-* | COG0760O | hypothetical protein SMc00581 |
| SMc00580 | **3.62** | *pdxA* | COG1995H | 4-hydroxythreonine-4-phosphate dehydrogenase |
| SMc00579 | **3.41** | *ksgA* | COG0030J | dimethyladenosine transferase |
| SMc00578 | **2.24** | *-* | *-* | hypothetical protein SMc00578 |
| SMc00575 | **2.97** | *-* | COG1559R | hypothetical protein SMc00575 |
| SMc00574 | **3.04/2.29** | *fabF* | COG0304IQ | 3-oxoacyl-(acyl carrier protein) synthase II |
| SMc00573 | **2.40** | *acpP* | COG0236IQ | acyl carrier protein |
| SMc00572 | **3.07** | *fabG* | COG1028IQR | 3-ketoacyl-(acyl-carrier-protein) reductase NodG |
| SMc00571 | **2.92** | *fabD* | COG0331I | acyl-carrier-protein S-malonyltransferase |
| SMc00570 | **4.79** | *-* | COG0667C | putative oxidoreductase protein |
| SMc00568 | **25.85/7.63** | *rpsF* | COG0360J | 30S ribosomal protein S6 |
| SMc00567 | **21.87/4.96** | *rpsR* | COG0238J | 30S ribosomal protein S18 |
| SMc00566 | **3.76** | *-* | *-* | hypothetical protein SMc00566 |
| SMc00565 | **13.44/2.38** | *rplI* | COG0359J | 50S ribosomal protein L9 |
| SMc00561 | **2.24** | *dnaB* | COG0305L | replicative DNA helicase |
| SMc00556 | **2.34** | *radA* | COG1066O | DNA repair protein RadA |
| SMc00554 | **2.05** | *purF* | COG0034F | amidophosphoribosyltransferase |
| SMc00553 | **3.07** | *-* | COG1028IQR | putative oxidoreductase protein |
| SMc00552 | **1.89** | *pssA* | COG1183I | putative CDP-diacylglycerol--serine O-phosphatidyltransferase transmembrane protein |
| SMc00551 | **2.12** | *-* | COG0688I | phosphatidylserine decarboxylase |
| SMc00550 | **5.43** | *-* | COG5265O | ABC transporter ATP-binding protein |
| SMc00537 | **1.89** | *-* | COG0477GEPR | putative transport protein |
| SMc00536 | **3.45** | *-* | *-* | putative transport protein |
| SMc00534 | **2.48** | *ttcA* | COG0037D | Putative tRNA 2-thiocytidine biosynthesis protein |
| SMc00533 | **2.24** | *-* | COG0520E | putative pyridoxal-phosphate-dependent aminotransferase protein |
| SMc00532 | **4.79/2.25** | *-* | COG0719O | hypothetical protein SMc00532 |
| SMc00531 | **2.59** | *-* | COG0396O | putative ABC transporter ATP-binding protein |
| SMc00530 | **4.56/3.00** | *-* | COG0719O | cysteine desulfurase activator complex subunit SufB |
| SMc00528 | **3.02/2.95** | *-* | *-* | hypothetical protein SMc00528 |
| SMc00525 | **2.51** | *-* | *-* | hypothetical protein SMc00525 |
| SMc00522 | **8.53** | *rhlE1* | COG0513LKJ | putative ATP-dependent RNA helicase protein |
| SMc00521 | **13.50** | *-* | COG1051F | hypothetical protein SMc00521 |
| SMc00514 | **2.16** | *-* | COG1853R | putative monooxygenase protein |
| SMc00511 | **2.46/2.19** | *rpe* | COG0036G | D-ribulose-5-phosphate 3-epimerase protein |
| SMc00508 | **4.29** | *purB* | COG0015F | adenylosuccinate lyase |
| SMc00495 | **2.18** | *purC* | COG0152F | phosphoribosylaminoimidazole-succinocarboxamide synthase |
| SMc00494 | **2.70** | *purS* | COG1828F | phosphoribosylformylglycinamidine synthase subunit PurS |
| SMc00493 | **3.85** | *purQ* | COG0047F | phosphoribosylformylglycinamidine synthase subunit I |
| SMc00492 | **2.06** | *-* | COG4992E | hypothetical protein SMc00492 |
| SMc00488 | **2.93/2.46** | *purL* | COG0046F | phosphoribosylformylglycinamidine synthase II |
| SMc00486 | **2.59** | *glsA* | COG2066E | glutaminase |
| SMc00485 | **9.20/8.80** | *rpsD* | COG0522J | 30S ribosomal protein S4 |
| SMc00482 | **2.53** | *-* | COG0565J | hypothetical protein SMc00482 |
| SMc00480 | **4.43** | *icd* | COG0538C | isocitrate dehydrogenase |
| SMc00472 | **2.84/2.61** | *-* | COG2313Q | hypothetical protein SMc00472 |
| SMc00469 | **3.07** | *dksA* | *-* | putative DNAK SUPPRESOR protein |
| SMc00467 | **3.49** | *-* | COG3106R | hypothetical protein SMc00467 |
| SMc00463 | **1.49** | *folB* | COG1539H | dihydroneopterin aldolase DHNA lyase folate biosynthesis protein |
| SMc00450 | **4.00** | *ctaB* | COG0109O | protoheme IX farnesyltransferase |
| SMc00427 | **3.50** | *prfC* | COG4108J | peptide chain release factor RF-3 protein |
| SMc00423 | **2.06** | *-* | COG1280E | putative amino acid efflux protein |
| SMc00421 | **1.90** | *cysK1* | COG0031E | O-acetylserine sulfhydrylase A |
| SMc00419 | **2.14/1.81** | *gshB1* | COG0189HJ | glutathione synthetase |
| SMc00415 | **1.38** | *dnaN* | COG0592L | DNA polymerase III subunit beta |
| SMc00412 | **2.22** | *pyrF* | COG0284F | orotidine 5'-phosphate decarboxylase |
| SMc00411 | **1.83** | *-* | COG5470S | hypothetical protein SMc00411 |
| SMc00410 | **2.19** | *-* | COG0702MG | putative oxidoreductase protein |
| SMc00409 | **4.69** | *-* | *-* | putative signal peptide protein |
| SMc00408 | **5.15/1.69** | *uppP* | COG1968V | undecaprenyl pyrophosphate phosphatase |
| SMc00407 | **2.37** | *gst4* | COG0625O | putative glutathione S-transferase protein |
| SMc00405 | **2.05** | *-* | COG0451MG | hypothetical protein SMc00405 |
| SMc00403 | **1.90** | *-* | COG0144J | NOL1/NOP2/SUN family signature protein |
| SMc00402 | **1.56** | *-* | COG3487P | putative signal peptide protein |
| SMc00401 | **3.57** | *-* | *-* | hypothetical protein SMc00401 |
| SMc00400 | **3.09** | *-* | COG3489R | putative signal peptide protein |
| SMc00399 | **2.77** | *corA1* | COG0598P | magnesium/cobalt transporter CorA |
| SMc00394 | **5.17** | *guaA* | COG0518F, COG0519F | bifunctional GMP synthase/glutamine amidotransferase protein |
| SMc00389 | **2.53** | *-* | *-* | hypothetical protein SMc00389 |
| SMc00388 | **2.14** | *-* | COG3608R | hypothetical protein SMc00388 |
| SMc00369 | **1.76** | *-* | COG0667C | putative oxidoreductase protein |
| SMc00364 | **3.63** | *rplT* | COG0292J | 50S ribosomal protein L20 |
| SMc00363 | **3.76** | *rpmI* | COG0291J | 50S ribosomal protein L35 |
| SMc00362 | **2.83/1.57** | *infC* | COG0290J | translation initiation factor IF-3 |
| SMc00357 | **6.06** | *efp* | COG0231J | elongation factor P |
| SMc00349 | **4.23** | *lepA* | COG0481M | GTP-binding protein LepA |
| SMc00346 | **2.24** | *-* | COG2259S | hypothetical protein SMc00346 |
| SMc00341 | **1.85** | *-* | *-* | hypothetical protein SMc00341 |
| SMc00339 | **2.06** | *cyaA* | COG0840NT, COG2114T | adenylate cyclase 1 protein |
| SMc00335 | **10.24/6.84** | *rpsA* | COG0539J | 30S ribosomal protein S1 |
| SMc00334 | **4.83** | *cmk* | COG0283F | cytidylate kinase |
| SMc00333 | **6.13** | *aroA* | COG0128E | 3-phosphoshikimate 1-carboxyvinyltransferase |
| SMc00332 | **6.64** | *-* | *-* | hypothetical protein SMc00332 |
| SMc00329 | **2.56/1.37** | *irr* | COG0735P | putative IRON response regulator protein |
| SMc00328 | **1.87** | *fabA* | COG0764I | 3-hydroxydecanoyl-(acyl carrier protein) dehydratase |
| SMc00327 | **2.09** | *fabB* | COG0304IQ | 3-oxoacyl-(acyl carrier protein) synthase I |
| SMc00326 | **2.49** | *fabI2* | COG0623I | enoyl-(acyl carrier protein) reductase |
| SMc00325 | **3.45** | *-* | COG2813J | hypothetical protein SMc00325 |
| SMc00324 | **10.11/5.96** | *pnp* | COG1185J | polynucleotide phosphorylase/polyadenylase |
| SMc00323 | **8.72** | *rpsO* | COG0184J | 30S ribosomal protein S15 |
| SMc00320 | **8.85** | *rbfA* | COG0858J | ribosome-binding factor A |
| SMc00317 | **3.21** | *-* | COG0679R | hypothetical protein SMc00317 |
| SMc00302 | **4.70** | *-* | COG2151R | hypothetical protein SMc00302 |
| SMc00301 | **1.85** | *-* | COG0316S | hypothetical protein SMc00301 |
| SMc00294 | **2.08** | *-* | COG0436E | aminotransferase |
| SMc00293 | **3.13/2.45** | *thrA* | COG0460E | homoserine dehydrogenase |
| SMc00292 | **2.78** | *recJ* | COG0608L | single-stranded-DNA-specific exonuclease protein |
| SMc00291 | **2.09** | *-* | COG3108S | lipoprotein |
| SMc00287 | **4.59/3.44** | *-* | *-* | putative oxidoreductase protein |
| SMc00286 | **1.79** | *-* | COG2931Q | hemolysin-type calcium-binding protein |
| SMc00282 | **6.75** | *-* | COG3803S | hypothetical protein SMc00282 |
| SMc00265 | **4.34** | *-* | COG1638G | putative periplasmic binding protein |
| SMc00261 | **3.25** | *-* | COG0318IQ | acyl-CoA synthetase |
| SMc00255 | **1.82** | *-* | *-* | hypothetical protein SMc00255 |
| SMc00248 | **3.59** | *ccsA* | COG0785O | putative cytochrome C-type biogenesis protein |
| SMc00247 | **1.83** | *pcs* | COG1183I | phosphatidylcholine synthase protein |
| SMc00240 | **2.76** | *-* | COG1253R | hypothetical protein SMc00240 |
| SMc00238 | **1.96** | *moeA* | COG0303H | molybdopterin biosynthesis protein |
| SMc00234 | **2.03** | *ppiD* | COG0760O | putative peptidyl-prolyl cis-trans isomerase protein |
| SMc00227 | **4.48** | *-* | COG2938S | hypothetical protein SMc00227 |
| SMc00198 | **1.60** | *-* | *-* | hypothetical protein SMc00198 |
| SMc00193 | **2.67** | *-* | *-* | hypothetical protein SMc00193 |
| SMc00189 | **2.72** | *fbcC* | COG2857C | putative cytochrome C1 protein |
| SMc00188 | **3.36/2.72** | *fbcB* | COG1290C | cytochrome B transmembrane protein |
| SMc00187 | **2.70/2.32** | *fbcF* | COG0723C | ubiquinol-cytochrome C reductase IRON-sulfur subunit protein |
| SMc00186 | **2.21** | *-* | COG1132V | ABC transporter ATP-binding transmembrane protein |
| SMc00176 | **1.75** | *-* | COG1463Q | hypothetical protein SMc00176 |
| SMc00175 | **1.61** | *-* | COG1127Q | putative ABC transporter ATP-binding protein |
| SMc00171 | **2.04** | *-* | COG2908S | hypothetical protein SMc00171 |
| SMc00169 | **1.97** | *dme* | COG0280C, COG0281C | malic enzyme |
| SMc00156 | **2.12** | *-* | COG3791S | hypothetical protein SMc00156 |
| SMc00155 | **4.88** | *aroF* | COG3200E | DAHP synthetase prtein |
| SMc00154 | **1.84** | *gor* | COG1249C | glutathione reductase |
| SMc00153 | **4.80/3.39** | *-* | COG3184S | hypothetical protein SMc00153 |
| SMc00152 | **3.69** | *rpiA* | COG0120G | ribose-5-phosphate isomerase A |
| SMc00151 | **3.39** | *gph1* | COG0546R | phosphoglycolate phosphatase |
| SMc00149 | **2.87** | *fumC* | *-* | fumarate hydratase |
| SMc00134 | **2.02** | *-* | COG0457R, COG0500QR | hypothetical protein SMc00134 |
| SMc00130 | **2.33** | *-* | *-* | hypothetical protein SMc00130 |
| SMc00127 | **2.46** | *betC* | COG3119P | choline sulfatase protein |
| SMc00118 | **2.91** | *-* | COG2166R | hypothetical protein SMc00118 |
| SMc00117 | **2.35** | *-* | *-* | hypothetical protein SMc00117 |
| SMc00114 | **3.06** | *ptrB* | COG1770E | protease II oligopeptidase B hydrolase serine protease protein |
| SMc00109 | **1.90** | *-* | COG0789K | putative transcription regulator protein |
| SMc00108 | **2.26** | *-* | COG0454KR | putative acetyltransferase protein |
| SMc00095 | **2.16** | *betI* | COG1309K | transcriptional regulator BetI |
| SMc00092 | **13.39** | *cysH* | COG0175EH | phosphoadenosine phosphosulfate reductase PAPS reductase |
| SMc00091 | **21.08** | *cysD* | COG0175EH | sulfate adenylyltransferase subunit 2 |
| SMc00090 | **37.50** | *cysN* | COG2895P | putative sulfate adenylate transferase cysteine biosynthesis protein |
| SMc00088 | **2.98** | *-* | *-* | hypothetical protein SMc00088 |
| SMc00086 | **1.35** | *cycG* | COG2010C | putative diheme cytochrome C-type signal peptide protein |
| SMc00085 | **3.17** | *-* | COG3346S | putative cytochrome oxidase complex biogenesis factor transmembrane protein |
| SMc00079 | **4.06** | *-* | COG0637R | hypothetical protein SMc00079 |
| SMc00078 | **1.60** | *livJ* | COG0683E | Leu/Ile/Val-binding protein |
| SMc00077 | **3.83** | *thrC1* | COG0498E | threonine synthase |
| SMc00074 | **1.99** | *-* | COG2199T, COG2200T | putative transmembrane signal peptide protein |
| SMc00067 | **2.07** | *lppA* | COG4520M | outer-membranne lipoprotein |
| SMc00062 | **2.40** | *-* | COG4991S | hypothetical protein SMc00062 |
| SMc00061 | **2.16** | *-* | *-* | hypothetical protein SMc00061 |
| SMc00058 | **1.40** | *mucR* | *-* | transcription regulator protein |
| SMc00057 | **2.56** | *phaG2* | COG1320P | putative monovalent cation/H+ antiporter subunit G |
| SMc00055 | **2.43** | *phaE2* | COG1863P | putative monovalent cation/H+ antiporter subunit E |
| SMc00054 | **3.09** | *phaD2* | COG0651CP | putative monovalent cation/H+ antiporter subunit D |
| SMc00053 | **2.19** | *phaC2* | COG1006P | putative monovalent cation/H+ antiporter subunit C |
| SMc00052 | **2.30** | *phaB2* | COG2111P | putative monovalent cation/H+ antiporter subunit B |
| SMc00051 | **2.53** | *phaA2* | COG1009CP, COG2111P | putative monovalent cation/H+ antiporter subunit A |
| SMc00043 | **2.36/2.09** | *sodB* | COG0605P | superoxide dismutase Fe protein |
| SMc00039 | **1.53** | *-* | COG1376S | hypothetical protein SMc00039 |
| SMc00036 | **1.88** | *gst1* | COG0625O | putative glutathione S-transferase protein |
| SMc00033 | **2.45** | *-* | COG2199T, COG2200T | hypothetical protein SMc00033 |
| SMc00024 | **1.90** | *smc* | COG1196D | putative chromosome partition protein |
| SMc00023 | **2.52** | *-* | COG1651O | hypothetical protein SMc00023 |
| SMc00020 | **2.92/2.71** | *-* | *-* | hypothetical protein SMc00020 |
| SMc00018 | **2.87** | *rnhA* | COG0328L | ribonuclease H |
| SMc00017 | **4.59** | *thrB* | COG2334R | homoserine kinase |
| SMc00016 | **3.29** | *ispH* | COG0761IM | 4-hydroxy-3-methylbut-2-enyl diphosphate reductase |
| SMc00014 | **2.13** | *-* | *-* | hypothetical protein SMc00014 |
| SMc00013 | **2.46** | *ctaE* | *-* | putative cytochrome C oxidase subunit III transmembrane protein |
| SMc00012 | **2.96** | *ctaG* | COG3175O | cytochrome C oxidase assembly protein |
| SMc00010 | **2.65** | *ctaD* | COG0843C | putative cytochrome C oxidase polypeptide I transmembrane protein |
| SMc00009 | **2.96** | *ctaC* | COG1622C | putative cytochrome C oxidase subunit II protein |
| SMc00008 | **4.54** | *ribA* | COG0108H, COG0807H | riboflavin biosynthesis protein RibA |
| SMc00007 | **4.71** | *aroC* | COG0082E | chorismate synthase |
| SMc00005 | **2.38** | *fabI1* | COG0623I | enoyl-(acyl carrier protein) reductase |
| SMb21691 | **3.70** | *-* | COG2141C | putative nitrilotriacetate monooxygenase component A protein |
| SMb21676 | **2.70** | *-* | *-* | hypothetical protein SM_b21676 |
| SMb21651 | **1.87** | *-* | COG1487R | putative plasmid stability protein |
| SMb21631 | **3.29** | *nspC* | COG0019E | putative carboxynorspermidine decarboxylase protein |
| SMb21630 | **3.12** | *-* | COG1748E | hypothetical protein SM_b21630 |
| SMb21605 | **2.30** | *-* | COG3839G | putative sugar uptake ABC transporter ATP-binding protein |
| SMb21602 | **3.34** | *-* | COG0395G | putative sugar uptake ABC transporter permease protein |
| SMb21568 | **3.83** | *-* | *-* | hypothetical protein SM_b21568 |
| SMb21566 | **6.59** | *groEL* | COG0459O | chaperonin GroEL |
| SMb21562 | **69.63** | *-* | COG3678UNTP | hypothetical protein SM_b21562 |
| SMb21561 | **27.13/7.39** | *-* | COG0745TK | putative two component response regulator protein |
| SMb21560 | **14.72** | *-* | COG0642T | putative two component sensor histidine kinase protein |
| SMb21559 | **2.65** | *-* | COG2207K | putative transcriptional regulator, araC family protein |
| SMb21552 | **4.50** | *aacC4* | COG1670J | putative aminoglycoside 6'-N-acetyltransferase, amikacin resistance protein |
| SMb21551 | **6.32/3.95** | *-* | *-* | hypothetical protein SM_b21551 |
| SMb21550 | **10.25** | *-* | COG2391R | hypothetical protein SM_b21550 |
| SMb21549 | **29.33** | *thtR* | COG2897P | putative exported sulfurtransferase, rhodanese protein |
| SMb21508 | **3.11** | *dapA3* | COG0329EM | putative dihydrodipicolinate synthetase protein |
| SMb21492 | **2.13** | *-* | *-* | hypothetical protein SM_b21492 |
| SMb21491 | **1.83** | *-* | *-* | hypothetical protein SM_b21491 |
| SMb21490 | **5.72** | *-* | COG3346S | hypothetical protein SM_b21490 |
| SMb21489 | **13.58** | *cyoC* | COG1845C | putative cytochrome o ubiquinol oxidase chain III protein |
| SMb21488 | **22.22** | *cyoB* | COG0843C | putative cytochrome o ubiquinol oxidase chain I protein |
| SMb21487 | **14.24** | *cyoA* | COG1622C | putative cytochrome o ubiquinol oxidase chain II protein |
| SMb21440 | **1.67** | *-* | *-* | hypothetical protein SM_b21440 |
| SMb21432 | **4.33** | *-* | COG0614P | putative iron uptake ABC transporter periplasmic solute-binding protein precursor |
| SMb21431 | **5.21/4.10** | *-* | COG0614P | hypothetical protein SM_b21431 |
| SMb21430 | **3.72** | *-* | COG0609P | putative iron ABC transporter permease protein |
| SMb21429 | **4.09** | *-* | COG1120PH | putative iron ABC transporter ATP-binding protein |
| SMb21425 | **2.89** | *-* | COG3510V | hypothetical protein SM_b21425 |
| SMb21416 | **5.01** | *ddhA* | COG1208MJ | putative glucose-1-phosphate cytidylyltransferase protein |
| SMb21411 | **5.29** | *-* | *-* | hypothetical protein SM_b21411 |
| SMb21379 | **2.46** | *-* | COG2343S | hypothetical protein SM_b21379 |
| SMb21346 | **1.79** | *-* | COG1609K | putative transcriptional regulator, LacI family protein |
| SMb21345 | **5.41** | *-* | COG1879G | putative sugar uptake ABC transporter periplasmic solute-binding protein precursor |
| SMb21328 | **3.81** | *-* | COG0846K | putative Sir2-like transcriptional silencer protein |
| SMb21296 | **1.66** | *-* | *-* | hypothetical protein SM_b21296 |
| SMb21295 | **2.79** | *-* | COG0071O | Hsp20 family heat-shock protein |
| SMb21274 | **4.97** | *potB* | COG1176E | putative spermidineputrescine ABC transporter permeasee protein |
| SMb21273 | **4.22** | *potD* | COG0687E | putative spermidineputrescine ABC transporter periplasmic solute-binding protein precursor |
| SMb21271 | **3.72** | *-* | COG0500QR | nucleotide-binding protein |
| SMb21269 | **2.69** | *-* | COG1132V | ABC transporter ATPase |
| SMb21268 | **1.80** | *-* | *-* | hypothetical protein SM_b21268 |
| SMb21266 | **4.28** | *-* | COG4671R | hypothetical protein SM_b21266 |
| SMb21265 | **3.44** | *redB* | COG0438M | putative glycosyltransferase protein |
| SMb21264 | **1.81** | *redA* | COG4671R | putative membrane-anchored protein |
| SMb21258 | **4.30** | *-* | COG2114T | adenylate cyclase |
| SMb21257 | **7.63** | *-* | COG2114T | adenylate cyclase |
| SMb21256 | **1.96** | *-* | COG0677M | putative nucleotide sugar oxidase protein |
| SMb21252 | **3.18** | *-* | COG0438M | putative glycosyltransferase protein |
| SMb21213 | **2.34** | *pphA* | COG0639T | putative serinethreonine protein phosphatase |
| SMb21209 | **2.42** | *-* | COG0642T | putative two-component sensor histidine kinase protein |
| SMb21208 | **2.69** | *-* | *-* | putative transcriptional regulator, TetR family protein |
| SMb21207 | **3.28** | *-* | COG0845M | hypothetical protein SM_b21207 |
| SMb21206 | **3.16** | *-* | COG1131V | ABC transporter ATP-binding protein |
| SMb21189 | **2.83** | *-* | COG0463M | putative glycosyltransferase protein |
| SMb21186 | **6.22** | *gabT* | COG0160E | 4-aminobutyrate aminotransferase |
| SMb21185 | **2.72** | *gabD2* | COG1012C | putative succinate-semialdehyde dehydrogenase (NAD(P)+) protein |
| SMb21183 | **5.28** | *htpG* | COG0326O | heat shock protein 90 |
| SMb21177 | **1.83** | *phoC* | COG3638P | phosphate uptake ABC transporter ATP-binding protein |
| SMb21176 | **2.49** | *phoD* | COG3221P | phosphate uptake ABC transporter periplasmic solute-binding protein precursor |
| SMb21175 | **5.40** | *phoE* | COG3639P | phosphate uptake ABC transporter permease protein |
| SMb21174 | **5.29** | *phoT* | COG3639P | phosphate uptake ABC transporter permease protein |
| SMb21171 | **2.02** | *phnM* | COG3454P | putative enzyme protein |
| SMb21140 | **2.36** | *-* | COG1414K | putative transcriptional regulator protein |
| SMb21139 | **2.38** | *-* | COG0251J | translation initiation inhibitors |
| SMb21136 | **6.48** | *-* | COG0765E | putative amino acid uptake ABC transporter permease protein |
| SMb21133 | **1.77** | *-* | COG1613P | putative sulfate uptake ABC transporter periplasmic solute-binding protein precursor |
| SMb21132 | **1.61** | *-* | COG0555O | putative sulfate uptake ABC transporter permease protein |
| SMb21131 | **1.74** | *-* | COG4208P | putative sulfate uptake ABC transporter permease protein |
| SMb21130 | **1.58** | *-* | COG1118P | putative sulfate uptake ABC transporter ATP-binding protein |
| SMb21129 | **2.58** | *-* | COG0277C | putative oxidoreductase protein |
| SMb21113 | **1.89** | *-* | *-* | hypothetical protein SM_b21113 |
| SMb21060 | **3.02** | *-* | COG0451MG | putative sugar nucleotide epimerase dehydratase protein |
| SMb21009 | **5.49** | *glpK* | COG0554C | glycerol kinase, sugar kinase protein |
| SMb20999 | **1.62** | *bacA* | COG1133I | transport protein |
| SMb20996 | **2.82** | *-* | *-* | hypothetical protein SM_b20996 |
| SMb20995 | **3.16** | *engA* | COG1160R | GTP-binding protein EngA |
| SMb20948 | **1.88** | *exoU* | COG0463M | glucosyltransferase protein |
| SMb20940 | **3.96** | *exsB* | COG0603R | hypothetical protein SM_b20940 |
| SMb20939 | **3.98** | *exsC* | COG0720H | putative 6-pyruvoyl tetrahydropterin synthase protein |
| SMb20938 | **5.41** | *exsD* | COG0602O | hypothetical protein SM_b20938 |
| SMb20924 | **1.82** | *abfA* | COG3534G | putative alpha-L-arabinofuranosidase protein |
| SMb20906 | **4.53** | *-* | *-* | hypothetical protein SM_b20906 |
| SMb20904 | **1.15** | *-* | COG1129G | putative sugar uptake ABC transporter ATP-binding protein |
| SMb20898 | **2.04** | *-* | *-* | hypothetical protein SM_b20898 |
| SMb20885 | **3.49** | *-* | COG0697GER | hypothetical protein SM_b20885 |
| SMb20880 | **2.78** | *rhlE2* | COG0513LKJ | putative ATP-dependent RNA helicase protein |
| SMb20877 | **3.35** | *-* | COG0665E | oxidoreductase |
| SMb20873 | **2.57** | *allA* | COG3194F | ureidoglycolate hydrolase |
| SMb20859 | **3.44** | *-* | COG2207K | putative transcriptional regulator, araC family protein |
| SMb20838 | **2.15/1.88** | *-* | COG2931Q | putative secreted calcium-binding protein |
| SMb20833 | **2.27** | *rkpT1* | *-* | putative cell surface polysaccharide export ABC-2 transporter permease protein, close relative of Y20822wzm2 |
| SMb20827 | **1.90** | *-* | COG1682GM | transposase |
| SMb20825 | **3.19** | *-* | COG2801L | putative acetyltransferase, cysElacA/lpxA/nodL family protein |
| SMb20824 | **5.35** | *-* | COG0110R | putative membrane protein, slightly carrier proteins |
| SMb20823 | **1.87** | *rkpZ2* | COG0463M, COG3562M, COG4092M | cell surface saccharide synthesis protein |
| SMb20813 | **2.85** | *msbA1* | COG3563M | lipid ABC transporter permease/ATP-binding protein |
| SMb20810 | **2.23** | *-* | *-* | putative membrane-located cell surface saccharide saccharide acetylase protein |
| SMb20807 | **2.81** | *-* | COG1835I | hypothetical protein SM_b20807 |
| SMb20775 | **2.28** | *-* | *-* | TonB-dependent receptor protein |
| SMb20762 | **1.91** | *phnJ* | COG0457R | putative C-P (carbon-phosphorus) lyase component protein |
| SMb20760 | **2.20** | *phnH* | COG3627P | carbon-phosphorus lyase complex subunit |
| SMb20759 | **2.77/2.18** | *phnG* | COG3625P | putative C-P (carbon-phosphorus lyase component protein |
| SMb20758 | **2.11** | *-* | *-* | putative transcriptional regulator, GntR or ArsR family protein |
| SMb20749 | **1.40** | *uxuB* | COG0246G | putative D-mannonate oxidoreductase protein |
| SMb20746 | **4.47** | *gstI* | *-* | Glutamine synthetase translation inhibitor |
| SMb20727 | **1.49** | *-* | *-* | hypothetical protein SM_b20727 |
| SMb20725 | **2.85** | *-* | *-* | hypothetical protein SM_b20725 |
| SMb20724 | **3.08** | *-* | COG3181S | hypothetical protein SM_b20724 |
| SMb20708 | **2.29** | *-* | COG0350L | putative methylated-DNA--protein-cysteine methyltransferase |
| SMb20707 | **1.95** | *cyaG2* | COG2114T | putative adenylate cyclase protein |
| SMb20697 | **1.65** | *-* | COG4187E | putative peptidase, arginine utilization protein RocB |
| SMb20689 | **2.18** | *xthA4* | COG0708L | exodeoxyribonuclease III protein |
| SMb20676 | **2.10** | *-* | *-* | hypothetical protein SM_b20676 |
| SMb20614 | **2.55** | *-* | *-* | hypothetical protein SM_b20614 |
| SMb20573 | **6.66** | *-* | COG0431R | putative NADH-dependent FMN reductase protein |
| SMb20571 | **2.04** | *-* | COG0600P | putative aliphatic sulfonate uptake ABC transporter permease protein |
| SMb20570 | **3.22** | *-* | COG0715P | putative aliphatic sulfonates uptake ABC transporter periplasmic solute-binding protein precursor |
| SMb20569 | **3.33** | *-* | COG1116P | putative ABC transporter ATP-binding protein |
| SMb20561 | **1.94** | *-* | *-* | hypothetical protein SM_b20561 |
| SMb20551 | **2.31** | *-* | *-* | hypothetical protein SM_b20551 |
| SMb20509 | **3.30** | *-* | COG2186K | putative transcriptional regulator protein |
| SMb20495 | **9.67** | *-* | COG0457R | hypothetical protein SM_b20495 |
| SMb20490 | **2.09** | *fucA2* | COG0235G | putative L-fuculose phosphate aldolase protein |
| SMb20488 | **2.78** | *-* | COG1082G | hypothetical protein SM_b20488 |
| SMb20484 | **1.93** | *-* | COG1879G | putative ABC transporter periplasmic sugar-binding protein |
| SMb20370 | **2.56** | *-* | COG1136V | putative ATP-binding transport protein |
| SMb20369 | **3.06** | *-* | COG4591M | hypothetical protein SM_b20369 |
| SMb20368 | **2.82** | *-* | COG0845M | hypothetical protein SM_b20368 |
| SMb20361 | **2.53** | *-* | COG1226P | putative ionic voltage-gated channel protein |
| SMb20347 | **5.99/3.18** | *-* | COG1309K | putative transcriptional regulator protein |
| SMb20346 | **8.26/5.63** | *-* | COG0845M | putative efflux protein |
| SMb20345 | **7.64** | *-* | COG0841V | putative efflux protein |
| SMb20344 | **6.32** | *-* | COG2207K | putative transcriptional regulator protein |
| SMb20335 | **1.52** | *-* | *-* | hypothetical protein SM_b20335 |
| SMb20323 | **2.86** | *-* | COG1802K | putative transcriptional regulator protein |
| SMb20303 | **1.93** | *-* | *-* | hypothetical protein SM_b20303 |
| SMb20285 | **1.98** | *-* | COG0583K | putative transcriptional regulator protein |
| SMb20263 | **2.04** | *-* | COG0834ET | putative ABC transporter periplasmic amino acid-binding protein |
| SMb20262 | **1.93** | *-* | COG1012C | putative semialdehyde dehydrogenase protein |
| SMb20201 | **3.60** | *cbbP* | COG3954C | putative phosphoribulokinase protein |
| SMb20054 | **2.46** | *cpo* | COG0596R | putative non-heme chloroperoxidase protein |
| SMb20023 | **3.56** | *-* | COG0145EQ, COG0146EQ | hypothetical protein SM_b20023 |
| SMb20020 | **7.01** | *Pdh* | COG0022C, COG1071C | putative pyruvate dehydrogenase E1 component,alpha and beta subunits protein |
| SMa5013 | **2.21** |  | *-* | hypothetical protein |
| SMa2410 | **1.29** | *rhbF* | COG4264Q | RhbF rhizobactin siderophore biosynthesis protein RhsF |
| SMa2408 | **1.71** | *rhbE* | COG3486Q | RhbE rhizobactin siderophore biosynthesis protein |
| SMa2406 | **1.37** | *rhbD* | COG1670J | RhbD rhizobactin siderophore biosynthesis protein |
| SMa2389 | **2.52** | *-* | *-* | OsmC-like protein |
| SMa2339 | **1.20** | *-* | COG1670J | Siderophore biosynthesis protein |
| SMa2293 | **2.38** | *-* | COG0583K | beta lactamase transcriptional activator |
| SMa2245 | **1.77** | *-* | COG1199KL, COG1204R | hypothetical protein SMa2245 |
| SMa2243 | **2.19** | *-* | *-* | hypothetical protein SMa2243 |
| SMa2237 | **1.92** | *-* | *-* | hypothetical protein SMa2237 |
| SMa2233 | **2.10** | *-* | *-* | hypothetical protein SMa2233 |
| SMa2107 | **1.74** | *-* | COG0583K | GstR transcriptional regulator |
| SMa2101 | **2.51** | *-* | COG2141C | nitrilotriacetate monooxygenase component A |
| SMa2091 | **2.36** | *-* | COG1960I | hypothetical protein SMa2091 |
| SMa2089 | **3.66** | *-* | COG1960I | hypothetical protein SMa2089 |
| SMa2087 | **2.48** | *-* | COG0715P | desulfurization enzyme |
| SMa2077 | **4.65** | *-* | COG0667C | oxidoreductase |
| SMa2075 | **2.61** | *-* | COG0747E | extracellular solute-binding protein |
| SMa2073 | **2.90** | *-* | COG1960I | hypothetical protein SMa2073 |
| SMa1952 | **2.41** | *-* | COG2367V | putative Beta lactamase |
| SMa1919 | **1.84** | *-* | *-* | hypothetical protein SMa1919 |
| SMa1896 | **1.48** | *msrA3* | COG0225O | methionine sulfoxide reductase A |
| SMa1894 | **2.08** | *-* | COG0229O | methionine sulfoxide reductase B |
| SMa1798 | **2.48** | *kup2* | COG3158P | Kup2 potassium uptake protein |
| SMa1749 | **2.01** | *-* | COG2207K | putative transcriptional regulator |
| SMa1747 | **3.52** | *-* | COG1629P | TonB-dependent siderophore receptor |
| SMa1745 | **3.64** | *-* | COG0609P | ABC transporter, permease, Fe3+-siderophore transport system |
| SMa1741 | **3.43** | *-* | COG1120PH | ABC transporter, ATP-binding protein |
| SMa1718 | **2.04** | *adeC4* | COG1001F | AdeC4 adenine deaminase |
| SMa1705 | **1.93** | *-* | *-* | MUCR family transcriptional regulatory protein IN SYRB 5'region |
| SMa1697 | **1.74** | *-* | *-* | hypothetical protein SMa1697 |
| SMa1654 | **2.57** | *-* | COG1802K | GntR family transcriptional regulator |
| SMa1641 | **4.85** | *-* | COG0477GEPR | NreB protein |
| SMa1639 | **4.36** | *-* | *-* | hypothetical protein SMa1639 |
| SMa1626 | **3.96** | *-* | COG3803S | hypothetical protein SMa1626 |
| SMa1625 | **1.98** | *-* | COG0583K | LysR family transcriptional regulator |
| SMa1600 | **2.28** | *-* | COG1226P, COG4651P | Potassium efflux protein |
| SMa1585 | **1.73** | *-* | *-* | hypothetical protein SMa1585 |
| SMa1296 | **3.17** | *adhA1* | COG1064R | Alcohol dehydrogenase, Zn-dependent class III |
| SMa1283 | **5.60** | *nnrU* | COG4094S | NnrU family protein |
| SMa1279 | **7.87** | *norE* | COG1845C | NorE accessory protein for nitric oxide reductase |
| SMa1273 | **5.36** | *norB* | COG3256P | NorB nitric oxide reductase, large subunit |
| SMa1272 | **3.60** | *norQ* | COG0714R | NorQ accessory protein for nitric oxide reductase |
| SMa1266 | **5.47** | *hemN* | COG0635H | coproporphyrinogen III oxidase |
| SMa1264 | **2.48** | *-* | *-* | hypothetical protein SMa1264 |
| SMa1256 | **2.51** | *-* | COG0425O | hypothetical protein SMa1256 |
| SMa1252 | **3.01** | *nnrS* | COG3213P | NnrS family protein |
| SMa1243 | **9.64/2.88** | *azu1* | COG3794C | Azu1 pseudoazurin |
| SMa1241 | **1.71** | *napE* | COG4459C | NapE component of periplasmic nitrate reductase |
| SMa1240 | **2.96** | *napF* | COG1145C | NapF ferredoxin component of periplasmic nitrate reductase |
| SMa1231 | **3.93** | *-* | COG0589T | hypothetical protein SMa1231 |
| SMa1226 | **3.00** | *fixT1* | *-* | FixT1 inhibitor of FixL autophosphorylation |
| SMa1220 | **6.99** | *fixN1* | COG3278O | FixN1 cytochrome c oxidase subunit 1 |
| SMa1216 | **18.50** | *fixO1* | COG2993C | FixO1 cytochrome C oxidase subunit |
| SMa1214 | **37.22** | *fixQ1* | *-* | FixQ1 nitrogen fixation protein |
| SMa1213 | **28.18** | *fixP1* | COG2010C | FixP1 di-heme cytochrome c |
| SMa1211 | **5.38** | *fixG* | COG0348C | FixG iron sulfur membrane protein |
| SMa1210 | **10.08** | *fixH* | *-* | FixH nitrogen fixation protein |
| SMa1209 | **24.35** | *fixI1* | COG2217P | FixI1 ATPase |
| SMa1208 | **10.60** | *fixS1* | COG3197P | FixS1 nitrogen fixation protein |
| SMa1207 | **3.14** | *-* | *-* | Transcriptional regulator, CAP/Crp family |
| SMa1201 | **4.41** | *-* | *-* | hypothetical protein SMa1201 |
| SMa1200 | **4.39** | *-* | *-* | hypothetical protein SMa1200 |
| SMa1198 | **3.38** | *-* | COG1276P, COG2372R | copper export protein |
| SMa1191 | **2.60** | *fhp* | COG1017C, COG1018C | nitric oxide dioxygenase |
| SMa1188 | **10.66** | *nosX* | COG1477H | NosX accesory protein |
| SMa1186 | **5.27** | *nosL* | COG4314C | NosL copper chaperone |
| SMa1185 | **8.45** | *nosY* | COG1277R | NosY permease |
| SMa1183 | **12.37** | *nosD* | COG3420P | NosD nitrous oxidase accessory protein |
| SMa1182 | **24.25** | *nosZ* | COG4263C | nitrous-oxide reductase |
| SMa1179 | **13.76** | *nosR* | COG0348C, COG3901K | NosR regulatory protein for N2O reductase |
| SMa1178 | **2.96** | *-* | *-* | hypothetical protein SMa1178 |
| SMa1176 | **3.67** | *-* | *-* | hypothetical protein SMa1176 |
| SMa1170 | **7.73** | *cycB2* | COG2010C | hypothetical protein SMa1170 |
| SMa1169 | **5.93** | *-* | *-* | hypothetical protein SMa1169 |
| SMa1168 | **5.48** | *-* | COG2249R | Dehydrogenase, FAD-dependent |
| SMa1166 | **7.40** | *-* | COG0596R | putative hydrolase protein |
| SMa1163 | **3.71** | *-* | *-* | Cation transport P-type ATPase |
| SMa1158 | **5.23** | *-* | *-* | hypothetical protein SMa1158 |
| SMa1156 | **7.00** | *-* | COG1063ER | alcohol dehydrogenase |
| SMa1155 | **7.09** | *-* | COG0474P | cation transport P-type ATPase |
| SMa1154 | **8.00** | *-* | **-** | hypothetical protein SMa1154 |
| SMa1153 | **3.62** | *-* | COG2823R | hypothetical protein SMa1153 |
| SMa1151 | **2.24** | *-* | COG3467R | hypothetical protein SMa1151 |
| SMa1149 | **11.49** | *-* | COG0589T | hypothetical protein SMa1149 |
| SMa1147 | **4.11** | *-* | COG0589T | hypothetical protein SMa1147 |
| SMa1136 | **4.45/2.48** | *-* | *-* | hypothetical protein SMa1136 |
| SMa1134 | **5.60** | *-* | *-* | hypothetical protein SMa1134 |
| SMa1132 | **3.35/2.77** | *-* | *-* | hypothetical protein SMa1132 |
| SMa1131 | **6.51** | *-* | COG1236J | Metallo-beta-lactamase superfamily protein |
| SMa1128 | **5.54** | *degP4* | *-* | DegP4 protease like protein |
| SMa1126 | **16.39** | *-* | COG0517R, COG1994R | Protease |
| SMa1120 | **5.30** | *-* | COG1136V | ABC transporter ATP-binding protein |
| SMa1118 | **6.77** | *hspC2* | COG0071O | HspC2 heat shock protein |
| SMa1100 | **3.57** | *-* | *-* | hypothetical protein SMa1100 |
| SMa1095 | **2.02** | *-* | COG0589T | hypothetical protein SMa1095 |
| SMa1093 | **6.76** | *-* | *-* | hypothetical protein SMa1093 |
| SMa1092 | **3.00** | *-* | *-* | hypothetical protein SMa1092 |
| SMa1091 | **7.49** | *-* | *-* | hypothetical protein SMa1091 |
| SMa1089 | **6.06** | *-* | *-* | hypothetical protein SMa1089 |
| SMa1087 | **5.54** | *-* | COG2217P | putative cation transport P-type ATPase |
| SMa1086 | **4.79** | *-* | COG0517R | hypothetical protein SMa1086 |
| SMa1084 | **7.87** | *-* | COG3957G | putative phosphoketolase |
| SMa1082 | **3.08** | *-* | *-* | hypothetical protein SMa1082 |
| SMa1081 | **1.95** | *-* | *-* | hypothetical protein SMa1081 |
| SMa1079 | **3.20** | *tspO* | COG3476T | TspO/MBR family protein |
| SMa1078 | **2.38** | *-* | COG2155S | hypothetical protein SMa1078 |
| SMa1077 | **2.28** | *nex18* | COG2335M | Nex18 symbiotically induced protein |
| SMa1041 | **2.68** | *-* | COG4454P | copper oxidase |
| SMa1033 | **1.54** | *-* | *-* | hypothetical protein SMa1033 |
| SMa0981 | **2.41** | *ntrR2* | COG1487R | NtrR2 transcription regulator |
| SMa0848 | **4.64** | *-* | *-* | hypothetical protein SMa0848 |
| SMa0769 | **5.72** | *fixP2* | COG2010C | FixP2 Diheme c-type cytochrome |
| SMa0767 | **11.54/3.57** | *fixQ2* | *-* | FixQ2 nitrogen fixation protein |
| SMa0766 | **7.25** | *fixO2* | COG2993C | FixO2 cytochrome c oxidase subunit |
| SMa0765 | **9.76** | *fixN2* | COG3278O | FixN2 cytochrome c oxidase subunit I |
| SMa0760 | **3.30** | *fixT2* | COG0745TK, COG0784T | FixT2 anti-kinase protein |
| SMa0748 | **2.41** | *-* |  | Transcriptional regulator, MucR family |
| SMa0745 | **19.27** | *groES2* | COG0234O | co-chaperonin GroES |
| SMa0744 | **18.28/13.69** | *groEL2* | COG0459O | chaperonin GroEL |
| SMa0738 | **1.34** | *cspA6* | COG1278K | CspA6 cold shock protein transcriptional regulator |
| SMa0719 | **3.01** | *-* | COG1028IQR | short chain alcohol dehydrogenase-related dehydrogenase |
| SMa0715 | **2.06** | *-* | COG0451MG | Nucleoside-diphosphate-sugar epimerase |
| SMa0697 | **3.66** | *arcC* | COG0549E | carbamate kinase |
| SMa0695 | **3.55** | *arcB* | COG0078E | ornithine carbamoyltransferase |
| SMa0693 | **5.19** | *arcA1* | COG2235E | arginine deiminase |
| SMa0690 | **9.49** | *-* | *-* | hypothetical protein SMa0690 |
| SMa0689 | **10.75** | *-* | *-* | hypothetical protein SMa0689 |
| SMa0687 | **2.22** | *-* | *-* | hypothetical protein SMa0687 |
| SMa0682 | **8.28** | *-* | *-* | decarboxylase (lysine, ornithine, arginine) |
| SMa0680 | **11.22** | *-* | COG1982E | amino acid (ornithine, lysine, arginine) decarboxylase, |
| SMa0678 | **4.73** | *-* | COG0531E | putrescine transporter |
| SMa0673 | **3.80** | *-* | COG3328L | TRm3 transposase |
| SMa0670 | **5.48** | *-* | COG2326S | regulatory protein |
| SMa0669 | **3.00** | *-* | COG1566V | HlyD-family protein |
| SMa0662 | **4.56** | *-* | COG0664T | Transcriptional regulator, CAP/Crp family |
| SMa0636 | **4.48** | *-* | COG0517R | hypothetical protein SMa0636 |
| SMa0633 | **5.54** | *-* | *-* | hypothetical protein SMa0633 |
| SMa0631 | **6.77** | *-* | *-* | hypothetical protein SMa0631 |
| SMa0630 | **3.79** | *-* | COG0668M | hypothetical protein SMa0630 |
| SMa0520 | **2.79** | *-* | COG1737K | Transcriptional regulator, RpiR family |
| SMa0436 | **2.05** | *-* | *-* | hypothetical protein SMa0436 |
| SMa0412 | **2.90** | *-* | *-* | hypothetical protein SMa0412 |
| SMa0357 | **2.13** | *-* | COG3153R | hypothetical protein SMa0357 |
| SMa0355 | **1.93** | *-* | *-* | Transcriptional regulator, LysR family |
| SMa0307 | **2.01** | *-* | COG0583K | Transcriptional regulator, LysR family |
| SMa0291 | **4.28** | *-* | *-* | hypothetical protein SMa0291 |
| SMa0286 | **1.86** | *-* | *-* | hypothetical protein SMa0286 |
| SMa0252 | **2.30** | *-* | COG1638G | TRAP-type periplasmic solute-binding protein |
| SMa0250 | **2.78** | *-* | COG1593G | dedA-like protein |
| SMa0249 | **2.38** | *-* | COG3090G | TRAP-type small permease component |
| SMa0247 | **3.22** | *-* | COG3970R | hypothetical protein SMa0247 |
| SMa0128 | **1.69** | *-* | *-* | hypothetical protein SMa0128 |
| SMa0125 | **3.47** | *groES3* | COG0234O | co-chaperonin GroES |
| SMa0124 | **3.37** | *groEL3* | COG0459O | chaperonin GroEL |
| SMa0121 | **1.92** | *-* | *-* | hypothetical protein SMa0121 |
| SMa0045 | **2.55** | *cah* | COG3338P | carbonic anhydrase, Cah |
| SMa0036 | **3.01** | *-* | COG0488R | ABC transporter, ATP-binding protein |
