# Supplementary material for: Absence of functional TolC protein causes increased stress response gene expression in Sinorhizobium meliloti
Source: BMC Microbiol. 2010 Jun 23;10:180. doi: 10.1186/1471-2180-10-180 (PMC2912261; doi:10.1186/1471-2180-10-180)
Supplement: Additional file 2 — Genes with decreased expression in the S. meliloti tolC mutant. Table S2. Complete list of all S. meliloti SmLM030-2 genes with decreased expression (>1.2-fold change; p < 0.017) compared to expression in the wild-type S. meliloti 1021. Genes classified into COGs are the ones analyzed. [file 1471-2180-10-180-S2.DOC]

### **Additional file 2 - Table S2. Genes with decreased expression in the S. meliloti tolC mutant.**

Complete list of all *S. meliloti* SmLM030-2 genes with decreased expression (>1.2-fold change; p<0.017) compared to the expression in the wild-type *S. meliloti* 1021. The genes classified into COGs are the ones analyzed.

| **ID code** | **RMA FC** | **Gene** | **COG** | **Annotation** |
| --- | --- | --- | --- | --- |
| SMc04449 | **-1.52** | *cpdB* | COG0737F | bifunctional 2',3'-cyclic nucleotide 2'-phosphodiesterase/3'-nucleotidase periplasmic precursor protein |
| SMc04439 | **-1.56** | *-* | COG4175E | putative glycine betaine transport ATP-binding ABC transporter protein |
| SMc04432 | **-1.87** | *-* | COG3514S | hypothetical protein SMc04432 |
| SMc04431 | **-1.77** | *-* | COG2929S | hypothetical protein SMc04431 |
| SMc04404 | **-1.28** | *-* | COG1280E | putative amino acid efflux transmembrane protein |
| SMc04385 | **-1.57** | *-* | COG1012C | putative aldehyde dehydrogenase transmembrane protein |
| SMc04383 | **-3.61** | *-* | COG5383S | hypothetical protein SMc04383 |
| SMc04352 | **-1.66** | *-* | COG3672S | putative signal peptide protein |
| SMc04323 | **-7.78** | *-* | COG1167KE | aminotransferase protein |
| SMc04322 | **-1.51** | *mocA* | COG0667C | putative oxidoreductase protein |
| SMc04312 | **-1.72** | *-* | *-* | hypothetical protein SMc04312 |
| SMc04298 | **-1.52** | *-* | *-* | hypothetical protein SMc04298 |
| SMc04293 | **-2.22** | *-* | COG0747E | putative peptide-binding periplasmic ABC transporter |
| SMc04292 | **-2.04** | *cyaF3* | COG0457R, COG2114T | adenylate/guanylate cyclase protein |
| SMc04291 | **-3.25** | *-* | COG2133G | putative L-sorbosone dehydrogenase (SNDH) protein |
| SMc04280 | **-4.40** | *-* | *-* | putative signal peptide protein |
| SMc04241 | **-1.10** |  | COG0663R | Putative transferase hexapeptide repeat containing protein |
| SMc04232 | **-1.34** | *-* | *-* | glycine rich transmembrane protein |
| SMc04227 | **-1.81** | *mcpV* | COG0840NT | chemoreceptor methyl-accepting chemotaxis transmembrane protein |
| SMc04226 | **-1.69** | *-* | *-* | lipoprotein transmembrane |
| SMc04222 | **-2.40** | *-* | COG4453S | hypothetical protein SMc04222 |
| SMc04221 | **-1.59** | *-* | COG0454KR | hypothetical protein SMc04221 |
| SMc04212 | **-1.60** | *-* | COG0642T | putative sensor histidine kinase transmembrane protein |
| SMc04206 | **-2.79** | *-* | COG2931Q | putative hemolysin-type calcium-binding protein |
| SMc04205 | **-2.22** | *-* | COG1629P | putative IRON/HEME transport protein |
| SMc04194 | **-2.64** | *-* | *-* | putative transmembrane protein |
| SMc04165 | **-1.76** | *-* | COG0596R | putative oxidoreductase protein |
| SMc04153 | **-8.73** | *-* | COG0404E | putative aminomethyltransferase protein |
| SMc04152 | **-6.06** | *-* | *-* | hypothetical protein SMc04152 |
| SMc04150 | **-3.33** | *-* | *-* | hypothetical protein SMc04150 |
| SMc04149 | **-4.46** | *-* | COG2072P | putative oxidoreductase protein |
| SMc04147 | **-10.73** | *-* | COG0531E | putative permease protein |
| SMc04127 | **-1.51** | *-* | COG3845R | ABC transporter ATP-binding protein |
| SMc04126 | **-1.87** | *-* | COG4603R | putative transport system permease ABC transporter protein |
| SMc04125 | **-1.69** | *-* | COG1079R | putative transport system permease ABC transporter protein |
| SMc04124 | **-1.53** | *cdd* | COG0295F | cytidine deaminase |
| SMc04118 | **-2.76** | *-* | *-* | hypothetical protein SMc04118 |
| SMc04117 | **-2.95** | *-* | *-* | hypothetical protein SMc04117 |
| SMc04115 | **-1.61** | *-* | COG4964U | hypothetical protein SMc04115 |
| SMc04114 | **-2.35** | *pilA1* | COG3847U | putative pilin subunit protein |
| SMc04113 | **-2.44** | *cpaA1* | COG4960OU | putative pilus assembly transmembrane protein |
| SMc04056 | **-1.57** | *-* | COG0667C | putative oxidoreductase protein |
| SMc04049 | **-3.67** | *-* | COG2041R | putative sulfite oxidase protein |
| SMc04048 | **-3.18/-3.15** | *-* | COG2010C | putative cytochrome c protein |
| SMc04047 | **-2.37** | *azu2* | COG3794C | pseudoazurin (blue copper protein) |
| SMc04037 | **-2.35** | *-* | COG0747E | peptide ABC transporter |
| SMc04032 | **-3.65** | *nesR* | COG2771K | putative transcription regulator protein |
| SMc04028 | **-11.74** | *gltB* | COG0067E, COG0069E, COG0070E | glutamate synthase NADPH large chain protein |
| SMc04026 | **-9.20** | *gltD* | COG0493ER | glutamate synthase subunit beta |
| SMc04024 | **-2.04** | *-* | COG2951M | putative membrane-bound lytic murein transglycosylase protein |
| SMc04018 | **-1.70** | *-* | COG0737F, COG1652S | 5'-nucleotidase precursor (signal peptide) protein |
| SMc04011 | **-3.81** | *tacA* | COG2204T | putative sigma-54-dependent transcription regulator protein |
| SMc03999 | **-1.81** | *-* | *-* | hypothetical protein SMc03999 |
| SMc03880 | **-1.76** | *aniA* | COG5394S | hypothetical protein SMc03880 |
| SMc03879 | **-2.05** | *phbA* | COG0183I | acetyl-CoA acetyltransferase |
| SMc03844 | **-1.82** | *-* | *-* | hypothetical protein SMc03844 |
| SMc03807 | **-8.10** | *amtB* | COG0004P | ammonium transporter |
| SMc03806 | **-9.09** | *glnK* | COG0347E | nitrogen regulatory protein PII 2 |
| SMc03246 | **-1.89** | *-* | COG0582L | putative integrase DNA protein |
| SMc03208 | **-5.46** | *hmgA* | COG3508Q | homogentisate 1,2-dioxygenase |
| SMc03207 | **-1.89** | *-* | COG0179Q | putative aromatic amino acid degradation protein |
| SMc03174 | **-1.69** | *-* | *-* | hypothetical protein SMc03174 |
| SMc03131 | **-1.31** | *-* | COG0834ET | putative amino acid-binding periplasmic ABC transporter protein |
| SMc03090 | **-2.04** | *cheW3* | COG3415L | putative chemotaxis protein |
| SMc03066 | **-1.99** | *-* | *-* | hypothetical protein SMc03066 |
| SMc03037 | **-1.62** | *flaA* | COG1344N | flagellin A protein |
| SMc02884 | **-1.76** | *-* | COG1744R | putative lipoprotein precursor |
| SMc02873 | **-1.89** | *-* | COG1653G | putative periplasmic binding (signal peptide) ABC transporter protein |
| SMc02848 | **-2.29** | *-* | *-* | hypothetical protein SMc02848 |
| SMc02832 | **-1.74** | *-* | COG4166E | putative periplasmic binding protein |
| SMc02770 | **-1.99** | *-* | COG4553I | Putative intracellular PHB depolymerase |
| SMc02738 | **-1.48** | *chow* | COG4176E | putative glycine betaine transport system permease ABC transporter protein |
| SMc02737 | **-2.32** | *choX* | COG2113E | putative glycine betaine-binding ABC transporter protein |
| SMc02689 | **-1.93** | *-* | COG1012C | aldehyde dehydrogenase |
| SMc02685 | **-2.32** | *-* | *-* | hypothetical protein SMc02685 |
| SMc02620 | **-1.83** | *-* | COG1396K | hypothetical protein SMc02620 |
| SMc02619 | **-2.49** | *-* | *-* | hypothetical protein SMc02619 |
| SMc02618 | **-2.00** | *-* | *-* | hypothetical protein SMc02618 |
| SMc02613 | **-4.61** | *glnT* | COG0174E | glutamine synthetase III protein |
| SMc02612 | **-3.17** | *glxD* | COG0069E | putative oxidoreductase protein |
| SMc02611 | **-3.49** | *glxC* | COG0070E | putative oxidoreductase protein |
| SMc02610 | **-4.43** | *glxB* | COG0067E | putative amidotransferase protein |
| SMc02602 | **-9.34** | *-* | *-* | hypothetical protein SMc02602 |
| SMc02489 | **-2.11** | *xerC* | COG0582L | site-specific tyrosine recombinase XerC |
| SMc02461 | **-2.01** | *-* | COG3549R | hypothetical protein SMc02461 |
| SMc02447 | **-1.79** | *-* | *-* | hypothetical protein SMc02447 |
| SMc02446 | **-1.54** | *-* | COG3847U | hypothetical protein SMc02446 |
| SMc02394 | **-1.91** | *-* | COG3093R | hypothetical protein SMc02394 |
| SMc02378 | **-2.48** | *-* | COG2113E | putative periplasmic binding transmembrane protein |
| SMc02356 | **-2.44** | *-* | COG0683E | branched chain amino acid ABC transporter periplasmic protein |
| SMc02340 | **-2.49** | *-* | COG2188K | putative transcription regulator protein |
| SMc02339 | **-2.58** | *-* | COG1028IQR | putative oxidoreductase protein |
| SMc02326 | **-1.75** | *-* | *-* | hypothetical protein SMc02326 |
| SMc02260 | **-1.67** | *-* | COG4598E | ABC transporter ATP-binding protein |
| SMc02259 | **-3.16** | *-* | COG0834ET | putative periplasmic binding ABC transporter protein |
| SMc02258 | **-1.44** | *-* | COG4215E | putative transport system permease ABC transporter protein |
| SMc02257 | **-1.64** | *-* | COG4160E | putative transport system permease ABC transporter protein |
| SMc02250 | **-1.91** | *mscL* | COG1970M | large-conductance mechanosensitive channel transmembrane protein |
| SMc02240 | **-26.96** | *-* | COG1284S | hypothetical protein SMc02240 |
| SMc02239 | **-2.41** | *-* | COG2128S | hypothetical protein SMc02239 |
| SMc02229 | **-2.08** | *-* | COG1960I | putative acyl-CoA dehydrogenase protein |
| SMc02228 | **-2.20** | *fadA* | COG0183I | acetyl-CoA acetyltransferase |
| SMc02227 | **-2.59** | *fadB* | COG1024I, COG1250I | putative fatty oxidation complex alpha subunit Includes: enoyl-COA hydratase, 3-hydroxyacyl-COA dehydrogenase, 3-hydroxybutyryl-COA epimerase transmembrane protein |
| SMc02121 | **-1.85** | *aapP* | COG1126E | general L-amino acid transport ATP-binding ABC transporter protein |
| SMc02120 | **-2.85** | *aapM* | COG0765E | general L-amino acid transport permease ABC transporter protein |
| SMc02119 | **-3.18** | *aapQ* | COG4597E | general L-amino acid transport permease ABC transporter protein |
| SMc02118 | **-1.85** | *aapJ* | COG0834ET | general L-amino acid-binding periplasmic ABC transporter protein |
| SMc02111 | **-1.56** | *-* | *-* | hypothetical protein SMc02111 |
| SMc02073 | **-1.92** | *argS* | COG0018J | arginyl-tRNA synthetase |
| SMc02060 | **-1.70** | *lppB* | COG0739M, COG0741M | lipoprotein precursor |
| SMc02049 | **-2.26** | *gcvP* | COG0403E, COG1003E | glycine dehydrogenase |
| SMc02048 | **-1.83** | *gcvH* | COG0509E | glycine cleavage system protein H |
| SMc01982 | **-1.75** | *coxM* | COG1622C | alternative cytochrome C oxidase polypeptide II transmembrane protein |
| SMc01981 | **-2.34** | *-* | COG3474C | putative cytochrome C transmembrane protein |
| SMc01973 | **-3.19** | *-* | COG0174E | glutamine synthetase |
| SMc01972 | **-2.24** | *ordL1* | COG0665E | putative oxidoreductase protein |
| SMc01967 | **-18.73/-2.99** | *speB2* | COG0010E | putative agmatinase protein |
| SMc01966 | **-12.44/-4.58** | *-* | COG0687E | spermidine/putrescine ABC transporter periplasmic protein |
| SMc01965 | **-7.36** | *-* | COG3842E | spermidine/putrescine ABC transporter ATP-binding protein |
| SMc01964 | **-5.84** | *-* | COG1176E | spermidine/putrescine ABC transporter permease |
| SMc01963 | **-5.19** | *-* | COG1177E | spermidine/putrescine ABC transporter permease |
| SMc01962 | **-4.94** | *-* | COG0388R | hypothetical protein SMc01962 |
| SMc01961 | **-2.63** | *-* | COG0346E | hypothetical protein SMc01961 |
| SMc01946 | **-2.01** | *livK* | COG0683E | putative leucine-specific binding protein precursor |
| SMc01940 | **-1.56** | *-* | COG3813S | hypothetical protein SMc01940 |
| SMc01872 | **-1.36** | *ftsQ* | COG1589M | cell division transmembrane protein |
| SMc01871 | **-1.67** | *ddl* | COG1181M | D-alanine--D-alanine ligase |
| SMc01857 | **-1.63** | *-* | COG2001S | cell division protein MraZ |
| SMc01826 | **-4.92** | *-* | COG0600P | Putative uracil and uridine ABC transporter, permease component |
| SMc01825 | **-3.23** | *-* | COG0600P | Putative uracil and uridine ABC transporter, permease component |
| SMc01824 | **-2.44** | *-* | *-* | hypothetical protein SMc01824 |
| SMc01823 | **-3.30** | *-* | *-* | Putative uracil and uridine ABC transporter, ATP-binding component |
| SMc01822 | **-3.34** | *-* | COG4101G | hypothetical protein SMc01822 |
| SMc01820 | **-12.71** | *-* | COG0624E | allantoate amidohydrolase |
| SMc01819 | **-10.02** | *-* | COG1309K | putative transcription regulator protein |
| SMc01818 | **-2.24** | *cyaC* | COG0633C, COG2114T | putative adenylate cyclase transmembrane protein |
| SMc01814 | **-12.51** | *-* | COG0493ER | putative oxidoreductase |
| SMc01813 | **-10.50/-2.89** | *-* | COG3568R | hypothetical protein SMc01813 |
| SMc01768 | **-2.48** | *-* | COG1846K | putative transcription regulator protein |
| SMc01719 | **-2.83** | *mcpT* | COG0840NT | chemoreceptor methyl-accepting chemotaxis transmembrane protein |
| SMc01666 | **-1.36** | *mdeA* | COG0626E | methionine gamma-lyase |
| SMc01656 | **-2.52** | *-* | COG1012C | gamma-aminobutyraldehyde dehydrogenase |
| SMc01645 | **-2.17** | *-* | COG1123R | putative ABC transporter ATP-binding protein |
| SMc01644 | **-1.89** | *-* | COG1173EP | ABC transporter permease |
| SMc01643 | **-2.73** | *-* | COG0601EP | ABC transporter permease |
| SMc01642 | **-4.17** | *-* | *-* | putative periplasmic binding protein |
| SMc01632 | **-2.62** | *-* | COG0687E | putative periplasmic binding ABC transporter protein |
| SMc01615 | **-1.96** | *-* | COG1349KG | putative transcription regulator protein |
| SMc01614 | **-1.98** | *tpiA* | COG0149G | triosephosphate isomerase |
| SMc01613 | **-2.13** | *rpiB* | COG0698G | ribose-5-phosphate isomerase B |
| SMc01608 | **-1.84** | *-* | COG3842E | ABC transporter ATP-binding protein |
| SMc01607 | **-2.23** | *-* | COG1177E | putative permease protein |
| SMc01606 | **-4.17** | *-* | COG4132R | putative permease protein |
| SMc01605 | **-3.74** | *-* | COG1840P | putative periplasmic binding ABC transporter protein |
| SMc01602 | **-2.77** | *-* | COG1737K | hypothetical protein SMc01602 |
| SMc01600 | **-1.78** | *-* | COG3931E | hypothetical protein SMc01600 |
| SMc01597 | **-8.09** | *-* | COG0531E | putative amino-acid permease protein |
| SMc01594 | **-4.87** | *-* | COG0174E | Putative glutamine synthetase |
| SMc01592 | **-2.59** | *-* | COG1846K | putative transcription regulator protein |
| SMc01588 | **-2.33** | *-* | COG1012C | putative aldehyde dehydrogenase protein |
| SMc01582 | **-2.05** | *-* | COG1454C | putative alcohol dehydrogenase protein |
| SMc01579 | **-1.72** | *-* | COG1192D | hypothetical protein SMc01579 |
| SMc01525 | **-2.01** | *dppA2* | COG0747E | putative dipeptide binding periplasmic protein |
| SMc01521 | **-1.36** | *ntrR1* | COG1487R | nitrogen regulatory protein |
| SMc01509 | **-2.33** | *-* | *-* | hypothetical protein SMc01509 |
| SMc01506 | **-4.72** | *rpoE2* | COG1595K | RNA polymerase sigma factor |
| SMc01505 | **-2.39** | *-* | *-* | Anti-sigma factor |
| SMc01504 | **-7.24** | *-* | COG0784T | two-component response regulator |
| SMc01493 | **-1.91** | *-* | COG1733K | hypothetical protein SMc01493 |
| SMc01490 | **-1.79** | *-* | *-* | hypothetical protein SMc01490 |
| SMc01467 | **-2.99** | *-* | *-* | putative signal peptide protein |
| SMc01460 | **-2.29** | *-* | COG2128S | hypothetical protein SMc01460 |
| SMc01459 | **-2.89** | *-* | COG1878R | hypothetical protein SMc01459 |
| SMc01432 | **-1.97** | *-* | COG0433R | hypothetical protein SMc01432 |
| SMc01416 | **-2.00** | *-* | COG4190K | hypothetical protein SMc01416 |
| SMc01374 | **-2.03** | *-* | COG1376S, COG3409M | hypothetical protein SMc01374 |
| SMc01267 | **-2.62** | *-* | COG2766T | hypothetical protein SMc01267 |
| SMc01266 | **-3.19** | *-* | COG2718S | hypothetical protein SMc01266 |
| SMc01265 | **-2.48** | *-* | COG2719S | hypothetical protein SMc01265 |
| SMc01241 | **-1.67** | *-* | *-* | hypothetical protein SMc01241 |
| SMc01237 | **-2.47** | *nrd* | COG0209F | ribonucleotide-diphosphate reductase subunit alpha |
| SMc01043 | **-6.86** | *ntrC* | COG2204T | nitrogen assimilation regulatory protein |
| SMc01042 | **-7.96** | *ntrB* | COG3852T | nitrogen regulation protein |
| SMc01041 | **-9.54/-4.43** | *dusB* | COG0042J | tRNA-dihydrouridine synthase B |
| SMc01029 | **-1.85** | *-* | COG2919D | hypothetical protein SMc01029 |
| SMc01022 | **-1.87** | *-* | COG3038C | putative cytochrome B transmembrane protein |
| SMc01021 | **-1.72** | *-* | COG2353S | hypothetical protein SMc01021 |
| SMc01017 | **-2.41** | *-* | COG0454KR | putative acetyltransferase protein |
| SMc01003 | **-1.48** | *-* | COG1752R | hypothetical protein SMc01003 |
| SMc00999 | **-1.90** | *-* | *-* | hypothetical protein SMc00999 |
| SMc00998 | **-3.82** | *-* | COG3672S | putative signal peptide protein |
| SMc00991 | **-1.78** | *-* | COG0687E | putative putrescine-binding periplasmic protein |
| SMc00987 | **-2.43** | *-* | *-* | hypothetical protein SMc00987 |
| SMc00980 | **-1.55** | *-* | COG3757M | hypothetical protein SMc00980 |
| SMc00948 | **-3.79** | *glnA* | COG0174E | glutamine synthetase I protein |
| SMc00947 | **-4.56** | *glnB* | COG0347E | nitrogen regulatory protein PII |
| SMc00939 | **-1.56** | *-* | COG3795S | hypothetical protein SMc00939 |
| SMc00928 | **-1.59** | *-* | COG3773M | putative signal peptide protein |
| SMc00924 | **-2.80** | *-* | COG1376S | hypothetical protein SMc00924 |
| SMc00922 | **-2.81** | *-* | COG1953FH | putative transporter transmembrane protein |
| SMc00885 | **-9.06** | *-* | *-* | putative transmembrane signal peptide protein |
| SMc00883 | **-1.55** | *-* | COG3386G | hypothetical protein SMc00883 |
| SMc00881 | **-1.56** | *dgoK1* | COG3734G | putative 2-dehydro-3-deoxygalactonokinase protein |
| SMc00849 | **-3.66** | *-* | *-* | hypothetical protein SMc00849 |
| SMc00824 | **-1.46** | *-* | COG5403S | hypothetical protein SMc00824 |
| SMc00823 | **-1.81** | *-* | COG5499K | hypothetical protein SMc00823 |
| SMc00795 | **-2.45** | *-* | *-* | hypothetical protein SMc00795 |
| SMc00786 | **-1.83** | *dppA1* | COG0747E | periplasmic dipeptide-binding protein |
| SMc00770 | **-1.71** | *potF* | COG0687E | putrescine-binding periplasmic protein |
| SMc00720 | **-2.34** | *-* | COG0784T | putative 2-component receiver domain protein |
| SMc00708 | **-1.55** | *glob* | COG0491R | putative hydroxyacylglutathione hydrolase (glyoxalase II) (GLX II) protein |
| SMc00672 | **-3.08** | *hisX* | COG2113E | histidine-binding periplasmic signal peptide protein |
| SMc00671 | **-1.67** | *hisW* | COG4176E | histidine transport system permease ABC transporter protein |
| SMc00667 | **-1.40** | *-* | COG3758S | hypothetical protein SMc00667 |
| SMc00657 | **-1.92** | *-* | *-* | hypothetical protein SMc00657 |
| SMc00655 | **-2.17** | *-* | *-* | hypothetical protein SMc00655 |
| SMc00654 | **-2.32** | *ctrA* | COG0745TK | response regulator,controls chromosomal replication initiation protein |
| SMc00653 | **-1.61** | *-* | COG0784T | putative 2-component receiver domain protein |
| SMc00652 | **-1.56** | *chpT* | COG0642T | putative histidine phosphotransferase |
| SMc00651 | **-1.82** | *-* | *-* | hypothetical protein SMc00651 |
| SMc00638 | **-2.45** | *-* | *-* | putative HEAT resistant agglutinin 1 protein |
| SMc00607 | **-1.52** | *-* | *-* | putative signal peptide protein |
| SMc00548 | **-1.50** | *-* | *-* | putative signal peptide protein |
| SMc00518 | **-1.45** | *-* | COG1975O | hypothetical protein SMc00518 |
| SMc00517 | **-1.53** | *-* | COG1975O | hypothetical protein SMc00517 |
| SMc00507 | **-2.68** | *-* | *-* | hypothetical protein SMc00507 |
| SMc00506 | **-1.38** | *-* | COG5473S | hypothetical protein SMc00506 |
| SMc00479 | **-1.31** | *-* | COG2207K | putative transcription regulator protein |
| SMc00456 | **-2.66** | *-* | *-* | hypothetical protein SMc00456 |
| SMc00371 | **-3.68** | *-* | COG3685S | hypothetical protein SMc00371 |
| SMc00347 | **-1.85** | *rnk* | COG0782K | nucleoside diphosphate kinase regulator |
| SMc00278 | **-4.38** | *-* | COG1522K | putative transcription regulator protein |
| SMc00277 | **-1.74** | *-* | COG0520E | hypothetical protein SMc00277 |
| SMc00242 | **-1.98** | *-* | *-* | putative signal peptide protein |
| SMc00170 | **-2.01** | *-* | COG2771K | putative transcription regulator HSL-dependent protein |
| SMc00159 | **-7.24** | *-* | COG4961U | putative signal peptide protein |
| SMc00158 | **-6.31** | *-* | COG4655S | hypothetical protein SMc00158 |
| SMc00140 | **-1.74** | *-* | COG0834ET | putative amino-acid binding periplasmic protein |
| SMc00137 | **-3.00** | *-* | *-* | hypothetical protein SMc00137 |
| SMc00136 | **-1.63** | *-* | COG1028IQR | putative oxidoreductase protein |
| SMc00101 | **-1.40** | *-* | COG0656R | putative oxidoreductase protein |
| SMc00084 | **-1.66** | *-* | COG1734T | hypothetical protein SMc00084 |
| SMc00059 | **-1.58** | *-* | COG0642T | putative sensor histidine kinase protein |
| SMc00049 | **-1.45** | *-* | COG1734T | hypothetical protein SMc00049 |
| SMc00041 | **-1.61** | *-* | *-* | hypothetical protein SMc00041 |
| SMc00040 | **-1.80** | *-* | COG1764O | hypothetical protein SMc00040 |
| SMc00030 | **-1.56** | *-* | *-* | hypothetical protein SMc00030 |
| SMc00021 | **-2.17** | *ccrM* | COG0863L | adenine DNA methyltransferase protein |
| SMc00003 | **-2.13** | *-* | COG2214O | putative chaperone protein |
| SMb21707 | **-14.39** | *-* | COG0410E | putative ureashort-chain amide or branched-chain amino acid uptake ABC transporter ATP-binding protein |
| SMb21683 | **-1.91** | *-* | *-* | hypothetical protein SM_b21683 |
| SMb21572 | **-4.17** | *-* | COG2113E | putative amino acid uptake ABC transporter periplasmic solute-binding protein precursor |
| SMb21570 | **-3.83** | *stcD2* | COG0446R, COG1902C | putative NADH-dependent oxidase protein |
| SMb21526 | **-2.01** | *tauA* | COG4521P | putative taurine uptake ABC transporter periplasmic solute-binding protein precursor |
| SMb21514 | **-1.57** | *hemK2* | COG2890J | putative modification methylase protein |
| SMb21502 | **-1.87** | *-* | COG0438M | putative glycosyltransferase protein |
| SMb21497 | **-1.42** | *acrE* | COG0845M | putative acriflavin resistance protein |
| SMb21481 | **-2.39** | *-* | COG0517R | hypothetical protein SM_b21481 |
| SMb21441 | **-1.96** | *-* | *-* | putative inosine-5'-monophosphate dehydrogenase protein |
| SMb21438 | **-2.73** | *-* | COG1638G | putative C4-dicarboxylate transport system, C4-dicarboxylate-binding protein precursor signal peptide |
| SMb21419 | **-1.53** | *-* | COG2207K | putative transcriptional regulator, araC family protein |
| SMb21377 | **-2.45** | *-* | COG1879G | putative sugar uptake ABC transporter periplasmic solute-binding protein precursor |
| SMb21330 | **-2.16** | *-* | *-* | hypothetical protein SM_b21330 |
| SMb21317 | **-2.75** | *wggR* | COG1846K | transcriptional activator of exopolysaccharide II synthesis, MarR family protein |
| SMb21250 | **-1.81** | *-* | COG0438M | putative glycosyltransferase protein |
| SMb21249 | **-1.32** | *-* | *-* | putative sulfotransferase protein |
| SMb21245 | **-1.73** | *exoF3* | COG1566V, COG1596M | OMA family outer membrane protein |
| SMb21240 | **-1.80** | *-* | COG0489D, COG3206M | putative MPA1 family protein |
| SMb21221 | **-2.65** | *-* | COG1653G | putative sugar uptake ABC transporter periplasmic solute-binding protein precursor |
| SMb21181 | **-2.15** | *-* | COG1960I | putative glutaryl-CoA dehydrogenase protein |
| SMb21166 | **-4.69** | *hutI* | *-* | imidazolonepropionase |
| SMb21165 | **-7.70** | *hutH* | COG2986E | histidine ammonia-lyase |
| SMb21164 | **-11.54** | *hutG* | COG3741E | putative formiminoglutamase protein |
| SMb21163 | **-10.29** | *hutU* | COG2987E | urocanate hydratase |
| SMb21152 | **-2.07** | *-* | COG3839G | putative sugar uptake ABC transporter ATP-binding protein |
| SMb21151 | **-3.46** | *-* | COG1653G | putative periplasmic solute-binding protein of a sugar uptake ABC transporter system |
| SMb21149 | **-1.60** | *-* | COG1175G | putative sugar uptake ABC transporter permease protein |
| SMb21148 | **-2.61** | *-* | COG0673R | putative oxidoreductase protein |
| SMb21117 | **-2.55** | *-* | COG1396K | putative transcriptional regulator protein |
| SMb21115 | **-20.15** | *-* | COG3707T | putative response regulator protein |
| SMb21114 | **-10.33** | *nrtA* | COG0715P | putative nitrate transport protein |
| SMb21098 | **-2.27** | *-* | COG2079R | hypothetical protein SM_b21098 |
| SMb21097 | **-17.52** | *-* | COG0834ET | putative amino acid uptake ABC transporter periplasmic solute-binding protein precursor |
| SMb21096 | **-10.66** | *-* | *-* | putative amino acid transporter ABC transporter ATP-binding protein |
| SMb21095 | **-10.33** | *-* | COG0765E | putative amino acid uptake ABC transporter permease protein |
| SMb21094 | **-20.73** | *argH2* | COG0165E | argininosuccinate lyase |
| SMb21052 | **-1.70** | *-* | COG0451MG | epimerase dehydratase |
| SMb21001 | **-1.83** | *-* | *-* | hypothetical protein SM_b21001 |
| SMb20993 | **-1.73** | *-* | COG1960I | putative FMNH2-dependent monooxygenase protein |
| SMb20992 | **-1.42** | *-* | COG4529S | hypothetical protein SM_b20992 |
| SMb20987 | **-7.63** | *-* | COG0007H, COG1648H | putative siroheme synthetase protein |
| SMb20986 | **-14.10** | *narB* | COG0243C | putative nitrate reductase, large subunit protein |
| SMb20985 | **-26.57** | *nirD* | COG2146PR | putative nitrite reductase |
| SMb20984 | **-22.74** | *nirB* | COG1251C | putative nitrite reductase |
| SMb20960 | **-1.58** | *exoN* | COG1210M | UDP-glucose pyrophosphorylase |
| SMb20941 | **-2.30** | *exsA* | COG1132V | MsbA-like saccharide exporting ABC transporter ATP-binding/permease subunits |
| SMb20933 | **-1.95** | *exsG* | COG2202T, COG2203T, COG3920T | putative two-component sensor histidine kinase protein |
| SMb20911 | **-1.68** | *-* | *-* | hypothetical protein SM_b20911 |
| SMb20910 | **-3.57** | *-* | *-* | hypothetical protein SM_b20910 |
| SMb20909 | **-3.18** | *-* | *-* | hypothetical protein SM_b20909 |
| SMb20888 | **-3.28** | *-* | COG1305E | hypothetical protein SM_b20888 |
| SMb20887 | **-4.77** | *-* | COG2307S | hypothetical protein SM_b20887 |
| SMb20886 | **-4.54** | *-* | COG2308S | hypothetical protein SM_b20886 |
| SMb20879 | **-1.53** | *-* | *-* | hypothetical protein SM_b20879 |
| SMb20848 | **-1.73** | *-* | COG3748S | membrane protein |
| SMb20754 | **-1.60** | *-* | COG1396K, COG3800R | hypothetical protein SM_b20754 |
| SMb20745 | **-3.86** | *glnII* | COG0174E | putative glutatmine synthetase II protein |
| SMb20686 | **-2.27** | *-* | COG1273S | putative ATP-dependent DNA ligase protein |
| SMb20649 | **-1.50** | *nadE* | COG0171H | NAD synthetase |
| SMb20647 | **-4.23** | *-* | *-* | hypothetical protein SM_b20647 |
| SMb20605 | **-22.36** | *-* | COG0683E | putative ureashort-chain amide or branched-chain amino acid uptake ABC transporter periplasmic solute-binding protein precursor |
| SMb20604 | **-24.97** | *-* | COG0559E | short-chain amide/branched-chain amino acid uptake ABC transporter permease |
| SMb20603 | **-15.70** | *-* | COG4177E | putative ureashort-chain amide or branched-chain amino acid uptake ABC transporter permease protein |
| SMb20602 | **-11.94** | *-* | COG4674R | putative ureashort-chain amide or branched-chain amino acid uptake ABC transporter ATP-binding protein |
| SMb20600 | **-1.74** | *-* | *-* | hypothetical protein SM_b20600 |
| SMb20597 | **-2.48/-2.43** | *-* | COG2259S | hypothetical protein SM_b20597 |
| SMb20592 | **-1.40** | *rpoE8* | COG1595K | RNA polymerase sigma factor |
| SMb20590 | **-1.46** | *-* | COG1254C | hypothetical protein SM_b20590 |
| SMb20562 | **-1.33** | *-* | COG3660M | hypothetical protein SM_b20562 |
| SMb20552 | **-1.59** | *-* | COG4099R | hypothetical protein SM_b20552 |
| SMb20475 | **-1.45** | *-* | COG3865S | hypothetical protein SM_b20475 |
| SMb20464 | **-3.49** | *-* | *-* | hypothetical protein SM_b20464 |
| SMb20436 | **-63.48/-62.15** | *-* | COG2223P | putative nitrate transporter protein |
| SMb20383 | **-2.19** | *-* | COG0687E | putative ABC transporter periplasmic solute-binding protein |
| SMb20340 | **-1.44** | *-* | *-* | hypothetical protein SM_b20340 |
| SMb20316 | **-2.23** | *-* | COG1879G | putative ABC transporter periplasmic sugar-binding protein |
| SMb20292 | **-1.88** | *-* | COG2358R | immunogenic protein |
| SMb20277 | **-1.60** | *-* | COG0161H | hypothetical protein SM_b20277 |
| SMb20261 | **-1.45** | *-* | COG2055C | putative malate dehydrogenase protein |
| SMb20154 | **-1.70** | *-* | COG1609K | putative transcriptional regulator protein |
| SMb20121 | **-1.69** | *-* | COG3609K | hypothetical protein SM_b20121 |
| SMb20119 | **-1.49/-1.45** | *-* | COG1961L | hypothetical protein SM_b20119 |
| SMb20099 | **-3.40** | *-* | COG0366G | maltose alpha-D-glucosyltransferase |
| SMb20097 | **-2.92** | *-* | COG0277C | oxidoreductase |
| SMb20079 | **-2.79** | *-* | COG2931Q | putative hemolysin-adenlyate cyclase protein |
| SMb20055 | **-1.75** | *-* | COG4977K | putative transcriptional regulator protein |
| SMb20046 | **-1.37** | *repA1* | COG1192D | replication protein A |
| SMb20025 | **-1.77** | *-* | COG3181S | hypothetical protein SM_b20025 |
| SMb20022 | **-1.58** | *-* | COG0412Q | hypothetical protein SM_b20022 |
| SMb20021 | **-2.02** | *-* | COG1522K | putative transcriptional regulator protein |
| SMa5007 | **-2.17** | *-* | *-* | Possible transcriptional regulator |
| SMa5002 | **-6.39** | *-* | *-* | Hypothetical protein of ISRm2011-2, orfC C-terminus |
| SMa2379 | **-2.13** | *katB* | COG0376P | KatB catalase/peroxidase |
| SMa2361 | **-4.11** | *-* | COG1744R | hypothetical protein SMa2361 |
| SMa2257 | **-1.69** | *-* | COG0582L | integrase-like protein |
| SMa2255 | **-2.10** | *-* | *-* | hypothetical protein SMa2255 |
| SMa2253 | **-3.31** | *-* | COG3744S | hypothetical protein SMa2253 |
| SMa2069 | **-1.53** | *cysP1* | COG1613P | ABC transporter |
| SMa2037 | **-1.40** | *-* | *-* | Oxidoreductase |
| SMa1990 | **-1.75** | *-* | COG4226S | hypothetical protein SMa1990 |
| SMa1961 | **-1.58** | *-* | *-* | polyhydroxybutyrate depolymerase |
| SMa1867 | **-1.49** | *-* | COG1522K | AsnC family transcriptional regulator |
| SMa1862 | **-1.54** | *-* | COG0601EP | ABC transporter permease |
| SMa1860 | **-2.07** | *-* | COG0747E | ABC transporter periplasmic solute-binding protein |
| SMa1821 | **-2.08** | *-* | *-* | hydrolase |
| SMa1820 | **-1.73** | *-* | COG4317S | hypothetical protein SMa1820 |
| SMa1793 | **-1.90** | *-* | *-* | hypothetical protein SMa1793 |
| SMa1766 | **-2.51** | *-* | COG2261S | hypothetical protein SMa1766 |
| SMa1765 | **-2.89** | *-* | COG3753S | hypothetical protein SMa1765 |
| SMa1761 | **-1.82** | *-* | COG0160E | Aminotransferase |
| SMa1755 | **-2.42** | *-* | COG0687E | ABC transporter periplasmic solute-binding protein |
| SMa1683 | **-4.96** | *-* | COG3119P | Arylsulfatase |
| SMa1682 | **-2.98** | *-* | *-* | nonspecific acid phosphatase precursor |
| SMa1670 | **-2.35** | *arcA2* | COG2235E | arginine deiminase |
| SMa1635 | **-1.72** | *-* | *-* | hypothetical protein SMa1635 |
| SMa1597 | **-1.46** | *-* | COG3121NU | Pilus assembly chaperone |
| SMa1462 | **-1.77** | *-* | COG1732M | ABC transporter |
| SMa1452 | **-3.57** | *-* | COG1028IQR | hypothetical protein SMa1452 |
| SMa1450 | **-3.31** | *-* | COG0183I | thiolase |
| SMa0943 | **-2.00** | *-* | COG3119P | arylsulfatase |
| SMa0825 | **-2.00** | *nifH* | COG1348P | nitrogenase reductase |
| SMa0822 | **-2.36** | *fixA* | COG2086C | FixA electron transfer flavoprotein beta chain |
| SMa0815 | **-1.99** | *nifA* | *-* | NifA transcriptional activator |
| SMa0616 | **-1.47** | *fixQ3* | *-* | FixQ3 nitrogen fixation protein |
| SMa0615 | **-1.59** | *fixO3* | COG2993C | FixO3 cytochrome-c oxidase subunit |
| SMa0612 | **-1.84** | *fixN3* | COG3278O | FixN3 cytochrome c oxidase subunit 1 |
| SMa0592 | **-1.59** | *-* | COG3550R | hypothetical protein SMa0592 |
| SMa0585 | **-34.77** | *nrtA* | COG0715P | nitrate ABC transporter substrate-binding protein |
| SMa0583 | **-33.00** | *nrtB* | COG0600P | nitrate ABC transporter permease |
| SMa0581 | **-24.80** | *nrtC* | COG1116P | nitrate transport ATP binding protein, |
| SMa0548 | **-2.38** | *-* | COG4691R | hypothetical protein SMa0548 |
| SMa0545 | **-1.93** | *-* | COG4113R | hypothetical protein SMa0545 |
| SMa0506 | **-1.61** | *-* | COG0834ET | ABC transporter periplasmic substrate-binding protein |
| SMa0403 | **-4.73** | *-* | *-* | hypothetical protein SMa0403 |
| SMa0402 | **-8.40** | *-* | COG2188K | Transcriptional regulator, GntR family |
| SMa0400 | **-4.75** | *-* | SMa0400 | Dehydrogenase, Zn-dependent |
| SMa0398 | **-10.61** | *hisD2* | *-* | histidinol dehydrogenase |
| SMa0396 | **-10.09** | *-* | COG1177E | ABC transporter, permease |
| SMa0394 | **-10.47** | *-* | COG1176E | ABC transporter, permease |
| SMa0392 | **-23.52/-8.28** | *-* | COG0687E | ABC transporter, periplasmic solute-binding protein |
| SMa0391 | **-15.58** | *-* | COG3842E | ABC transporter, ATP-binding protein |
| SMa0389 | **-3.00** | *-* | *-* | short chain alcohol dehydrogenase-related dehydrogenase |
| SMa0387 | **-11.44** | *hisC3* | COG0079E | histidinol-phosphate aminotransferase |
| SMa0325 | **-1.67** | *-* | COG1359S | hypothetical protein SMa0325 |
| SMa0319 | **-2.04** | *-* | COG2207K | Transcriptional regulator, AraC family |
| SMa0316 | **-5.41** | *-* | COG0599S | hypothetical protein SMa0316 |
| SMa0314 | **-6.76** | *-* | *-* | hypothetical protein SMa0314 |
| SMa0312 | **-8.43** | *-* | *-* | hypothetical protein SMa0312 |
| SMa0144 | **-1.71** | *-* | *-* | hypothetical protein SMa0144 |
| SMa0142 | **-3.18** | *-* | COG1404O | protease |
| SMa0134 | **-4.13** | *-* | *-* | hypothetical protein SMa0134 |
| SMa0104 | **-3.16** | *-* | COG0747E | ABC transporter, periplasmic solute-binding protein |
| SMa0093 | **-1.75** | *-* | COG0115EH | Branched-chain amino acid aminotransferase |
