# Supplementary material for: Absence of functional TolC protein causes increased stress response gene expression in Sinorhizobium meliloti
Source: BMC Microbiol. 2010 Jun 23;10:180. doi: 10.1186/1471-2180-10-180 (PMC2912261; doi:10.1186/1471-2180-10-180)
Supplement: Additional file 3 — Primer sequences used in this study. Table S3. Gene-specific primers used for real-time RT-PCR. [file 1471-2180-10-180-S3.DOC]

### **Additional file 3 - Table S3. Primer sequences used in this study.**

Gene-specific primers used for real-time RT-PCR.

| Gene identification nº | Gene name | Forward primer | Reverse primer |
| --- | --- | --- | --- |
| SMc03104 | *hemA* | GGCAAGGAATCGGCTCTTC | TGCGGCCCAGTTCGAA |
| SMc03167 | *-* | TGGCGGTCATCAACACTATCC | AGCCGGGCGTAGTGGAA |
| SMc00948 | *glnA* | GCCGGCGACGAGTATGC | TGATGATGCCACCGATGAAG |
| SMc03046 | *rem* | CGCGATCTACGGCATCTTC | CTTGCTGATGTGGCTTTCGA |
| SMc01498 | *smoG* | GCGATCGCACCCATCCT | CGCGCACGAGCTGTTTTT |
| SMb21314 | *wgeA* | CCTGCAGATATCCAAGGGTATCA | GATACGCGAAGCGAGATCCT |
| SMc04028 | *gltB* | TCATTCGCCATCACGACAGA | GGGAATGCGGGCGTTT |
| SMb21094 | *argH2* | CTGGAGCTCTAAGCAGTTCAGTTG | CGAGGAGCCGGTGGAATA |
| SMa0585 | *nrtA* | GCAGATGGTCGCCAACATG | TTCGCCGACGCAGAAAC |
| SMc00090 | *cysN* | GGCCGAGCGGGAACAG | TTGTCGGTCGCGAAATAGC |
| SMc02365 | *degP1* | CCGCTGCGGCGTTTC | AACGATCGGCACGGTCAT |
